# Safety profile of sikamat virus and its oncolytic potential in leukemic cells and cancer stem cells

**DOI:** 10.1038/s41598-025-96061-z

**Published:** 2025-04-22

**Authors:** Zhen Yun Siew, Ghee Khang Ong, Siew Tung Wong, Pooi Pooi Leong, Boon Shing Tan, Chee-Onn Leong, Juita Binti Chupri, Chee-Mun Fang, Kenny Voon

**Affiliations:** 1 School of Pharmacy, University of Nottingham Malaysia, 43500 Semenyih, Selangor Malaysia; 2 School of Medicine, IMU University, Bukit Jalil, 57000 Kuala Lumpur, Malaysia; 3 Faculty of Medicine and Health Sciences, Universiti Tunku Abdul Rahman, 43000 Kajang, Selangor Malaysia; 4 AGTC Genomics, Bukit Jalil, 57000 Kuala Lumpur, Malaysia; 5 Department of Pathology, Faculty of Medicine and Health Sciences, Universiti Putra Malaysia, 43400 UPM Serdang, Selangor Malaysia

**Keywords:** Cancer stem cell, Myeloid leukaemia, Oncolytic virotherapy, Transcriptome analysis, *Pteropine orthoreovirus*, Oncolytic reovirus, Cancer stem cells, Cancer therapy, Haematological cancer, Cancer, Virology, Viral pathogenesis, Cancer stem cells, Pluripotent stem cells

## Abstract

Leukaemia remains a global health concern. The oncotherapy resistance of leukaemia might be due to the existence of cancer stem cell populations. This study investigated the therapeutic potential of Sikamat virus (PRV7S), a *Pteropine orthoreovirus*, as an oncolytic virus against acute myeloid leukaemia (AML) and chronic myeloid leukaemia (CML). Using AML and CML cell lines (THP-1 and K562), as well as an AML-M5-derived cancer stem cell (CSC) model, PRV7S was shown to infect these leukaemic cells, replicate within them, and reduce their viability. PRV7S-induced cell death was associated with caspase-mediated apoptosis without significant cell cycle arrest. Transcriptomic and proteomic analyses revealed that PRV7S infection altered several cell death pathways, including apoptosis and necroptosis, highlighting its complex cell death mechanisms. PRV7S replicated efficiently in infected cells, though it did not cause persistent infection. An in vivo safety evaluation in immunocompetent mice demonstrated that PRV7S was well-tolerated, showing no adverse effects on survival, body weight, or histopathology, and no evidence of viral persistence. These findings suggest PRV7S as a promising oncolytic candidate for myeloid leukaemia, with potential efficacy against CSCs and a favourable safety profile. In conclusion, the study provides new insights into the cellular pathways involved in PRV7S-mediated oncolysis and supports further exploration of PRV7S’s potential against resistant leukaemic and solid tumours.

## Introduction

Leukaemia represents a group of life-threatening malignancies affecting the blood and bone marrow. In the United States, acute myeloid leukaemia (AML) has been recorded as the highest incidence and mortality in 2019 among other types of leukaemia, reaching more than twenty-thousand newly diagnosed cases and nearly eleven-thousand deaths^1^. The overall trajectory is rising in the United States, intriguingly, the incidence rate of AML in males is double that in females^2^. In contrast, myelogenous leukaemia was the most prevalent in Malaysia from 2012 to 2016, the incidence rate between both genders is similar^3^. Epidemiological studies reveal a higher incidence and mortality rate of leukaemia in males compared to females^4–6^. Furthermore, leukaemia imposes a substantial burden on paediatric populations. According to CANCER TODAY under the World Health Organization (WHO)^7^, the latest data on global cancer incidence and mortality rates is only available up to 2022. Leukemia ranked 13th in overall incidence and 10th in mortality. China, India, and the United States of America (USA) were the top three countries with the highest leukaemia incidence and mortality rates. Furthermore, in Asia alone, leukaemia incidence and mortality were reported at 227,206 (46.6%) and 158,144 (51.8%), respectively, contributing to approximately half of the global leukaemia cases and associated deaths^7^ (Supplementary Fig. 1).

Apart from AML, chronic myeloid leukaemia (CML) is a distinguished classification of myeloproliferative neoplastic disorder arising from myeloid progenitor cells and is commonly defined with the association of the positive Philadelphia (Ph) chromosome. Ph chromosome formation results from the translocation between the q34 segment of chromosome 9 and the q11 segment of chromosome 22, with the cytogenetic abbreviation of t(9;22)(q34;q11)^8,9^. Both AML and CML were initially classified according to the French-American-British (FAB) system, subsequently revised and refined by the WHO recommendations (Supplementary Tables 1–4).

Several risk factors have been implicated in the development of leukaemia, including a history of pre-leukaemic blood disorders such as myelodysplastic syndrome, prior chemotherapy or radiation therapy, exposure to ionising radiation or carcinogenic chemicals like benzene, and genetic predispositions^4,6,10^. Conventional therapeutic modalities, such as chemotherapy and radiation therapy, have not demonstrated the highest efficacy in the permanent eradication of myeloid leukaemia and may paradoxically elevate the risk of leukaemia^4,10,11^. This underscores the urgent need for the development and implementation of alternative therapeutic strategies for leukaemia on a global scale.

The concept of cancer stem cells (CSCs), also known as tumour-initiating cells (TICs), was first introduced in AML in 1997^12^. Since then, CSCs have also been identified in CML^13,14^. CSCs are notably resistant to chemotherapy and are key drivers of cancer recurrence and patient mortality. Thus, targeting CSCs remains one of the paramount challenges in contemporary oncology^15,16^.

A breakthrough was achieved with the successful generation of an induced pluripotent stem cell (iPSC) model from the AML-M5 subtype, THP1^17^. We hypothesise that this iPSC model closely resembles CSCs that may exist within a minor population of leukaemic cells. Therefore, a treatment effective against this iPSC model could potentially be effective against other CSCs as well.

Oncolytic viruses (OVs), also known as oncolytic vaccines, are viral particles with a unique affinity for tumor cells, inducing cytopathic effects (CPE) and direct tumour oncolysis either intrinsically or through genetic modification. Additionally, an oncolytic vaccine must have the capability to stimulate an immune response that ultimately benefits the cancer patient. T-VEC, the first oncolytic virotherapy approved by the Food and Drug Administration (FDA), is used to treat high-grade melanoma and has significantly contributed to the advancement and success of OV research both in vitro and in vivo. Besides, several reoviruses isolated from different organisms have also demonstrated oncolytic properties, including *Mammalian orthoreovirus* (MRV), *Avian orthoreovirus* (ARV), and *Pteropine orthoreovirus* (PRV). Orthoreovirus type 3 Dearing (Pelareorep, Reolysin), a subtype of MRV, is the most well-established oncolytic reovirus. Despite completing phase III trials, Reolysin has not yet received FDA approval but has been designated an orphan drug for the treatment of ovarian cancer, pancreatic cancer, and malignant glioma. Additionally, a significant drawback of most OVs is their reliance on the efficacy of chemotherapies and other cancer treatments to achieve desirable outcomes in various clinical trials across different cancers (Supplementary Tables 5, 6)^18^.

In this study, we aim to examine the therapeutic potential of a PRV member, Sikamat virus (PRV7S, NCBI ID: txid1109732), as an oncolytic reovirus. We investigated its oncolytic activities, cell death mechanisms in myeloid leukaemia cells (THP1 and K562), and associated safety concerns. Interestingly, an AML-M5-derived CSC model was also studied.

### Leukemic cells are susceptible to PRV7S

**Fig. 1:**
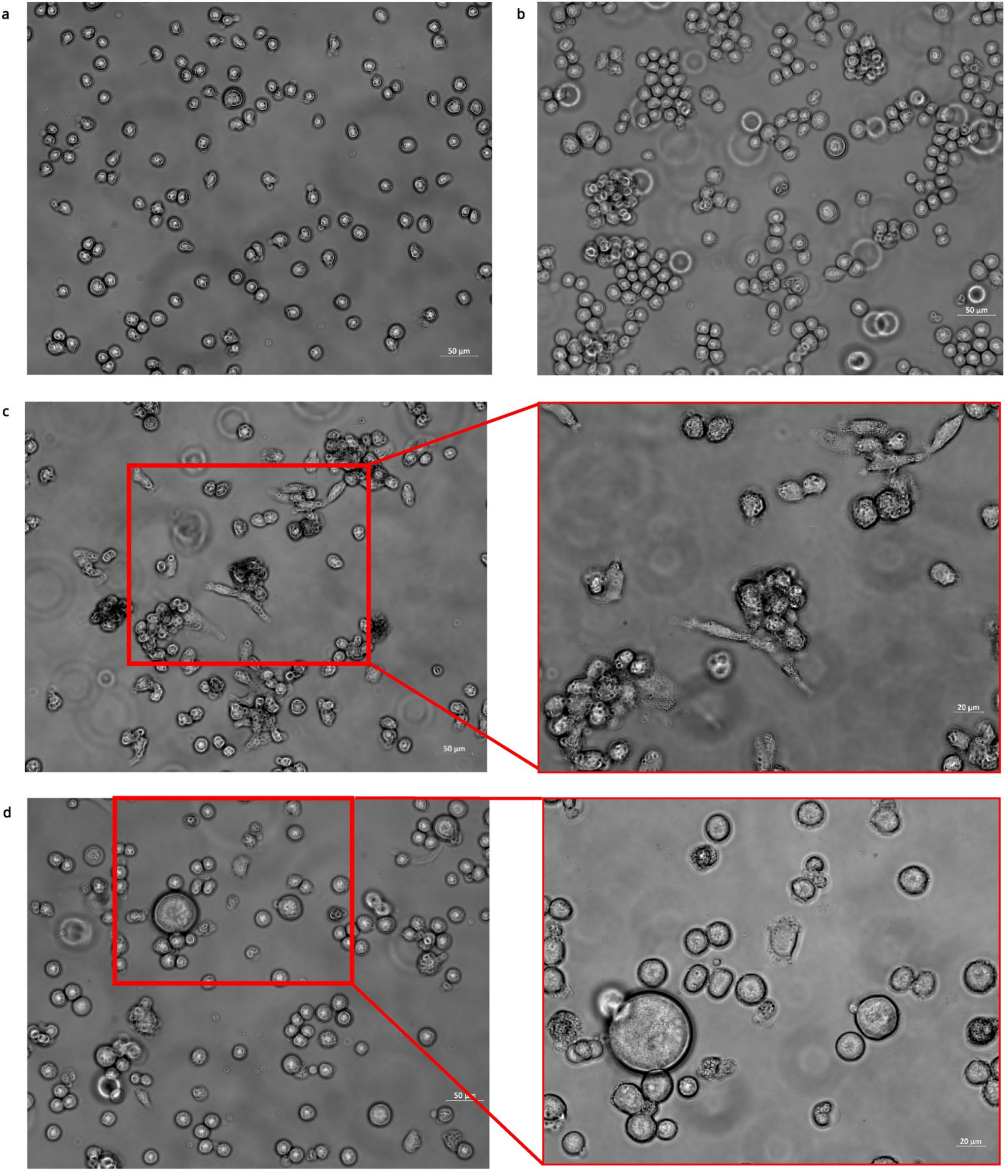

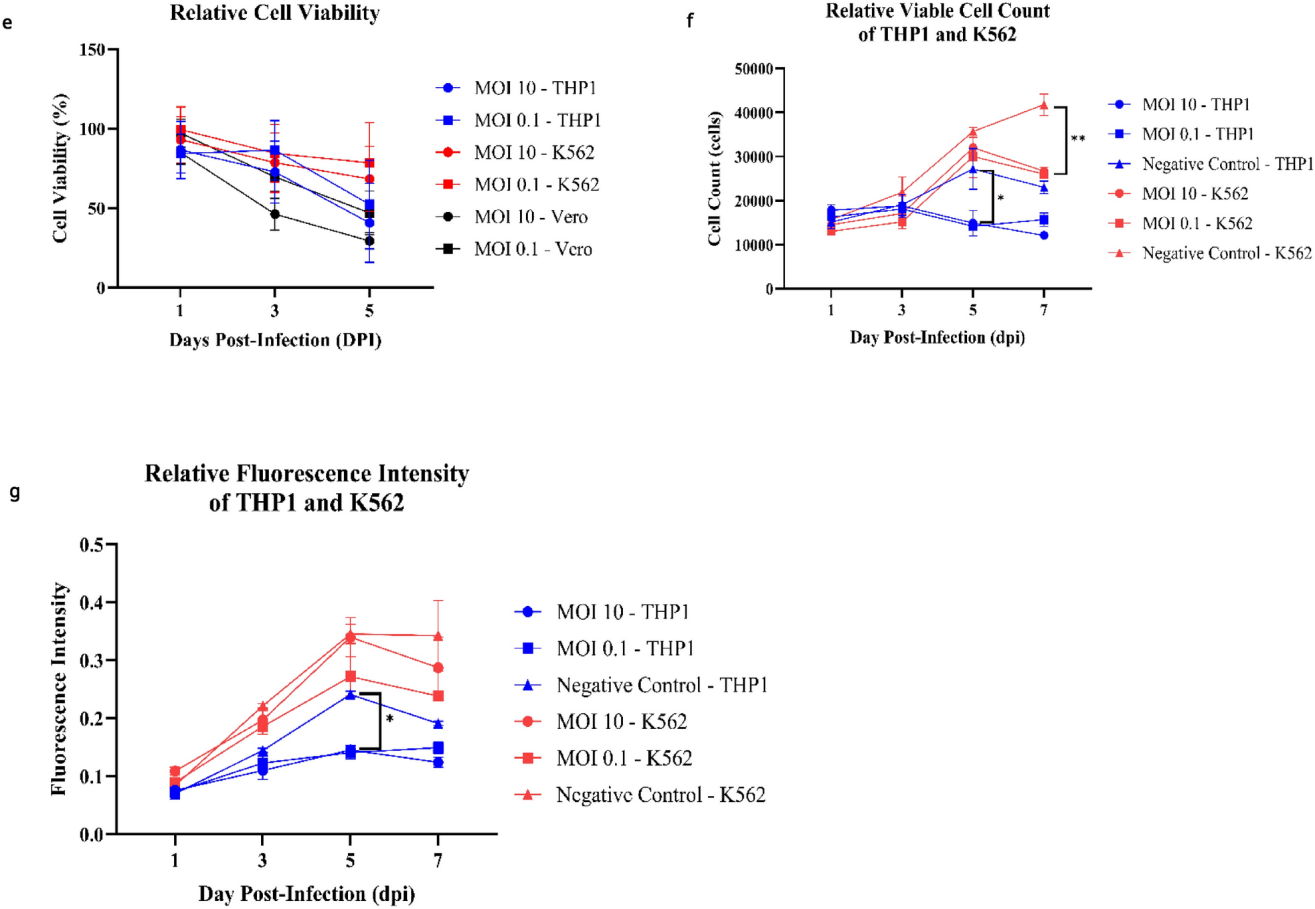
THP1 and K562 cells are susceptible to PRV7S infection. (**a**,**b**) Representative micrograph of non-infected THP1 and K562 cells. Scale bar: 50 μm. (**c**) PRV7S-infected THP1 cells appeared elongated and spindle-like at 5 days post-infection (dpi). Some infected THP1 cells turned adhesive and attached to the well bottom. Scale bar: 50 μm (left), 20 μm (right). (**d**) Plasma membrane blebbing and cell lysis were observed in PRV7S-infected K562 cells at 5 dpi. Scale bar: 50 μm (left), 20 μm (right). (**e**) Cell viability of PRV7S-infected THP1 and K562 cells at MOI 10, 0.1 was measured relative to Vero cells as the standard propagation cell line for PRV7S. (**f**,**g**) Cell viability validation assay using trypan blue (**f**) and PI (**g**). All data directly (**f**) and indirectly (**e**,**g**) represent the cell viability of PRV7S-infected THP1 and K562 cells compared to non-infected cells. (**e**–**g**) Data is presented with mean ± SD. A two-way ANOVA analysis was performed. *p < 0.05; **p < 0.01.

Morphological alterations, including cell elongation, membrane blebbing, and lysis, were observed in infected cell lines (Fig. 1c,d) relative to uninfected controls (Fig. 1a,b), although the CPE was less pronounced in K562 cells (Fig. 1d). Additionally, increased cell clumping was noted in both infected cell lines. In the case of THP1 cells, PRV7S infection led to some cells becoming adherent to the well bottom (Fig. 1c). To evaluate the susceptibility of leukaemia cells to PRV7S, cell viability assays were conducted using a multi-well plate format. Acute myeloid leukaemia (THP1) and chronic myeloid leukaemia (K562) cell lines were assessed for susceptibility to PRV7S using the 3-(4,5-Dimethylthiazol-2-yl)-2,5-diphenyltetrazolium bromide (MTT) assay (Fig. 1e), with further validation by trypan blue (Fig. 1f) and propidium iodide (PI) assays (Fig. 1g). PRV7S-infected THP1 and K562 cells exhibited generally reduced viability compared to uninfected cells, with a time-dependent decrease in viability (Fig. 1e). This reduction in viability was further corroborated by trypan blue and PI staining assays (Fig. 1f,g).

### PRV7S propagates without causing persistent infection

**Fig. 2:**
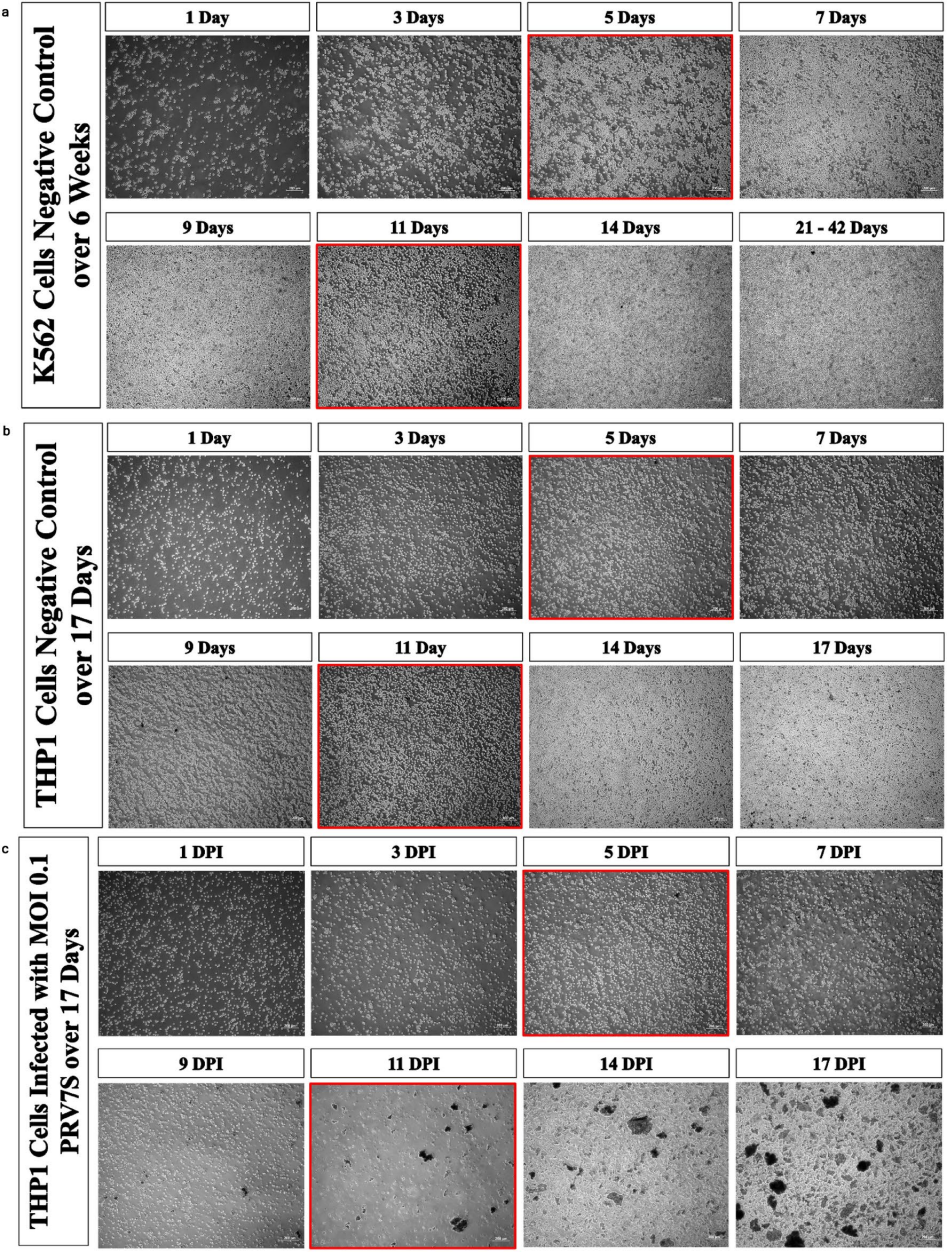

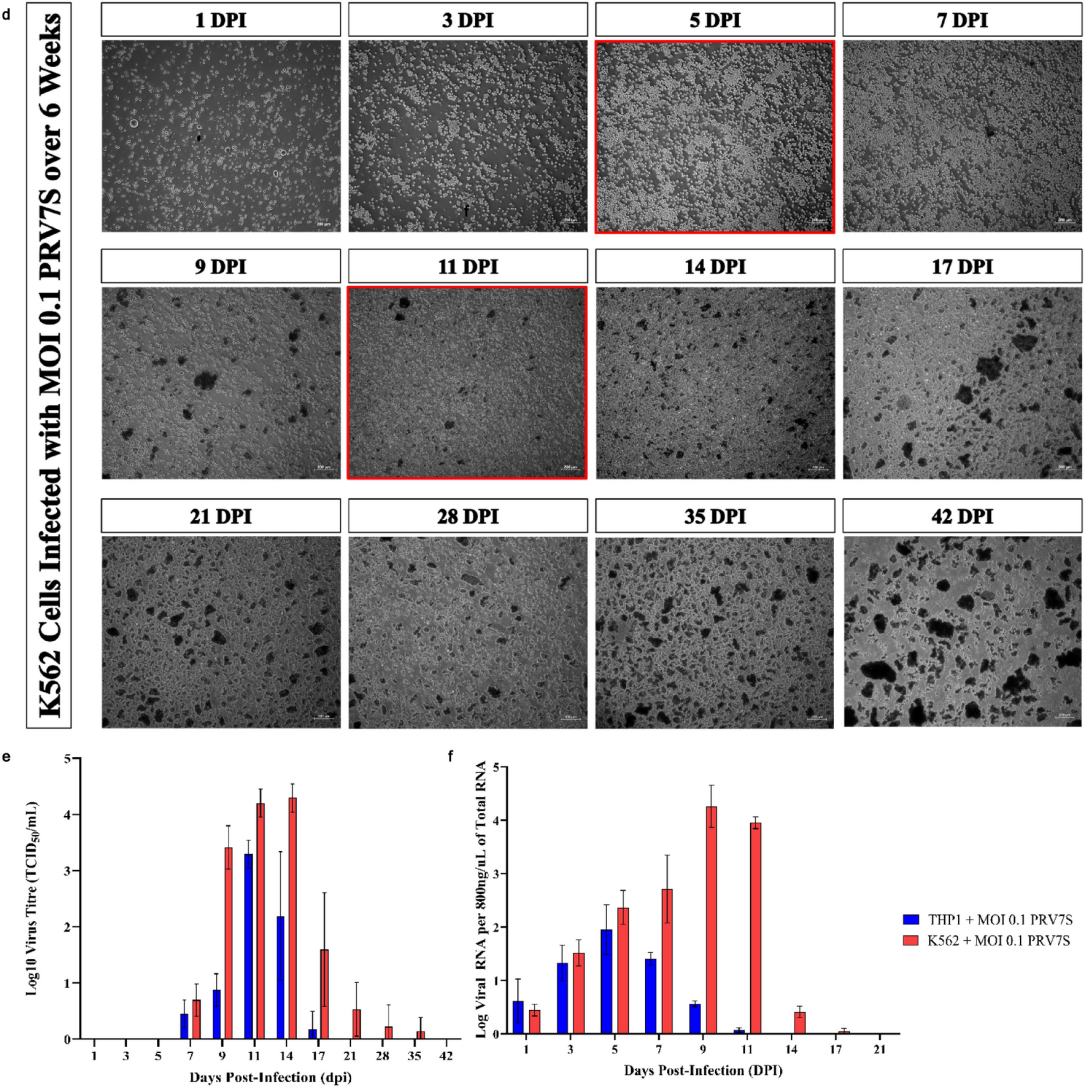
PRV7S propagates without causing persistent infection. (**a**–**d**) Representative micrographs of infected and non-infected THP1 and K562 cells over time. The complete medium was refreshed at 5 and 11 dpi. Scale bar: 200 μm. (**e**) Extracellular virus titre of PRV7S was determined using end-point dilution TCID_50_ assay. (**f**) Intracellular viral RNA of PRV7S was measured using qRT-PCR. (**e**,**f**) Data are presented as mean ± SD. Four independent experiments were performed, each with four replicates.

The replication kinetics of PRV7S in THP1 and K562 cells were investigated using the 50% tissue culture infectious dose (TCID_50_) assay to measure extracellular viral titre, and quantitative real-time polymerase chain reaction (qRT-PCR) to quantify intracellular viral RNA. CPE in infected cells served as a microscopic indicator of viral replication, as viral egress induces cell death, thereby releasing progeny virions from the intracellular to the extracellular environment^18,19^. Cell debris and fragments accumulated gradually, while cell confluency declined in infected THP1 and K562 cells over time. By the final day of observation, cell debris and fragments were notably prominent compared to the negative controls (Fig. 2a–d). Interestingly, multinucleated syncytia, a hallmark of fusogenic PRV7S^20^, were rarely observed in infected THP1 and K562 suspension cells (Fig. 2b,d). However, cell clumping observed in Fig. 1c,d may represent virus-induced cell fusion similar to syncytium formation. Notably, syncytiogenesis in infected cells facilitates viral propagation and replication, enhancing progeny production^21^.

Correlation between the TCID_50_ and qRT-PCR assays indicated that PRV7S infected both cell lines by 1 dpi and generated a detectable titre of viral progeny by 7 dpi. Intracellular viral RNA levels initially increased but subsequently declined over time, becoming undetectable by 21 dpi as most infected cells were lysed (Fig. 2e,f). This suggests that PRV7S does not establish persistent infection in THP1 and K562 cells, consistent with previous observations in solid tumour cells^22^. Overall, K562 cells produced a higher titre of functional extracellular viral progeny and exhibited greater levels of intracellular viral RNA compared to THP1 cells (Fig. 2e,f). These findings imply that K562 cells are more permissive to PRV7S infection and support higher viral replication capacity than THP1 cells.

### PRV7S induces apoptosis and necrosis but does not cause cell cycle arrest

**Fig. 3:**
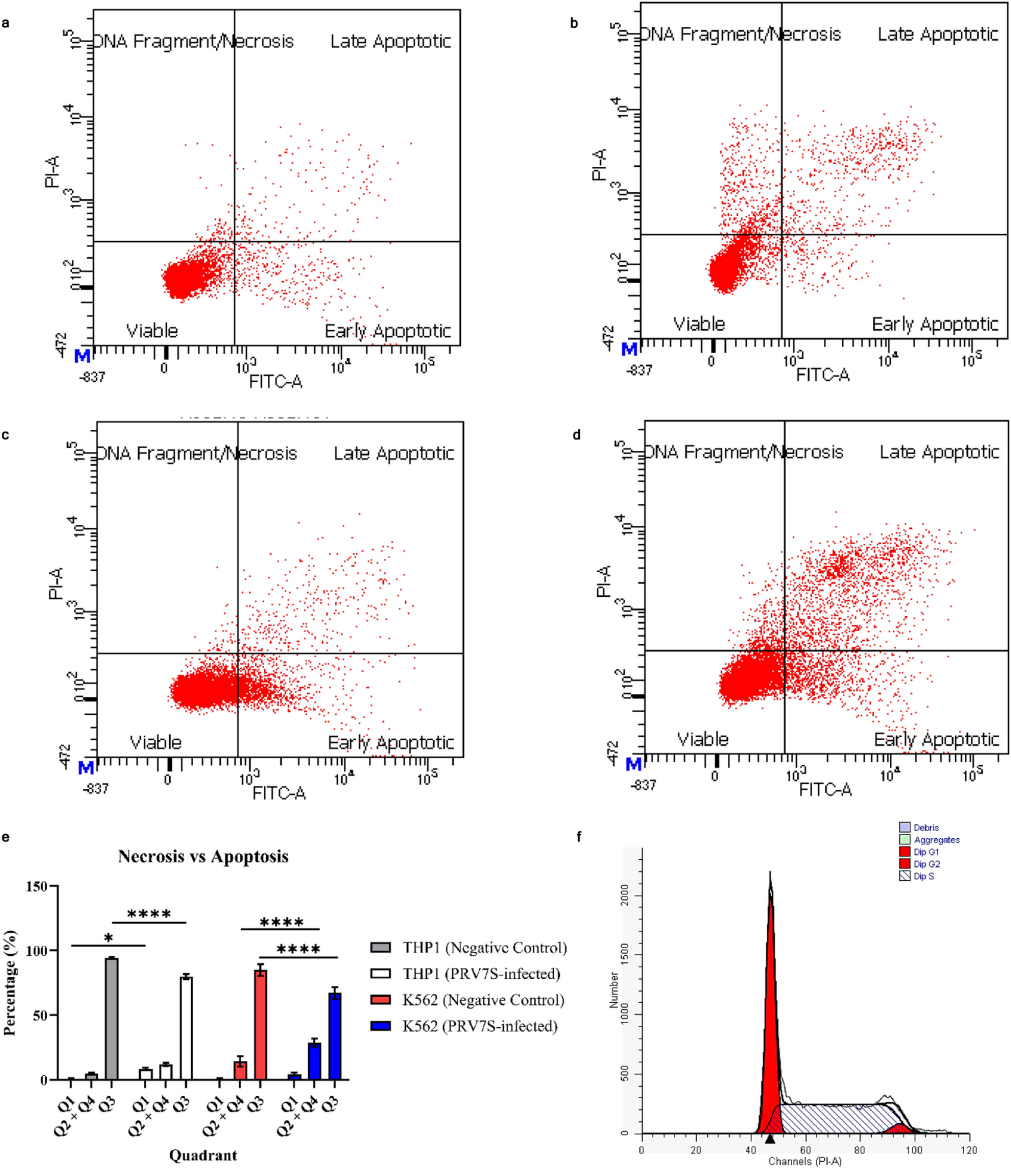

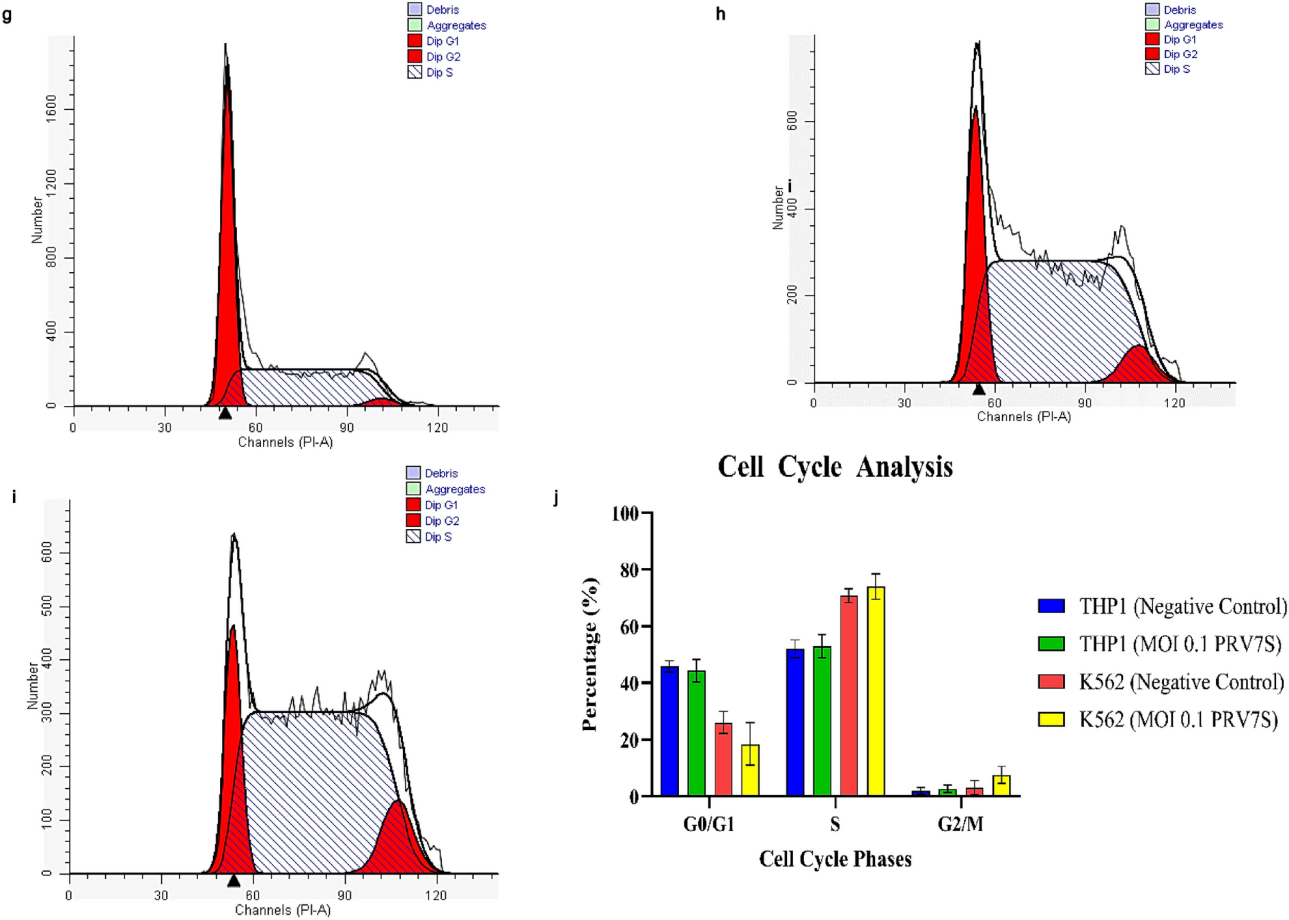
PRV7S induces apoptosis and necrosis but does not cause cell cycle arrest. (**a**–**d**) Flow cytometry analysis of non-infected THP1 (**a**), infected THP1 (**b**), non-infected K562 (**c**) and infected K562 (**d**) cells at 5 dpi. (**e**) Column chart showing the population distribution of THP1 and K562 cells across different quadrants, with or without PRV7S infection. (**f**–**i**) Cell cycle analysis of non-infected THP1 (**f**), infected THP1 (**g**), non-infected K562 (**h**) and infected K562 (**i**) cells at 5 dpi. (**j**) Column chart showing the cell cycle phases of THP1 and K562 cells with or without PRV7S infection. (**e**,**j**) Data are presented as mean ± SD. Three independent experiments were performed, each with three replicates. A two-way ANOVA was conducted. *p < 0.05; ****p < 0.0001.

Flow cytometry analysis was conducted on PRV7S-infected THP1 and K562 cells to detect early and late apoptosis, as well as necrosis. A fluorescein isothiocyanate (FITC) conjugated Annexin V antibody-PI coupled staining method was used to differentiate cell death events^19,23^. The analysis revealed variations in cell death occurrences among the leukaemia cell lines. Specifically, flow cytometry scatter plots showed higher population intensities in the late apoptotic and necrotic quadrants for infected THP1 cells, while infected K562 cells exhibited increased population across the necrotic, early, and late apoptotic quadrants (Fig. 3a–d). Statistical analysis indicated a significant increase in necrotic THP1 cells and apoptotic K562 cells compared to uninfected controls (Fig. 3e). Both leukaemia cell lines also demonstrated a statistically significant decrease in viable cell populations relative to the negative control (Fig. 3e).

Cell cycle analysis confirmed that PRV7S infection did not induce statistically significant cell cycle arrest in either THP1 or K562 cells (Fig. 3f–j). This absence of cell cycle inhibition may be therapeutically advantageous by potentially avoiding delayed apoptosis in cancer cells^24^.

### PRV7S triggered caspase-mediated apoptosis

**Fig. 4:**
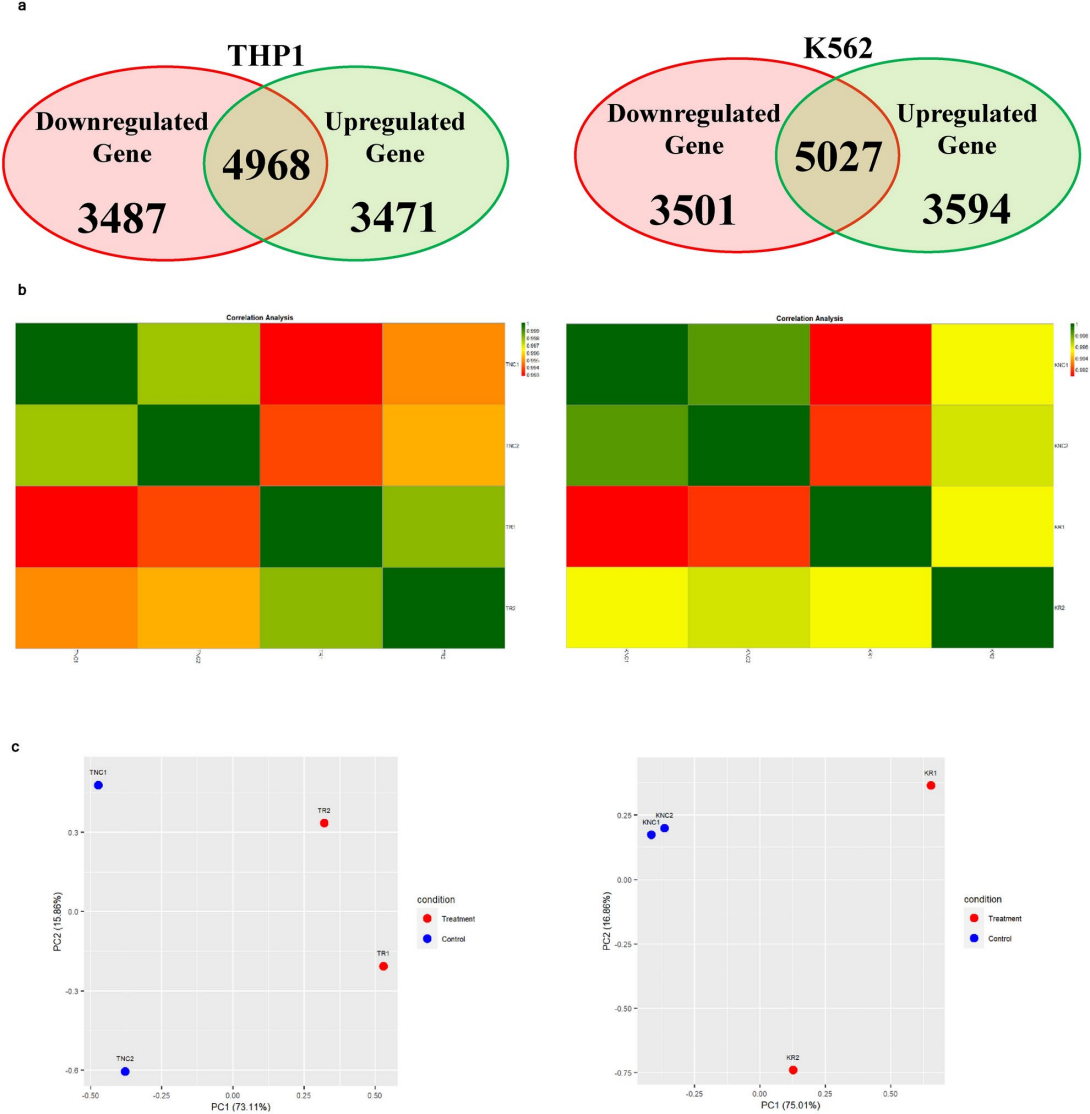

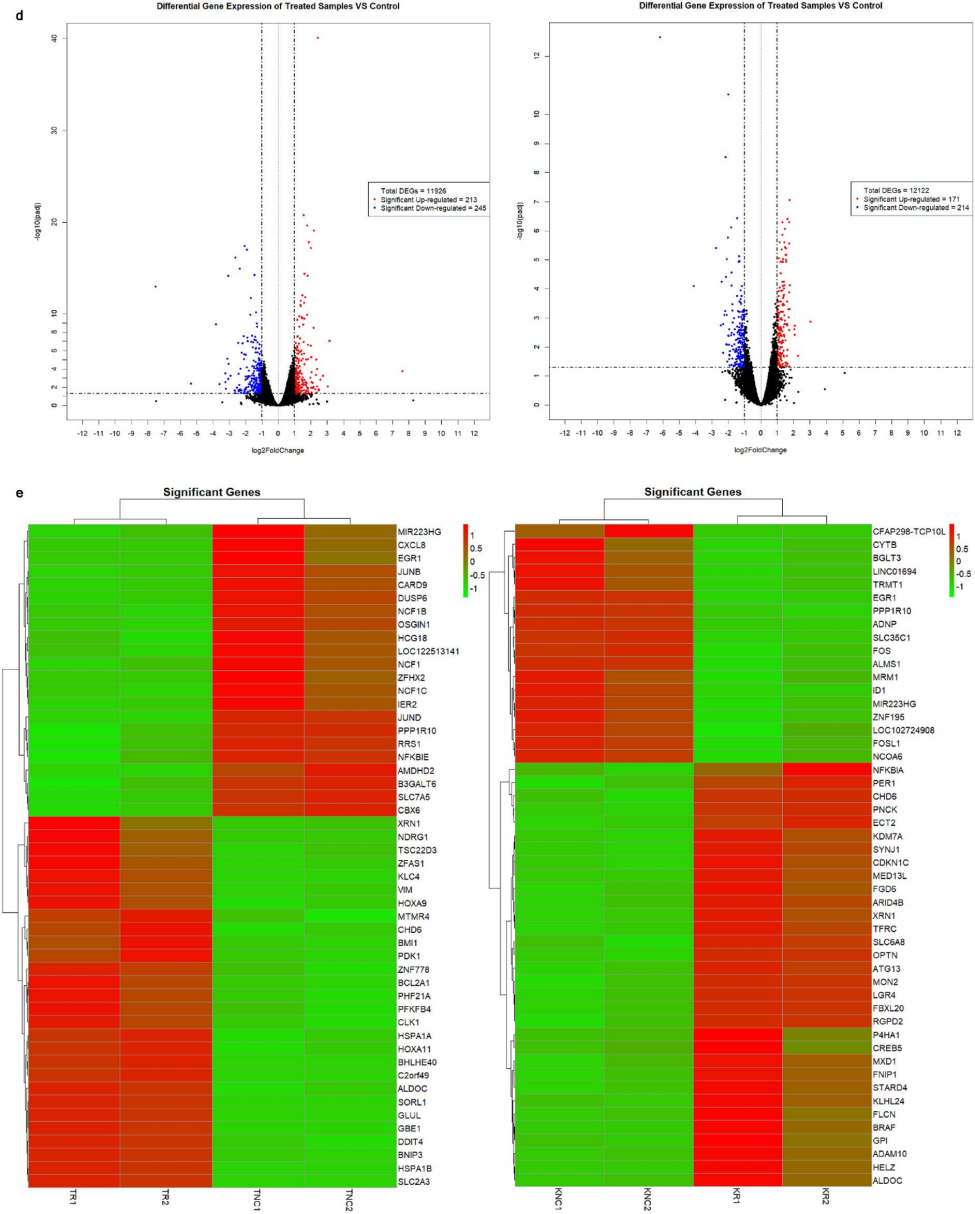

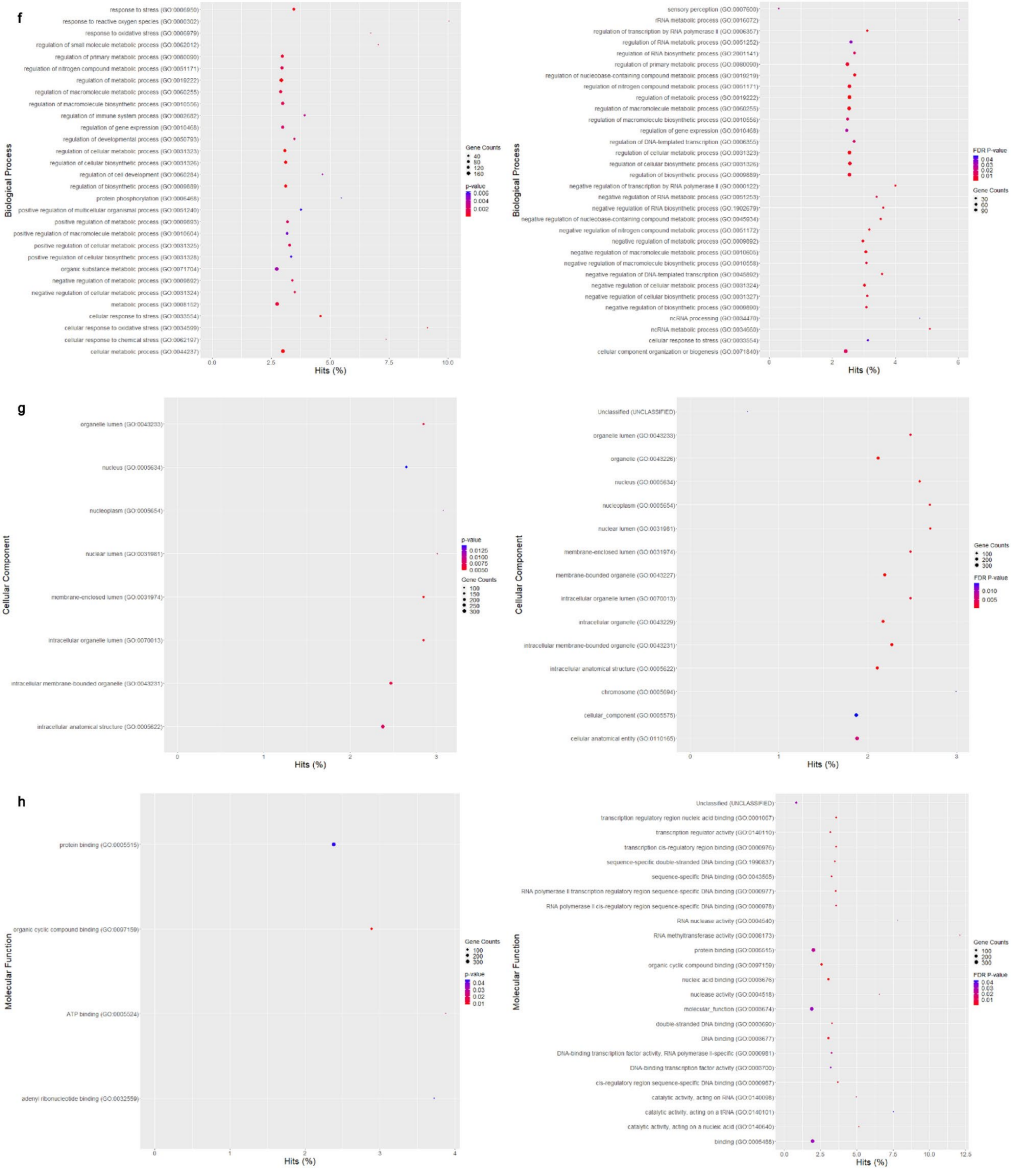

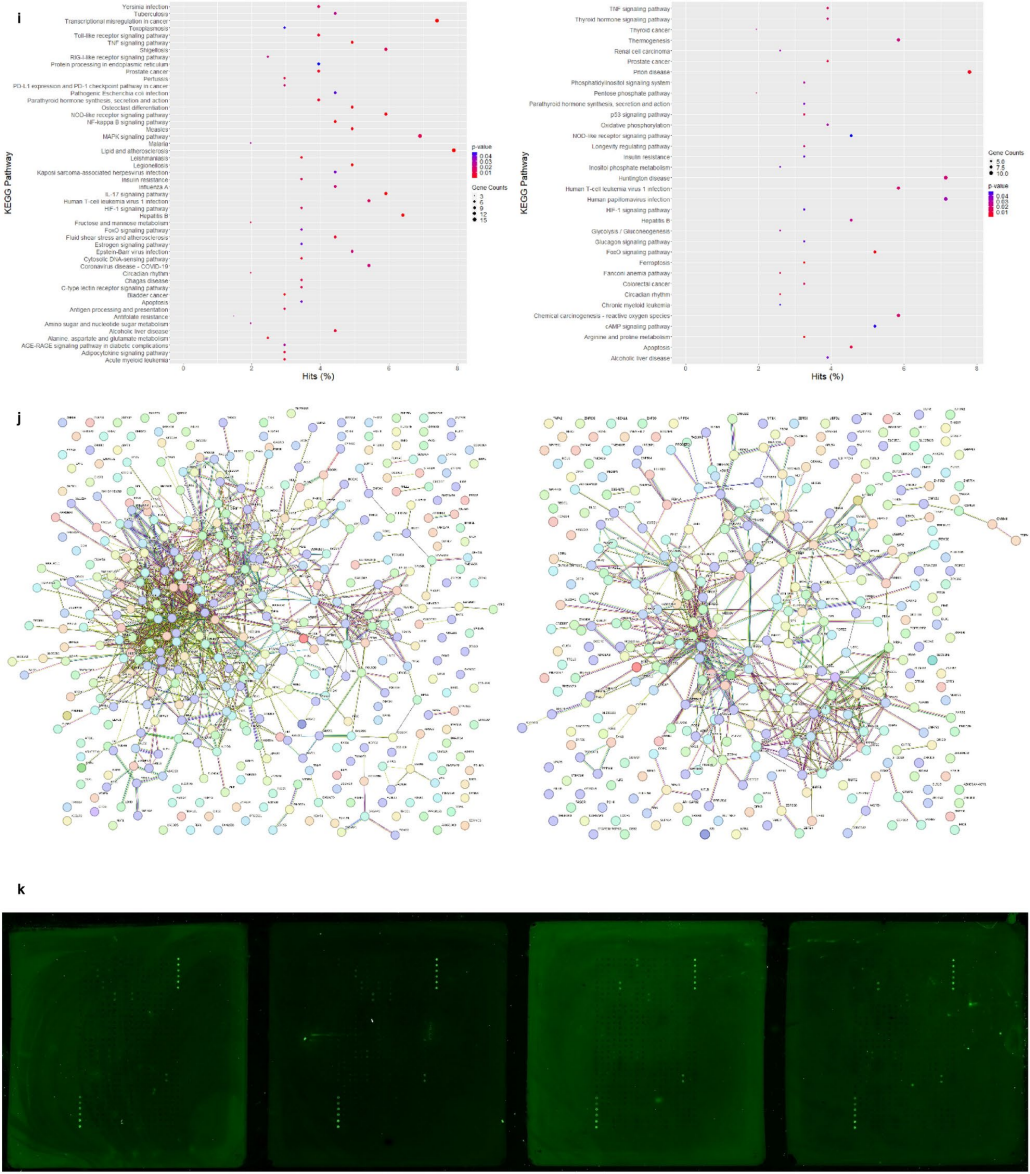

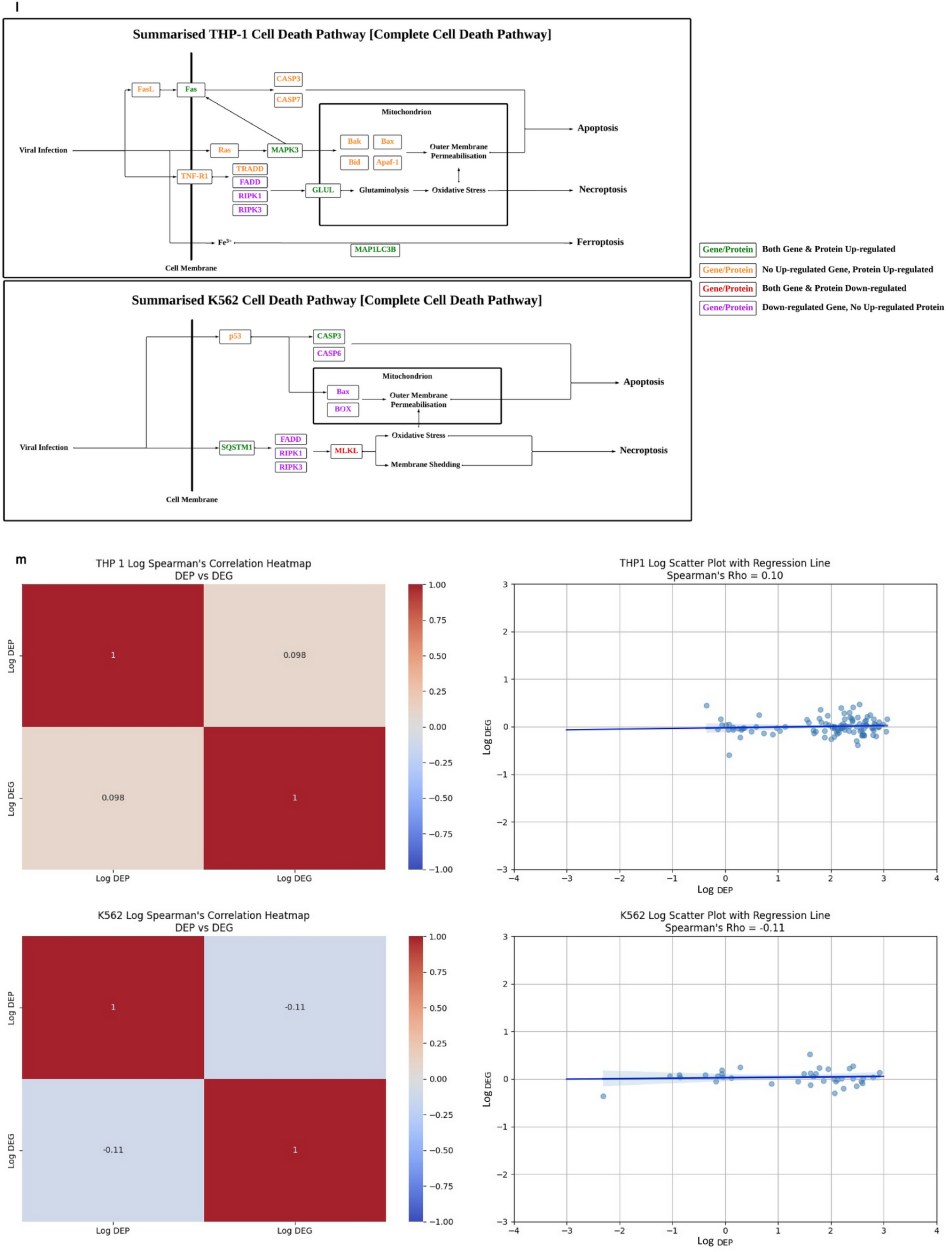

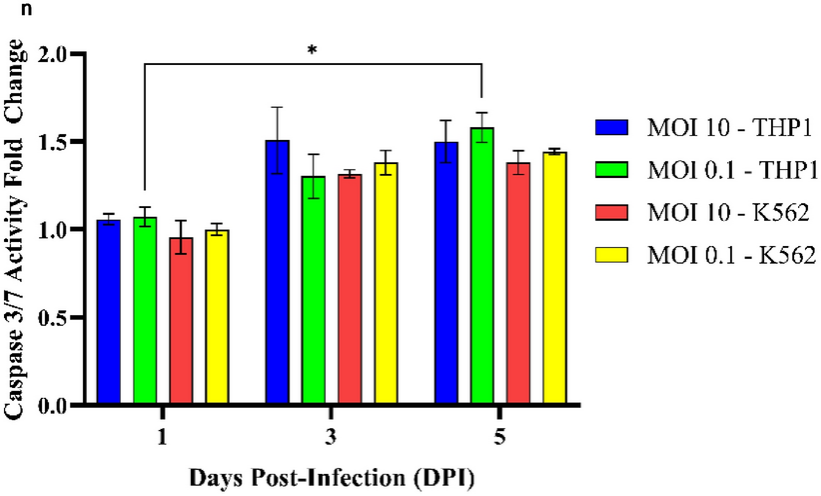
PRV7S triggered caspase-mediated apoptosis. (**a**) Venn diagram of differentially expressed genes (DEGs). The total number of DEGs identified in PRV7S-infected THP-1 (left) and K562 (right) cells was 11,926 and 12,122, respectively. In THP-1 cells (left), 3471 genes were significantly upregulated (≥ 1.2 fold change), while 3487 genes were significantly downregulated (≤ − 1.2 fold change). In K562 cells (right), 3594 genes were significantly upregulated, and 3501 were significantly downregulated. (**b**,**c**) Correlation analysis of THP-1 and K562 cells. (**b**) Correlation heatmap of THP-1 (left) and K562 (right) cells. (**c**) Principal component analysis (PCA) plot of THP-1 (left) and K562 (right) cells. (**d**,**e**) Differential gene expression analysis of THP-1 and K562 cells. (**d**) Volcano plot of THP-1 (left) and K562 (right) cells. (**e**) Hierarchical clustering heatmap of DEGs in THP-1 (left) and K562 (right) cells. (**f**,**g**) Functional enrichment analysis of THP-1 and K562 cells. (**f**–**h**) Gene Ontology (GO) analysis was performed using significant DEGs from THP-1 and K562 cells, with scatter plots generated for biological processes (**f**), cellular components (**g**), and molecular functions (**h**) for both cell lines. (**i**) Pathway enrichment analysis based on the Kyoto Encyclopedia of Genes and Genomes (KEGG) for THP-1 (left) and K562 (right) cells. (**j**) Gene–gene interaction network derived from the Search Tool for the Retrieval of Interacting Genes/Proteins (STRING) for THP-1 (left) and K562 (right) cells. (**a**–**j**) Results from THP-1 are consistently shown on the left, and those from K562 on the right. (**k**) Cell death biomarkers antibody array of THP1 (first quadrant from the left is negative control; second quadrant from the left is PRV7S-infected) and K562 (third quadrant from the left is negative control; fourth quadrant from the left is PRV7S-infected) was performed using a glass slide with duplicates. (**l**) The summarised cell death signalling pathways of THP1 (top) and K562 (bottom) cells based on the complete mechanism pathway (Supplementary Figs. 2, 3). (**m**) Spearman’s correlation analysis. Spearman’s correlation heatmaps (Top and bottom left) and scatter plot (top and bottom right) indicate no significant (THP1’s *p* = 0.3125, K562’s *p* = 0.5051) relationship between DEP and DEG. (**n**) Caspase 3/7 activity fold change in THP-1 (blue and green) and K562 (red and yellow) cells infected with PRV7S at different MOIs. Data are presented as mean ± SD from three independent experiments, each performed with three replicates. Statistical analysis was performed using two-way ANOVA. *p < 0.05.

Differentially expressed genes (DEGs) and proteins (DEPs) were identified in PRV7S-infected THP1 and K562 cells compared with non-infected cells, using mRNA sequencing and a human cell death biomarker antibody microarray, respectively. Transcriptomic profiling and analysis revealed a higher number of DEGs in K562 cells than in THP1 cells (Fig. 4a). A correlation heatmap (Fig. 4b) and principal component analysis (PCA) plot (Fig. 4c) showed strong correlation within groups (e.g., treatment vs. treatment, control vs. control) but weaker correlation between different groups (treatment vs. control). A total of 458 (THP1) and 385 (K562) significantly upregulated and downregulated genes met the filtering criteria of adjusted p-value < 0.05 and fold change > 2 or < − 2 (Fig. 4d). The top 50 significant DEGs for each cell line were clustered in a heatmap based on Euclidean distance (Fig. 4e).

Gene Ontology (GO) analysis of significant DEGs was conducted via the GO web browser (PANTHER Overrepresentation Test). Fisher’s Exact test with False Discovery Rate (FDR) correction was used for statistical validation, and only GO terms with FDR p-values < 0.05 were reported and presented in dot plots by category (Fig. 4f–h). Pathway enrichment analysis, based on the Kyoto Encyclopaedia of Genes and Genomes (KEGG), was then performed to identify pathways associated with significant DEGs (Fig. 4i). The DEGs in both cell lines, with higher gene hits, were primarily linked to pathways involved in malignancy-related genetic dysregulation, inflammatory responses, endocrine and metabolic homeostasis, and, critically, regulation of cell death (Fig. 4i). A gene–gene interaction network was constructed using the Search Tool for the Retrieval of Interacting Genes/Proteins (STRING), representing predicted associations among significant DEGs (Fig. 4j).

For the human cell death biomarker antibody microarray, fluorescence intensities were detected on a pre-coated glass slide using a microarray scanner (Fig. 4k). This assay, focused on DEPs relevant to cell death, helped elucidate PRV7S-induced cell death pathways, including the apoptotic pathway, cyclic adenosine monophosphate (cAMP) signalling pathway, tumour necrosis factor (TNF) signalling pathway, nuclear factor kappa B (NF-κB) signalling pathway, toll-like receptor signalling pathway, mitogen-activated protein kinase (MAPK) signalling pathway, phosphatidylinositol-3-kinase/protein kinase B (PI3K/AKT) pathway, mammalian target of rapamycin (mTOR) signalling pathway, Janus kinase-signal transducer and activator of transcription (JAK-STAT) pathway, adipocytokine signalling pathway, and p53 signalling pathway (Fig. 4l, Supplementary Figs. 2, 3). However, no significant or strong correlation was observed between DEGs and DEPs (Fig. 4m). The discordant of DEGs and DEPs has also been reported in numerous other studies^25–34^.

In line with PRV7S-induced cell death mechanisms previously reported in nasopharyngeal carcinoma cells, infected THP1 and K562 cells showed involvement of pro-apoptotic caspases, including caspase-3 and caspase-7, leading to apoptosis (Fig. 4l)^19^. Elevated caspase-3/7 activity was further confirmed via a caspase-3/7 activity assay (Fig. 4n). Interestingly, expression of the *CASP-3* and *CASP-7* genes was not consistent with levels of their respective caspase proteins (Fig. 4l), potentially due to complex apoptotic regulatory mechanisms, such as a negative feedback loop resulting in inverse expression between genes and proteins at specific time points^35^. Remarkably, PRV7S-induced cell death in THP1 and K562 cells may not be restricted to apoptosis, as necroptosis, pyroptosis, ferroptosis, and autophagy pathways could also be involved (Fig. 4l, Supplementary Figs. 2, 3).

### PRV7S can infect and reproduce in human cancer stem cell

**Fig. 5:**
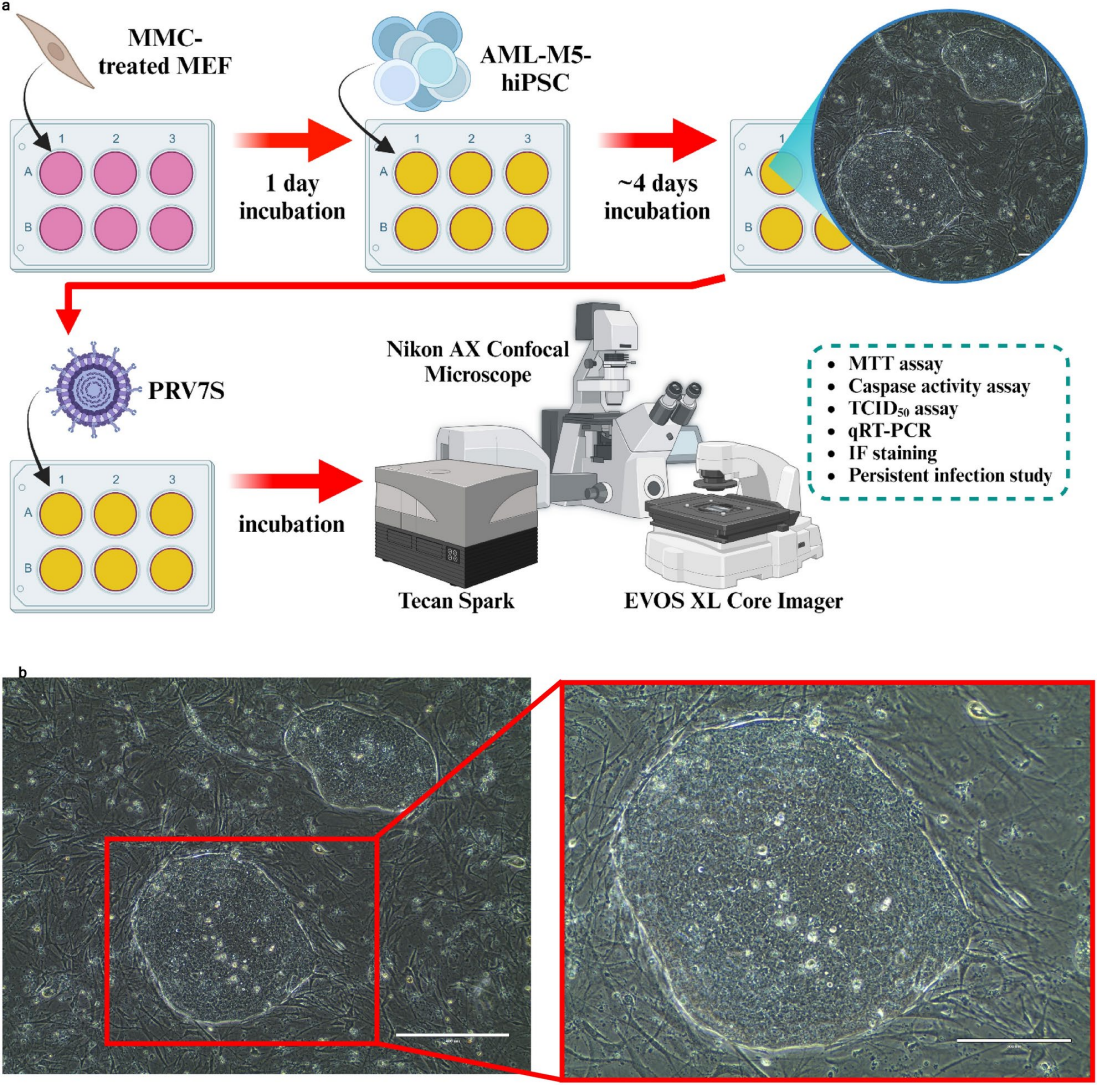

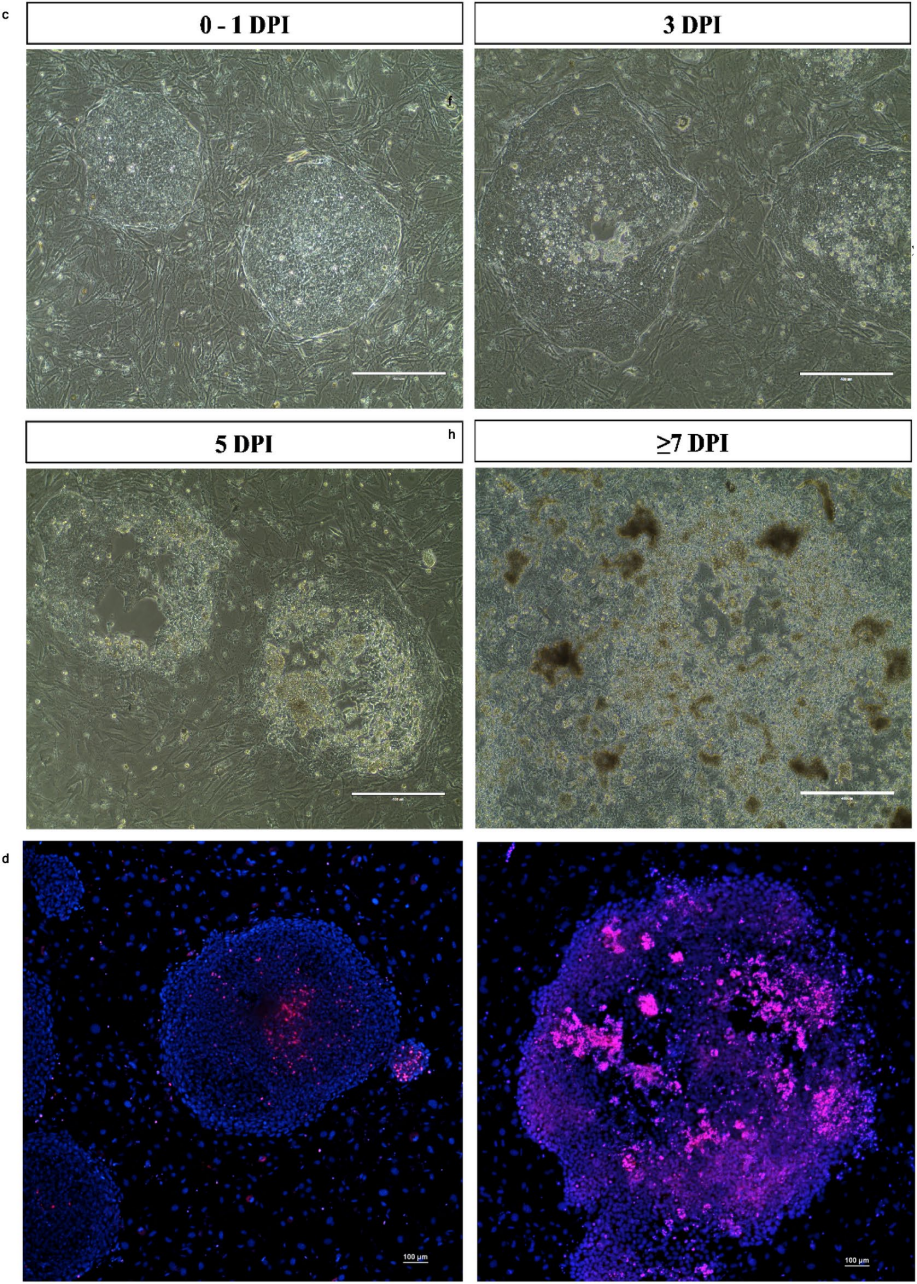

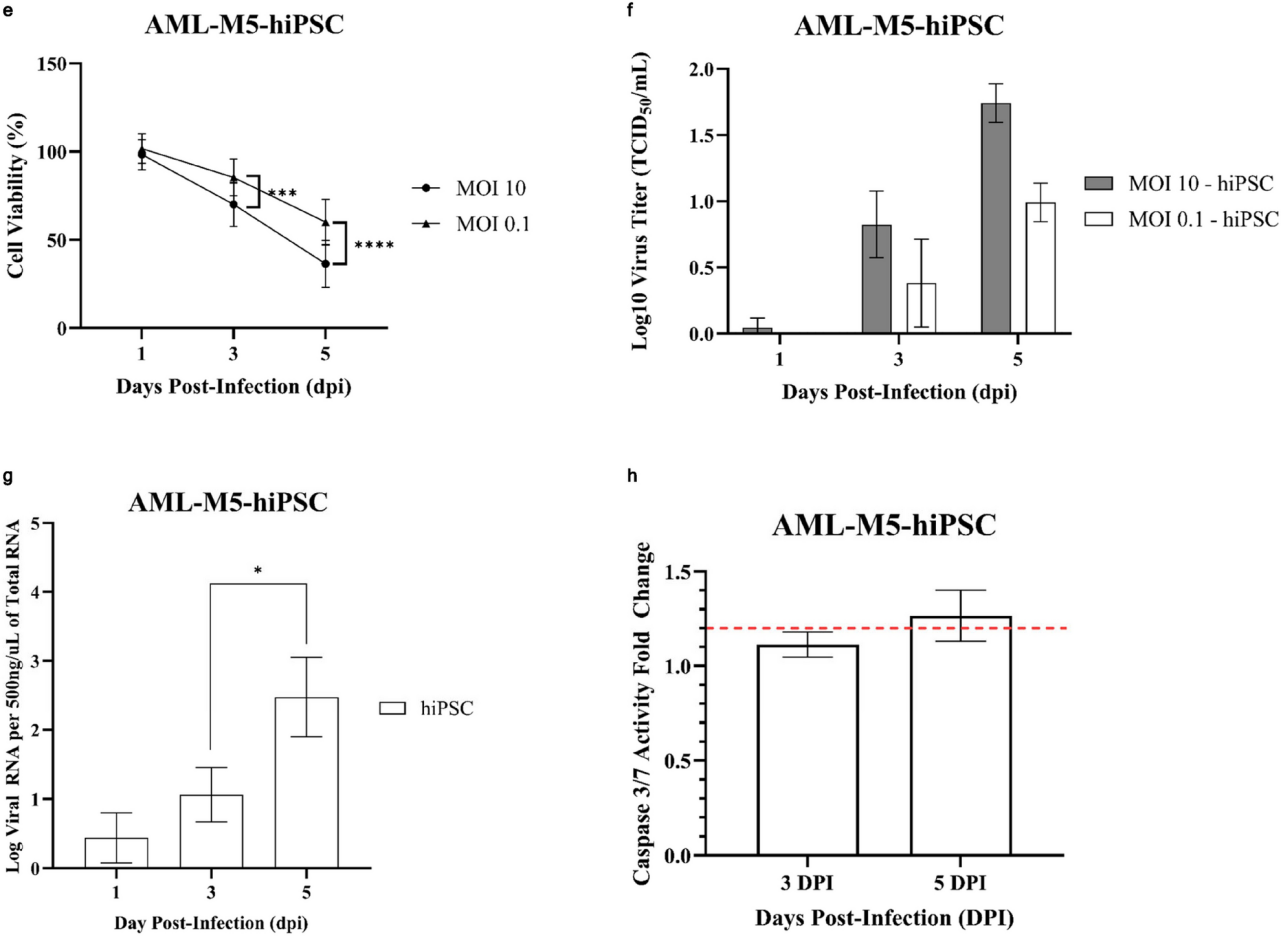
PRV7S can infect and reproduce in human cancer stem cell. (**a**) Schematic illustration of experiments performed on AML-M5 human induced pluripotent stem cell (hiPSC), also known as human cancer stem cell. AML-M5 hiPSC were seeded into multi-well plates and subsequently infected by PRV7S. Cell viability, caspase activity, viral titre and viral RNA from infected cells were measured over time. Created with BioRender.com (https://BioRender.com/r23t298). (**b**) Micrographs of non-infected, healthy colonies of AML-M5 hiPSCs seeded on mitomycin C-treated primary mouse embryonic fibroblasts (MEFs). Scale bars: 400 μm (left), 200 μm (right). (**c**) hiPSCs infected with PRV7S at a multiplicity of infection (MOI) of 0.1 over 7 days. Pronounced cytopathic effects (CPE) and plaques (clear zones) were observed from 3 days post-infection (dpi) onwards. Scale bars: 400 μm. (**d**) Hoechst 33342 and propidium iodide (PI) staining was performed on negative control hiPSC colonies (left) and PRV7S-infected colonies (right). The PRV7S-infected colony exhibited more intense PI staining compared to the negative control. Scale bars: 100 μm (left and right). (**e**) Cell viability of PRV7S-infected hiPSCs was assessed using the MTT assay over 5 days. A statistically significant decrease in cell viability was observed from 3 dpi onwards. (**f**,**g**) Viral kinetics of PRV7S propagation in hiPSCs were determined using the TCID50 assay (**f**) and qPCR (**g**). Viral titre and viral RNA increased from 1 to 5 dpi. (**h**) Caspase-3/7 activity in PRV7S-infected hiPSCs increased slightly by more than 1.2-fold at 5 dpi. The red dashed line indicates a 1.2-fold change threshold. (**e**–**h**) Data are presented as mean ± SD. Three independent experiments were performed, each with four replicates. ANOVA or *t*-test were conducted. *p < 0.05; ***p < 0.001; ****p < 0.0001.

Similar to the AML and CML cell lines, AML-M5 human induced pluripotent stem cells (hiPSCs) were subjected to various assays upon PRV7S infection at a multiplicity of infection (MOI) of 0.1 to evaluate cell susceptibility and viral replication capacity (Fig. 5a–d). The hiPSCs, previously reprogrammed by Chiew et al. (2017), require culturing on Mitomycin C-treated mouse embryonic fibroblast (MEF) feeder cells^17^. Healthy hiPSC colonies appear undifferentiated and compact, with clear, well-defined colony borders and no marginal spikes (Fig. 5b). However, PRV7S-infected hiPSCs developed progressive plaque zones of cell lysis over time even at a low viral concentration (Fig. 5c). Moreover, infected hiPSC demonstrated a higher intensity of PI staining than uninfected cells, which indicates significant cell death events (Fig. 5d). MTT assays demonstrated a dose-dependent decline in cell viability in infected hiPSCs (Fig. 5e). Notably, PRV7S was able to infect, replicate, and egress from hiPSCs, as evidenced by the detection of extracellular viral titres and intracellular viral RNA using TCID_50_ assay and qRT-PCR, respectively (Fig. 5f,g). The cell death mechanism in infected hiPSCs may resemble that observed in THP1 and K562 cells, as indicated by increased activity of executioner caspase-3 and caspase-7, suggesting apoptotic cell death (Fig. 5h).

### PRV7S does not persist and cause significant pathological damage to immunocompetent mice

**Fig. 6:**
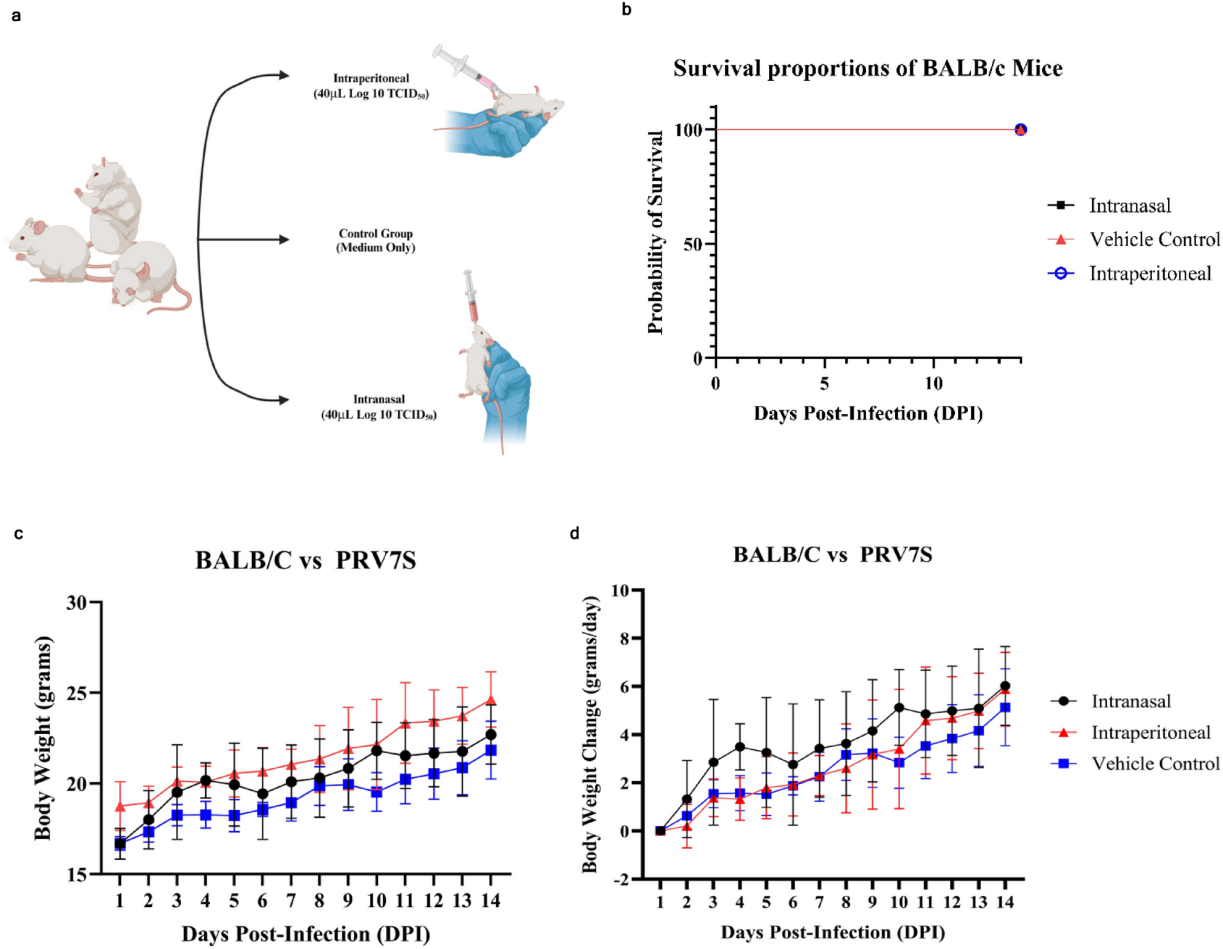

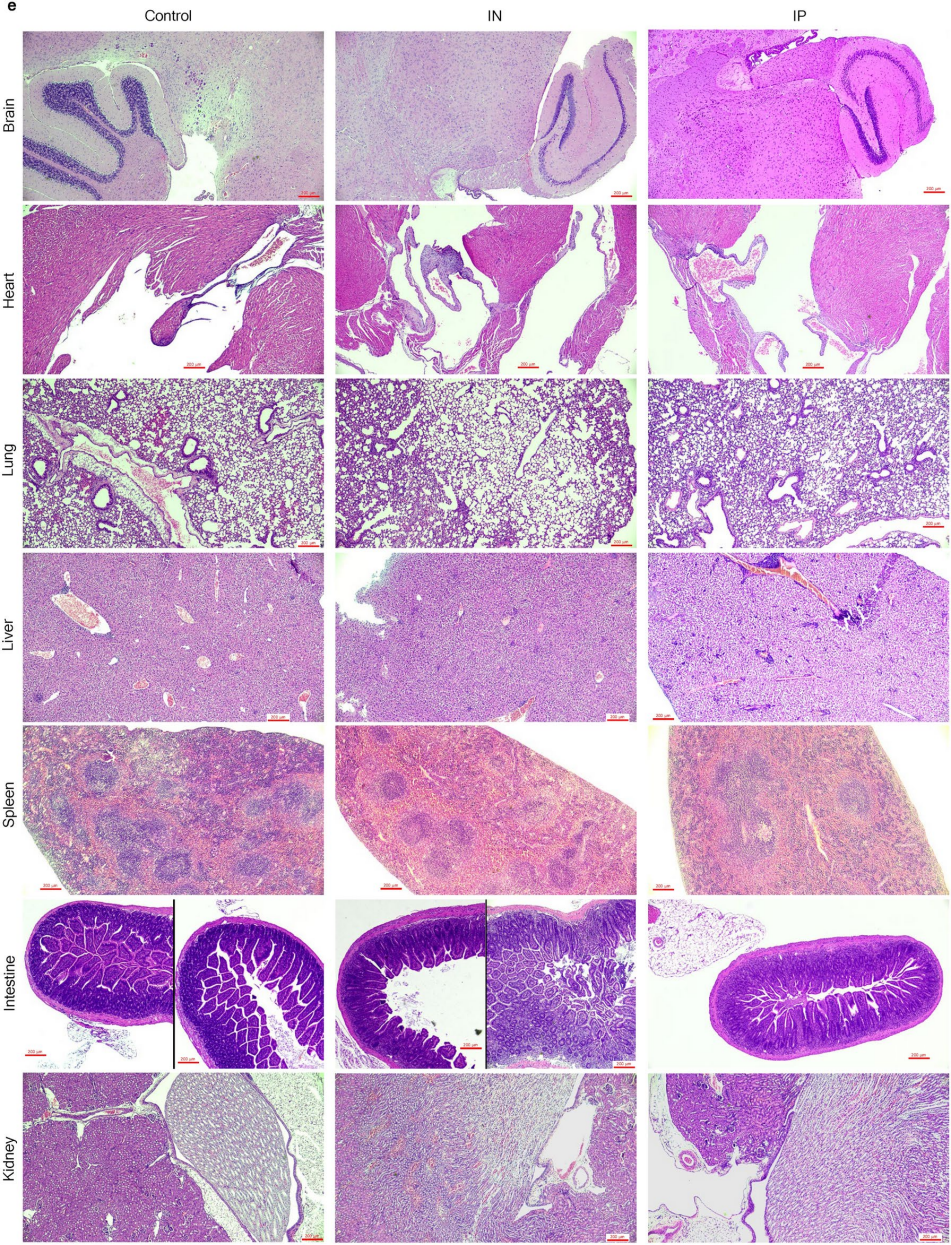

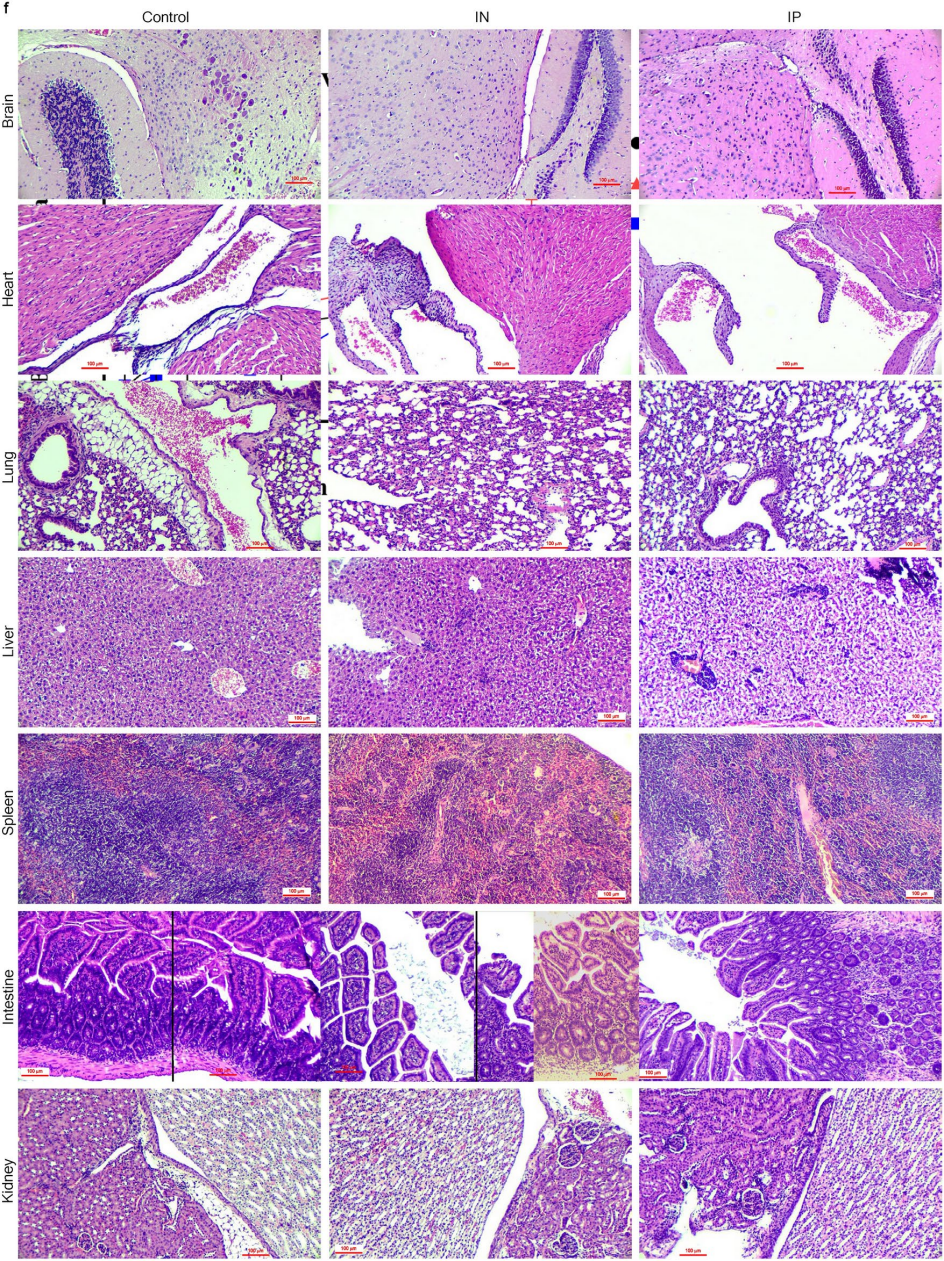

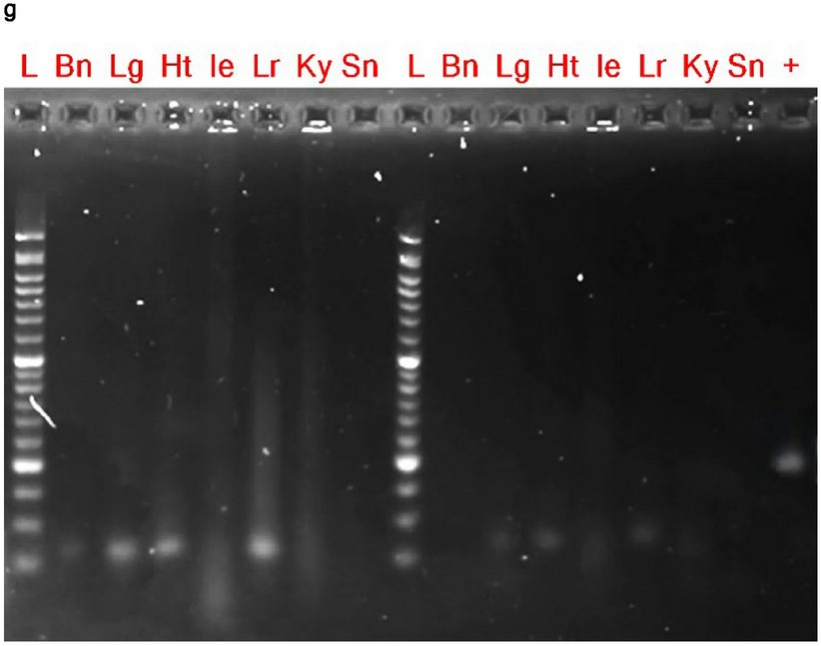
PRV7S does not persist and cause significant pathological damage to immunocompetent mice. (**a**) Schematic illustration of Log 9 TCID_50_/mL PRV7S inoculation into BALB/c mice via different route of administration. Created with BioRender.com (https://BioRender.com/w24s510). (**b**) No death (100% survival rate) observed in PRV7S-infected BALB/c mice after 14 days post-infection (dpi). (**c**,**d**) PRV7S-infected BALB/c mice have an overall increasing trend of body weight and body weight changes over 14 dpi. No significant weight loss was observed. (**e**,**f**) Histology from PRV7S-infected BALB/c mice demonstrating no prominent pathological organ damage on all tested organs, compared with uninfected mice after 14 dpi. Scale bars: 200 μm (**e**), 100 μm (**f**). (**g**) PRV7S was detected negative in all organs at 14 dpi. *L* 50 base pairs (bp) DNA ladder, *Bn* brain, *Lg* lung, *Ht* heart, *Ie* intestine, *Lr* liver, *Ky* kidney, *Sn* spleen; + positive control. (**c**,**d**) Data are presented as mean ± SD. Three independent experiments were performed, each with three mice (n = 3) per group (total 3 groups).

An in vivo study was conducted to evaluate the safety profile of PRV7S when administered within a biological system. BALB/c mice were inoculated with PRV7S at a concentration of Log 9 TCID_50_/mL via intraperitoneal and intranasal routes, while the control group received only Dulbecco’s Modified Eagle Medium (DMEM) (Fig. 6a). Throughout the 14-day observation period, all groups maintained 100% survival, indicating no PRV7S-induced mortality (Fig. 6b). Furthermore, no significant body weight loss was observed in any group throughout the 14-day period, although a slight decrease was noted between 4 and 6 dpi in the intranasal group (Fig. 6c,d). Interestingly, histopathological analysis revealed no significant tissue damage in any of the tested organs (Fig. 6e,f). Moreover, no PRV7S viral RNA was detected in any organs across all groups, including the brain, heart, lung, liver, spleen, intestine, and kidney (Fig. 6g). The qRT-PCR results were negative for PRV7S in all organs, with CT values > 35 (data not shown). Collectively, these findings suggest that PRV7S exhibits an acceptable safety profile in immunocompetent mice, with no evidence of persistent infection in either in vitro or in vivo settings.

## Discussion

Our study confirms that PRV7S is capable of infecting, replicating within, and exerting oncolytic activity on AML and CML cell lines, as well as on AML-M5-hiPSC. We employed transcriptomic analysis and human cell death biomarker antibody microarray to determine the cell death mechanism in PRV7S-infected cells. Albeit apoptosis is the focused pathway in PRV7S-induced cell death, the analysis of the complete cell death pathway (Supplementary Figs. 2, 3) yields a complex, interconnected network of DEGs and DEPs, suggesting the involvement of additional pathophysiological and biochemical subtypes of cell death. These results offer critical insight into the roles and interaction between significant DEGs and DEPs in regulating cell death following PRV7S infection in myelogenous liquid tumours, with potential relevance to solid tumours^19^. Furthermore, the PRV7S-induced oncolytic effect on human cancer stem cells may hold promise in preventing relapse in AML or CML and overcoming chemotherapeutic resistance^36^.

Beyond demonstrating the oncolytic potential of PRV7S in vitro, we included an in vivo evaluation to assess the safety of PRV7S in an internal environment of mice. Clinically, the virus was known to cause respiratory and gastrointestinal infections^18^. While some pteropine orthoreoviruses have been reported to cause fatal infections in mice^37–40^, we uncovered that the direct administration of the PRV7S, either intranasally or intraperitoneally, was generally well-tolerated, with no histopathological complications observed in the treated mice, particularly in respiratory and intestinal tissues. Furthermore, no persistent infection was detected in the treated groups, demonstrating a favourable safety profile for PRV7S in vivo.

However, there are certain limitations in the transcriptomic study. First, the comparison of mRNA profiles between uninfected and PRV7S-infected groups was conducted at a single time point, specifically at 5 dpi. As shown in cell viability assays, flow cytometry analysis, and studies on persistent infection, cell viability was reduced in both infected cell lines compared to controls, which maintained a higher viable cell count. This may have led to higher mRNA concentrations for certain genes in the control group and subsequently lower mRNA levels in infected cells. Second, regulatory mechanisms influence the differential expression of specific genes and proteins. For instance, previous studies have shown that direct oncolysis induced by OVs such as adenovirus, ARV, and MRV is associated with P53-mediated apoptosis and autophagy^41–45^, aligning with our findings. We observed significant upregulation of P53 protein in THP1 (76.88 folds) and K562 cells (113.59 folds), despite *TP53* gene downregulation in THP1 cells (1.7-fold) and no differential gene expression (< 1.2-fold change) in K562 cells. This discrepancy may reflect an initial upregulation of the *TP53* gene and overexpression of the P53 protein at 5 dpi, followed by a negative feedback loop that suppresses *TP53* gene expression. Supporting this, genes associated with *TP53* downregulation, including *CCNG1* (1.5 folds) and *PPM1J* (1.4 folds) in THP1 cells, and *CCNG1* (1.8 folds), *SIAH-1* (1.4 folds) and *PPM1D* (1.6 folds) in K562 cells, were upregulated^46–48^. Furthermore, MDMX (MDM4) may be dephosphorylated and stabilised by the PPM1D and PPM1J. The upregulated MDM4 in THP1 (1.4 folds) and K562 (1.4 folds) cells, along with the increased expression of protein phosphatase 1 isoforms, potentially affecting *TP53* gene transcription^48^. Beyond the negative feedback loop, mRNA and protein stability may also play a critical role in modulating RNA and protein levels^43,48,49^. Besides, microRNA (miRNA) is also a key contributor to mRNA degradation and translational repression^50^. Interestingly, OV infection may modulate miRNA expression. In some cases, alterations in miRNA could even enhance oncolytic effects^51,52^. Moreover, transcription factors, such as AP-1 and P53 serve as important regulators of gene expression^53–56^. For example, the *c-Jun/Jun* gene was upregulated (1.4 folds) in PRV7S-infected K562 cells and it may suppress the transcription of the *TP53* gene^55^. However, the AP-1 signalling pathway is very complex, as it can act in both pro-apoptotic and oncogenic capacities^55,57^.

Furthermore, the in vivo study should include multiple animal models (e.g., *Mesocricetus auratus*) and compare the effects of different dosages. Besides, we did not include an in vivo assessment of the oncolytic effects of PRV7S in mice with induced AML or CML. Such experiments could provide a deeper understanding of the PRV7S oncolytic effect on leukaemia cells within a complex biological environment, where external factors such as cell–cell interactions and exosome-mediated signalling may significantly impact therapeutic outcomes^58–61^. Thus, future studies are encouraged to explore the complex regulatory mechanisms underlying differential gene and protein expression in PRV7S-infected cancer cells, the impact of the host immune response on the oncolytic activity of PRV7S, and the efficacy and potency of PRV7S as an OV in cancer-bearing mice before progress into clinical trials. Lastly, additional studies on the proteins involved in immunoregulation and immunomodulation in relation to oncolytic vaccines should also be considered.

## Methods

### Cell viability assays

#### Cancer cells and virus culture maintenance

Vero (ATCC CCL81), THP-1 (ATCC TIB-202), and K562 (ATCC CCL-243) cells were cultured in Dulbecco’s Modified Eagle Medium (DMEM), Roswell Park Memorial Institute (RPMI) 1640 Medium, and Iscove’s Modified Dulbecco’s Medium (IMDM), respectively. All media were sourced from Nacalai Tesque Inc., Japan, and supplemented with 5% foetal bovine serum (FBS; Biosera, France) and 1% penicillin–streptomycin–amphotericin B antibiotics (Abcam, UK). Cell cultures were maintained in a humidified 5% CO_2_ incubator at 37 °C. Routine mycoplasma testing was performed, particularly if abnormalities were observed in the cultures (e.g., rapid pH or colour changes), using a mycoplasma PCR detection kit (SouthernBiotech, USA), with results confirmed by a mycoplasma staining detection kit (Solarbio, China). Vero, THP-1, and K562 cells were treated routinely with a mycoplasma prevention reagent (Sangon Biotech, China) and a mycoplasma removal reagent (Beyotime, China), respectively. PRV7S, the virus used in this study, was originally isolated from a patient by Chua et al. (2011)^62^. Vero cells were used to propagate PRV7S. In brief, 100 μL of PRV7S stock was added to Vero cultures at approximately 80% confluency in T75 flasks, followed by incubation until complete cell lysis was observed. The supernatant was subsequently harvested for TCID_50_ determination.

#### MTT assay

The 3-(4,5-dimethylthiazol-2-yl)-2,5-diphenyltetrazolium bromide (MTT) assay was conducted as previously described to assess the viability of various cell lines infected with PRV7S at MOI of 0.1 and 10 over 1, 3, and 5 dpi^19^. The assay was performed in 96-well plates, with each cell line seeded at a density of 1 × 10^4^ cells per well. Each experiment included four replicates per condition, and at least five independent experiments were conducted. Formazan absorbance was measured at 570 nm and 630 nm using the BioTek Epoch Microplate Spectrophotometer (Agilent, USA).

#### Validation assays

Cell viability was further validated using PI staining and trypan blue exclusion assays^63–65^. Briefly, 25 μL of 50 μg/mL Invitrogen™ Propidium Iodide (Thermo Fisher Scientific, USA) was added to each well of a black 96-well plate (Thermo Fisher Scientific, USA) and incubated for 10 min at room temperature (RT) in the dark. Fluorescence intensity was measured at 530 nm excitation (12 nm bandwidth) with a 620 nm emission filter (12 nm bandwidth) using a Varioskan™ LUX multimode microplate reader (Thermo Fisher Scientific, USA). The plate was then stored at − 20 °C and thawed at RT for a second reading after 24 h. For the trypan blue assay, Gibco™ 0.4% Trypan Blue Solution (Thermo Fisher Scientific, USA) was added at a 1:1 ratio to each well of a 96-well plate. A 10 μL aliquot of this mixture was transferred to a gridded, improved Neubauer-ruled hemocytometer, with two independent counts performed for each replicate using a Nikon TS100 inverted microscope (Nikon Instruments, USA). Additionally, an Invitrogen Countess 3 Automated Cell Counter (Thermo Fisher Scientific, USA) was first optimised and utilised to verify the cell count.

### Study of persistent infection and virus kinetics

#### Microscopy evaluation

Generally, microscopic evaluations were conducted as previously described to observe the presence of CPE or notable morphological changes in PRV7S-infected cells on different time points^19^, using a ZEISS Axio Observer inverted microscope (ZEISS, Germany) and EVOS™ XL Core Imaging System (Thermo Fisher Scientific, USA). The observation time points varied between experiments. Cell seeding density depended on the type of culture container used (e.g., T25 flask, 6-well plate, etc.).

#### Attempt to establish persistent infection

Following a similar approach to Loh et al. (2023), a long-term study was conducted to assess the capacity of PRV7S to establish persistent infection in THP-1 and K562 cells^22^. Briefly, approximately 3 × 10^5^ THP-1 and K562 cells were infected with PRV7S at a MOI of 0.1 in 6-well plates and incubated under standard culture conditions. The culture medium was refreshed on days 5 and 11 dpi. Thereafter, it could be refreshed at intervals of at least five days. Microscopic evaluation and sample collection were performed at 1, 3, 5, 7, 9, 11, 14, 21, 28, 35, and 42 dpi for K562 cells and up to 21 dpi for THP-1 cells. Supernatants were collected for TCID_50_ assays to quantify viral release, while cell pellets were harvested for qPCR-based viral RNA quantification.

#### Median tissue culture infectious dose (TCID_50_) assay

As previously described, virus titres were determined using the TCID_50_ assay, an endpoint dilution method conducted on Vero cells^66^. The TCID_50_ assay protocol was adapted with minor modifications from previous studies^67,68^. Unlike Mok et al. (2015), the supernatant alone was used here to measure extracellular virus titres, rather than a combination of supernatant and whole lysate^67^. The mean of six readings for each dilution was used to calculate virus titres according to the Reed–Muench method^69^. The obtained TCID_50_ values were used to calculate the MOI, commonly defined as the ratio of infectious viral particles to the number of target cells^70,71^. The following formulas were applied for MOI calculation:

1. Plaque forming units (pfu)/mL = 0.5 (TCID_50_ value)
2. MOI = $$\frac{pfu\; of\; a \;given \;virus \;species}{{Total \;number \;of\; cells}}$$

#### Real-time PCR viral RNA quantification

Harvested cell pellets were used for a three-step qRT-PCR (including melt curve analysis) to quantify viral RNA, performed on a CFX Connect Real-Time PCR Detection System (Bio-Rad, USA). Cell pellets underwent at least one freeze–thaw cycle to lyse cell membranes. Viral RNA was then extracted using the Viral Nucleic Acid Extraction Kit II (Geneaid Biotech, Taiwan) according to the manufacturer’s protocol. Equal concentrations of total RNA (1 μg) were used to synthesise complementary DNA (cDNA) with the QuantiTect Reverse Transcription Kit (Qiagen, Germany), following the manufacturer’s instructions. The qRT-PCR was carried out using the QuantiNova SYBR Green RT-PCR Kit (Qiagen, Germany) with primers PRVMiyazakiS4F2 (5′-CAACTTCCACTCGTTCGTTG-3′) and PRVMiyazakiS4R2 (5′-GATGATGTGGAAACGGATAC-3′) to amplify the S4 segment of PRV7S^72^. The thermal cycling conditions were: initial denaturation at 95 °C for 3 min, followed by 40 cycles of 95 °C for 10 s, 57 °C for 30 s, and 72 °C for 50 s. Three independent experiments were performed, each in triplicate.

### Cell death mechanism determination

#### Annexin V/PI and cell cycle assays using flow cytometry

THP-1 and K562 cells were seeded in T75 flasks at a density of 1.0 × 10^6^ cells per flask and infected with PRV7S at a MOI of 0.1. Unlike commonly used T25 flasks, T75 flasks were selected due to the longer incubation time (5 dpi) required for cell harvest. At 5 dpi, cells were resuspended and counted using the Countess 3 automated cell counter. Apoptosis was assessed using the BD Pharmingen™ FITC Annexin V Apoptosis Detection Kit I (BD Biosciences, USA), following the manufacturer’s protocol, as described in similar studies^73–75^. Briefly, cells were collected, washed twice with cold 1× phosphate-buffered saline (PBS), and resuspended in 1× Binding Buffer before being transferred into 5 mL FACS tubes at a density of at least 2.0 × 10^5^ cells per tube. FITC-Annexin V and PI staining was then performed on live cells (without fixation) by incubating them in the dark for 15–30 min at room temperature. Stained cells were analysed within an hour using the BD FACSCanto™ II Flow Cytometer with BD FACSDiva™ Version 6.1.3 software (BD Biosciences, USA).

For cell cycle analysis, the protocol was adapted from previous studies^73–77^. Briefly, harvested cells were washed with cold 1× PBS and fixed in cold 70% ethanol at 4 °C for at least 30 min, with optional overnight incubation. Following fixation, cells were washed with cold 1× PBS and centrifuged three times to thoroughly remove ethanol. Cells were then treated with 500 μL of 100 μg/mL (~ 7000 units/mL) RNase A (Qiagen, Germany) and incubated at 37 °C for at least 30 min (incubation at room temperature ≥ 15 °C is also effective). Subsequently, cells were incubated with 100 μg/mL PI (minimum 10 μg/mL) for an additional 30 min at room temperature in the dark before flow cytometric analysis. Cell cycle analysis was conducted using ModFit LT™ software (Verity Software House, USA), with guidance from technical support.

Flow cytometric analysis, including fluorescence compensation, was conducted together with a BD Biosciences Flow Cytometry Application Specialist. Three independent experiments were performed for both apoptosis and cell cycle analyses, each in triplicate.

#### Transcriptomic analysis (mRNA profiling)

THP-1 and K562 cells were seeded in T75 flasks at a density of 1.0 × 10^6^ cells per flask and infected with PRV7S at a MOI of 0.1. Cells were harvested at 5 dpi, centrifuged at 1000 rpm for 10 min to remove the supernatant, and subjected to at least one freeze–thaw cycle using a − 80 °C freezer. A total of 6 independent experiments were performed for each cell line and they were pooled into 2 samples per treatment group. Nucleic acid extraction was then performed with the Quick-DNA/RNA Miniprep Plus Kit (Zymo Research, USA), following the manufacturer’s protocol. Genomic DNA was removed with DNase I (Zymo Research, USA). After DNA degradation, RNA samples were assessed for purity and prepared using the Illumina Stranded mRNA Prep, Ligation Kit (Illumina, USA) according to the manufacturer’s instructions. Sequencing was conducted on the Illumina NovaSeq 6000 system (Illumina, USA) with paired-end 150 bp reads (PE150). Libraries were normalised to 0.5 nM, pooled, and loaded onto the NovaSeq platform. Demultiplexing was carried out using the Illumina DRAGEN Bio-IT Platform v3.9 (Illumina), which provides an RNA-seq (splicing-aware) aligner and RNA-specific analysis tools for gene expression quantification and gene fusion detection.

For differential gene expression (DEG) analysis, transcript quantification files were imported into R using tximport^78^, and DEGs were identified with DESeq2^79^. Functional enrichment analysis, which includes Gene Ontology and Kyoto Encyclopedia of Genes and Genomes (KEGG)^80–82^, was performed using clusterProfiler^83^, and gene–gene interaction networks were generated with STRING v12.0^84,85^. An overview of the Illumina DRAGEN RNA pipeline is provided in Extended Data Fig. 1a.

Accurate quantification and rigorous quality control of RNA samples and sequencing libraries are essential for a successful sequencing run. RNA concentrations were measured using a Qubit fluorometer (Thermo Fisher Scientific, USA). Quality assessment of sequencing data was performed with FastQC tools^86^, generating plots to evaluate data integrity. To translate raw read counts into meaningful gene expression measures, normalisation was performed to adjust for factors influencing read mappings, such as gene length^87^, GC content^88^, and sequencing depth^89^. Transcript abundance was quantified using Salmon, a fast and accurate tool for RNA-seq read quantification^90^. The reference transcriptome (FASTA format) and raw sequencing reads (FASTQ format) were used as inputs for read mapping and quantification. Sample and library quality check metrics were included in Extended Data Fig. 1b–e.

#### Human cell death biomarker antibody array

The Human Cell Death Biomarker Screening Array Kit (RayBiotech, USA) was used to detect 263 proteins involved in cell death pathways, following the manufacturer’s protocol with assistance from an application specialist. THP-1 and K562 cells were seeded in T75 flasks at a density of 1.0 × 10^6^ cells per flask and infected with PRV7S at a MOI of 0.1. Cells were harvested at 5 dpi, centrifuged at 1000 rpm for 10 min to remove the supernatant, and subjected to at least one freeze–thaw cycle at -80 °C. The cell pellet was then lysed in 1× Cell Lysis Buffer for 30 min on ice (or at 4 °C) and centrifuged at 13,600 rpm for 10 min at 4 °C. Protein concentration was measured in the clarified lysates using the Pierce™ BCA Protein Assay Kit (Thermo Fisher Scientific, USA). Equal concentrations of protein from each sample were biotinylated and purified with a spin column.

Biotinylated samples were incubated for 2 h at room temperature on a blocked glass slide pre-coated with capture antibodies. After several washes with 1× Wash Buffer, samples were incubated with 1× Cy3-Conjugated Streptavidin for 1 h at room temperature in the dark. The slide was then rinsed with 1× Wash Buffer and ddH_2_O, air-dried in a biosafety cabinet, and scanned on an InnoScan 710 Microarray Scanner (Innopsys, France) at 2 μm/pixel resolution and 532 nm wavelength. Data analysis was conducted using Mapix 8.5.0.

#### Caspase 3/7 activity assay

PRV7S-infected THP-1 and K562 cells were assessed for caspase-3/7 activity using Invitrogen™ CellEvent™ Caspase-3/7 Green ReadyProbes™ Reagent (Thermo Fisher Scientific, USA), following the manufacturer’s protocol. Similar to the PI viability assay, 1 × 10^4^ cells were seeded in each well of a 96-well dark plate, infected with PRV7S at MOIs of 0.1 and 10, and incubated for 1, 3, and 5 dpi. Approximately 35 μL (1 drop) of CellEvent™ Caspase-3/7 Green ReadyProbes™ Reagent was added to each well and incubated under standard cell culture conditions. Fluorescence readings were taken at 30 and 60 min post-incubation using a Varioskan™ LUX multimode microplate reader, with an excitation wavelength of 502 nm and an emission wavelength of 530 nm (suitable for GFP and FITC filters). Each experiment was performed in triplicate, with a total of three independent experiments conducted. The optimisation of caspase-3/7 activity assay using lithium chloride as a positive control (apoptosis inducer) was included in Extended Data Fig. 1f.

### PRV7S infection in AML-M5 human induced pluripotent stem cell

#### AML-M5 human induced pluripotent stem cell (hiPSC) culture

The AML-M5-hiPSC cell line, hereafter referred to as hiPSC, was previously generated by Chiew et al. (2017)^17^. This hiPSC model requires a feeder-dependent culture system utilising mouse embryonic fibroblasts (MEFs) as the feeder layer. Primary MEFs, pretreated with Mitomycin-C (10 μg/mL) or commercially obtained Gibco MEFs (Thermo Fisher Scientific, USA), were thawed and seeded into 6-well plates that had been pretreated with 0.1% gelatin at least one day before thawing and culturing the hiPSCs. MEFs were cultured in high-glucose DMEM supplemented with non-essential amino acids (NEAA), GlutaMAX, 10% foetal bovine serum (FBS), and 0.1% penicillin/streptomycin antibiotics.

For hiPSC culture, DMEM-F12 supplemented with 20% Gibco KnockOut Serum Replacement (Thermo Fisher Scientific, USA), 0.02 mM β-mercaptoethanol, and 10 ng/mL Gibco Human FGF-basic (FGF-2/bFGF) recombinant protein (Thermo Fisher Scientific, USA) was used. A 10 μM ROCK inhibitor (Y-27632) may be applied during the first 24 h post-thawing (not exceeding 24 h). When subculturing hiPSCs, a blade and scalpel were utilised for cutting and trimming the hiPSCs, avoiding chemical or enzymatic detachment techniques (e.g., trypsin, versene, and accutase).

#### The ability of hiPSC to differentiate spontaneously

Initially, healthy and undifferentiated hiPSCs were cut and transferred to a non-adherent, non-tissue culture (TC)-treated 6-well plate. The hiPSCs were then maintained in DMEM-F12 without FGF-2. Within a few days, the formation of embryoid bodies was observed, followed by the spontaneous differentiation of these embryoid bodies into adherent cells. Results of spontaneous differentiation assay were included in Extended Data Fig. 2a, b.

#### Immunofluorescence staining of hiPSC pluripotency markers

The expression of pluripotency markers (SSEA-4, TRA-1-81, OCT3/4, SOX2, and TRA-1-60) and the endoderm marker SOX17 in hiPSCs were determined using the immunofluorescence (IF) staining method as previously described^17^. Briefly, hiPSCs were seeded on a feeder-coated 24-well plate and subsequently fixed with 4% (v/v) paraformaldehyde (Thermo Fisher Scientific, USA) at room temperature (RTP) for 15 to 60 min. The fixed cells were then washed with PBS containing 1% Bovine Serum Albumin (BSA). For intracellular nuclear markers such as OCT3/4, an additional permeabilization step was performed using 0.2% Triton X-100 (Merck, Germany) for 15 min before IF staining. After permeabilization, the hiPSCs were blocked with either 10% rabbit serum or Blocker™ BSA (Thermo Fisher Scientific, USA) at RTP for 1 h. Following blocking, the cells were washed with 1% BSA/PBS and incubated with a dilution of fluorochrome-conjugated mouse anti-human antibodies (FITC-SOX17, FITC-TRA-1-81, FITC-SSEA4, PE-TRA-1-60, PE-SOX2, and PE-OCT3/4) ranging from 1:50 to 1:200 for 2 h at RTP or overnight at 4 °C. After incubation, the cells were washed several times with 1% BSA/PBS and examined under a ZEISS fluorescence microscope. Optionally, 1 mg/mL DAPI (Thermo Fisher Scientific, USA) can be employed as a counterstain for fixed cells, while 1 mg/mL Hoechst 33342 (Thermo Fisher Scientific, USA) can be used for live cell staining. A Nikon AX confocal microscope may be utilised if more than one fluorochrome-conjugated antibody or stain is employed, particularly when the emission wavelengths of the fluorochromes overlap or for 3-dimensional (3D) imaging. The results of IF were included in Extended Data Fig. 2c.

#### Oncolytic activities of PRV7S in hiPSC

The study of persistent infection (including TCID_50_ assays and qRT-PCR), MTT cell viability assay and Caspase 3/7 activity assay were performed as described above. Microscopy evaluation was performed using the EVOS™ XL Core Imaging System (Thermo Fisher Scientific, USA). Besides, Hoechst 33342 and PI stainings were performed on both the negative control and PRV7S-infected hiPSC colonies, which were subsequently examined under a confocal microscope. Additionally, healthy hiPSC colonies were also stained with Hoechst 33342, MitoSpy™ Red CMXRos (BioLegend, USA), ActinGreen™ 488 ReadyProbes™ Reagent (Thermo Fisher Scientific, USA) to view the intermediate filament and mitochondria (Extended Data Fig. 2d). A comparative analysis was performed between ROCK inhibitor-treated and non-treated hiPSC groups. However, no significant differences were observed between these two groups (data not shown).

### The safety profile of PRV7S in BALB/c mice

#### Ethics statement

All animal studies and procedures were approved by the IMU Joint-Committee on Research and Ethics (IMUJC) (BMS I-2019(10)). IMUJC guidelines for the proper and humane use of animals in biomedical research were followed strictly. The in vivo study also adhered strictly to the ARRIVE and AWERB guidelines.

#### Animal model and housing condition

Female BALB/c mice, approximately 5–6 weeks old and weighing approximately 16 g and above, were used. All mice were immunocompetent and in good health at the start of the study. The mice were housed in individually ventilated cages (IVC) with a 12-h light/dark cycle, under controlled conditions at approximately 23 °C. Each group contained six mice, with fresh bedding, food, and water provided throughout the study.

#### Inoculation of PRV7S into BALB/c mice and sample collection

The experimental design was adapted from previous studies^37–40,91^. The intraperitoneal (IP) and intranasal (IN) routes of administration were selected to investigate whether the virus can spread to other organs from different injection sites or potentially cause systemic infection. Additionally, pathological damage, if present, could be confirmed through histological examination. Furthermore, we aimed to determine whether PRV7S could persist in mice up to 14 dpi, with or without causing significant pathological damage. BALB/c mice (N = 6 per group) were inoculated with a single dosage of approximately Log 9 TCID_50_/mL of PRV7S or treated with Dulbecco’s Modified Eagle Medium (DMEM) as a vehicle control. Anaesthesia was administered using isoflurane via open-drop, nose-cone methods or with a vaporizer, with each mouse closely monitored throughout. Following anaesthesia, mice were inoculated intranasally or intraperitoneally with PRV7S or DMEM. Body weight was recorded daily.

At 14 dpi, mice were euthanized with an overdose of isoflurane, and organs were collected for histopathological analysis and RT-PCR detection. Notably, cervical dislocation was performed to confirm euthanasia. Organs were fixed in 10% neutral-buffered formalin for histopathological examination, while samples fixed in 95% ethanol were used for RT-PCR analysis.

Endpoint criteria included severe physiological or behavioural changes (e.g., blood in urine or faeces, significant weight loss, or breathing difficulties). If any significant indicator was observed, the mouse was immediately sacrificed via anaesthesia overdose. However, all mice were euthanized at 14 dpi regardless of clinical condition.

#### Statistics and reproducibility

All statistical analyses were performed using Python, R or GraphPad Prism 10 (GraphPad, Dotmatics). Unless specified in the figure description, statistical significance was determined using the ANOVA test or two-tailed unpaired Student’s *t*-test. Tukey’s honestly significant difference test was performed as a statistical hypothesis test to correct for multiple comparisons. *P* values < 0.05 were defined as statistically significant. No data were excluded from the analyses.

#### Reporting summary

Further information on research design is available in the (Nature Portfolio Reporting Summary) linked to this article.

## Supplementary Information

Supplementary Information 1.

Supplementary Information 2.

Supplementary Information 3.

Supplementary Information 4.

Supplementary Information 5.

Supplementary Information 6.

## Data Availability

The datasets generated and analysed in this study are available in the Gene Expression Omnibus (GEO) repository under the series record GSE286527 (accession numbers: GSM8728952 – GSM8728959) (https://www.ncbi.nlm.nih.gov/geo/query/acc.cgi?acc=GSE286527). All other data supporting the findings of this study are available from the corresponding author upon reasonable request.

## Code availability

In-house R scripts (version 4.2.3) and the Illumina DRAGEN RNA pipeline (available from Illumina: https://sapac.illumina.com/products/by-type/informatics-products/basespace-sequence-hub/apps/edico-genome-inc-dragen-rna-pipeline.html) were used in the transcriptomic analysis.
