# Supplementary material for: Safety profile of sikamat virus and its oncolytic potential in leukemic cells and cancer stem cells
Source: Sci Rep. 2025 Apr 22;15:13817. doi: 10.1038/s41598-025-96061-z (PMC12012088; doi:10.1038/s41598-025-96061-z)
Supplement: Supplementary file 1 — Supplementary Information 1. [file 41598_2025_96061_MOESM1_ESM.docx]

******Extended Data Fig. 1:** Transcriptome profiling and caspase 3/7 activity assay positive controls

**c**

**b**

**a**

**d**

**f**

**e**

**a**, Overview of Illumina DRAGEN RNA pipeline. **b**, Sample quality check. RNA concentration was measured using Qubit Fluorometer 4.0 (RNA HS); RNA purity was determined using Implen NanoQuant Spectrophotometer; RNA quality was determined using LabChip GX Touch Nucleic Acid Analyzer (RNA Assay). **c**, Representative image of electrophoresis of the samples. Ribosomal 28S (upper band, red color) and 18S (lower band, green color) RNAs. **d**, Library quality check metrics. DNA concentration was measured using Qubit Fluorometer 4.0; Average library fragment size was determined using LabChip GX Touch Nucleic Acid Analyzer. **e**, Electronic DNA electrophoresis gel (LabChip GX Touch) showing DNA library products. **f**, Lithium chloride (LiCl), known to induce apoptosis, was used as a positive control for the caspase-3/7 activity assay in THP1 and Vero cells. Caspase activity significantly increased in LiCl-treated THP1 and Vero cells, as well as in PRV7S-infected Vero cells, compared to negative controls. Data are presented as mean ± SD. Three independent experiments were performed, each with four replicates. Statistical significance was calculated using one-way ANOVA, followed by Tukey’s test. *p<0.05, **p<0.01, ****p<0.0001.
