# Supplementary material for: Safety profile of sikamat virus and its oncolytic potential in leukemic cells and cancer stem cells
Source: Sci Rep. 2025 Apr 22;15:13817. doi: 10.1038/s41598-025-96061-z (PMC12012088; doi:10.1038/s41598-025-96061-z)
Supplement: Supplementary file 2 — Supplementary Information 2. [file 41598_2025_96061_MOESM2_ESM.docx]

**Extended Data Fig. 2:** Spontaneous differentiation assay, pluripotency marker immunofluorescence staining, and confocal microscopy.

**
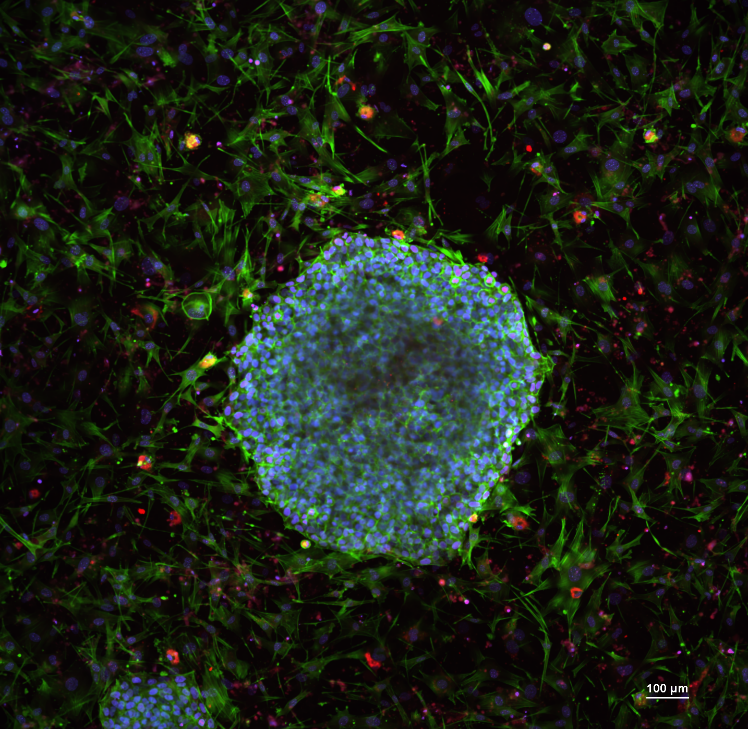
**
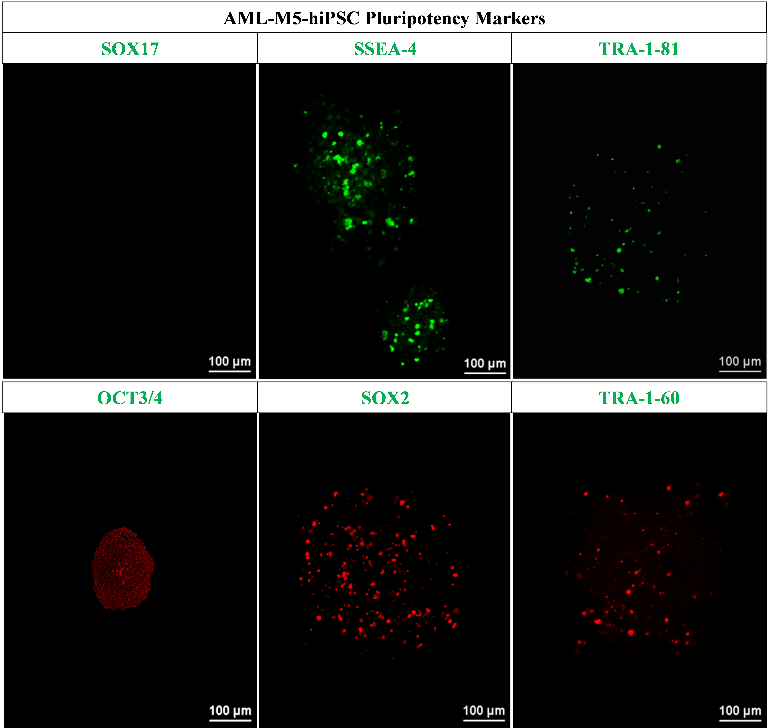

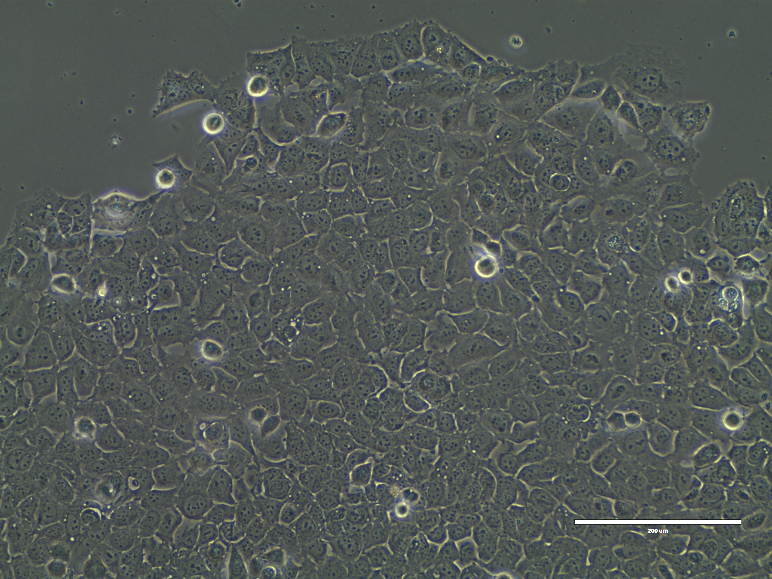

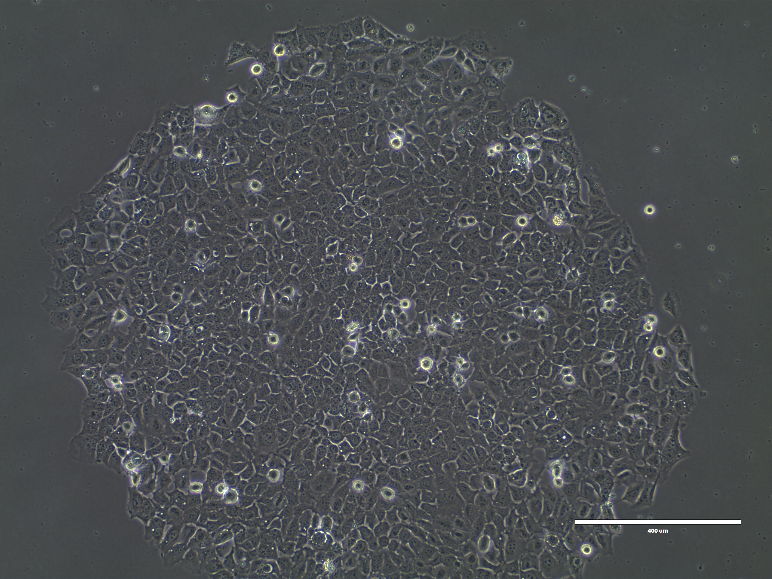

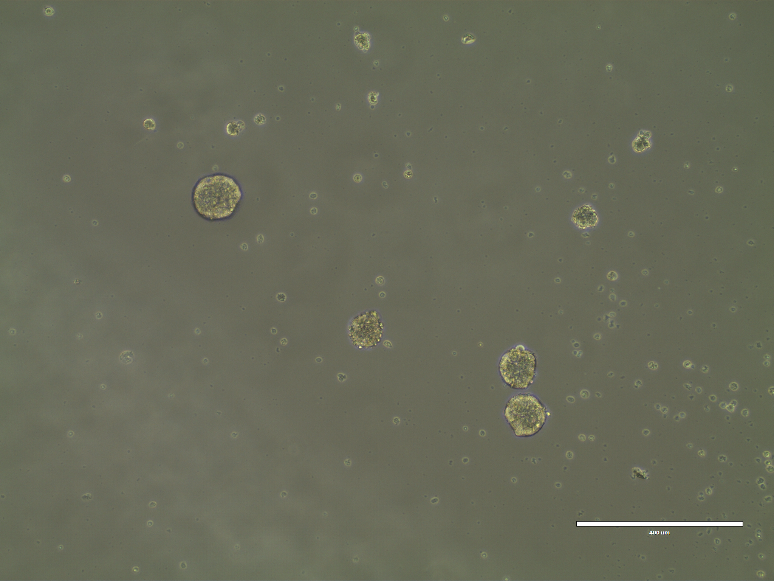

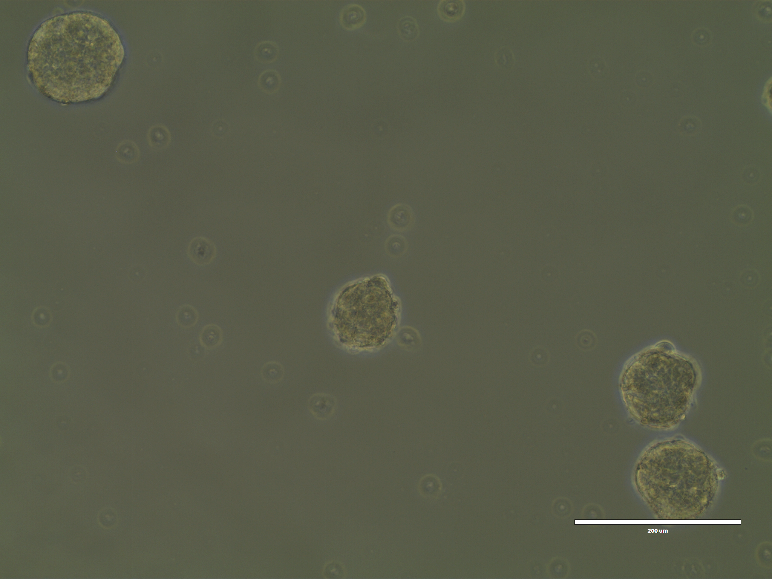


**d**

**a**

**c**

**b**

**a**, Formation of suspension embryoid bodies can be observed during the first 5 days of spontaneous differentiation assay. Scale bars: 400 μm (left), 200 μm (right). **b**, Usually, differentiated adherent cells can be observed from day 5 and onwards of spontaneous differentiation assay. Scale bars: 400 μm (left), 200 μm (right). **c**, The immunofluorescence staining of hiPSC pluripotency markers. Scale bars: 100 μm (all micrographs). **d**, High-resolution micrograph of healthy hiPSC colonies captured using a Nikon AX confocal microscope. The cells were stained with Hoechst 33342, MitoSpy Red CMXRos and ActinGreen 488 ReadyProbes Reagent. Scale bar: 100 μm.
