# Supplementary material for: Safety profile of sikamat virus and its oncolytic potential in leukemic cells and cancer stem cells
Source: Sci Rep. 2025 Apr 22;15:13817. doi: 10.1038/s41598-025-96061-z (PMC12012088; doi:10.1038/s41598-025-96061-z)
Supplement: Supplementary file 3 — Supplementary Information 3. [file 41598_2025_96061_MOESM3_ESM.pdf]

Supplementary Fig. 1: Global cancer statistics 2022

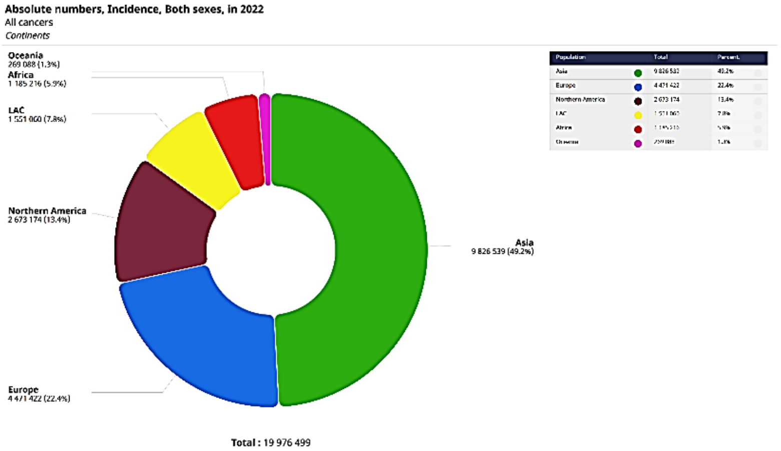

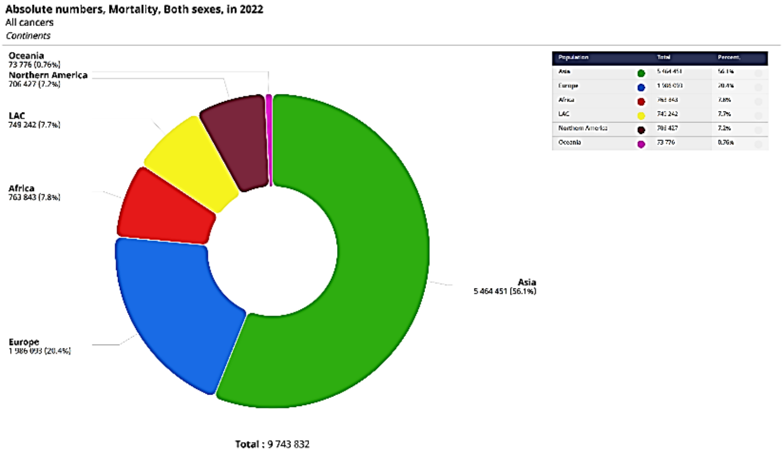

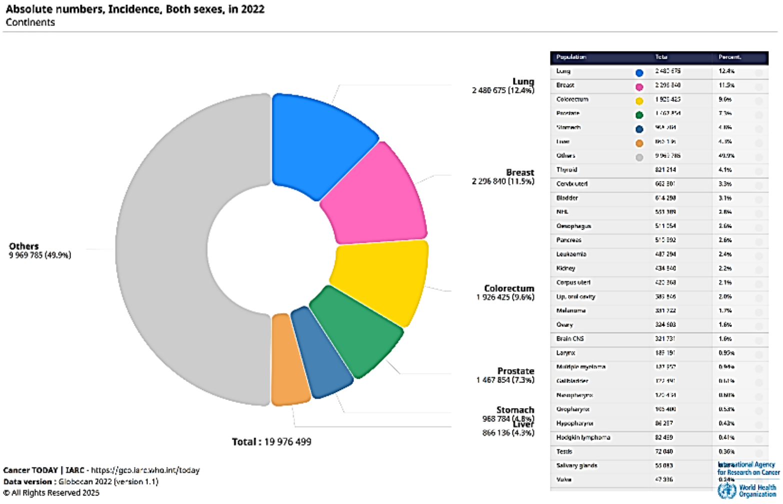

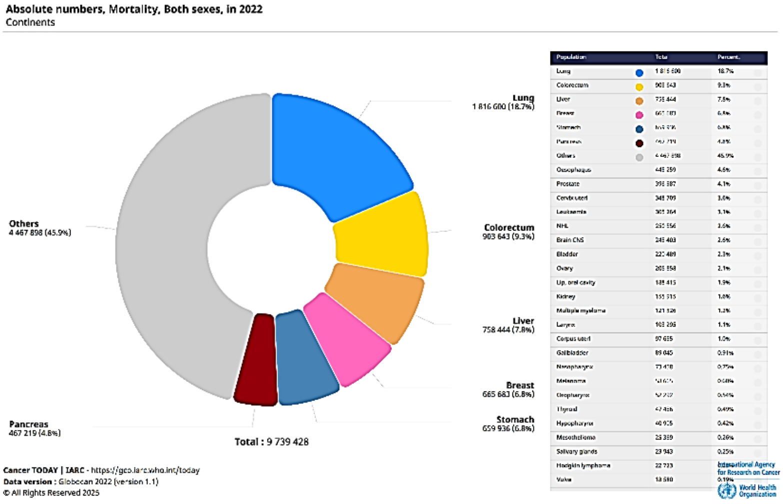

Absolute numbers, Incidence, Both sexes, in 2022
Leukaemia
Continents

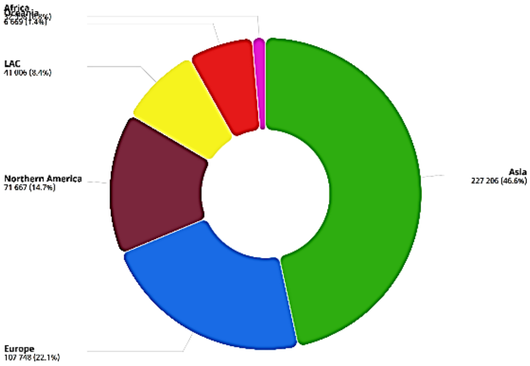

Population	Total	Percent.
Asia	227 206	46.6%
Europe	107 748	22.1%
Northern America	71 667	14.7%
LAC	41 006	8.4%
Africa	[illegible]	6.8%
Oceania	6 629	1.4%

Absolute numbers, Mortality, Both sexes, in 2022
Leukaemia
Continents

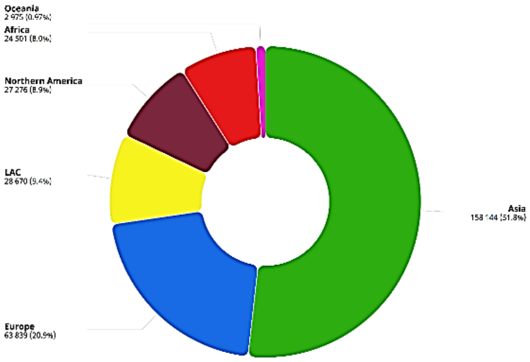

Population	Total	Percent.
Asia	158 144	51.8%
Europe	63 839	20.9%
LAC	28 670	9.4%
Northern America	27 276	8.9%
Africa	24 501	8.0%
Oceania	2 975	0.97%

Absolute numbers, Incidence, Both sexes, in 2022
Leukaemia
Countries

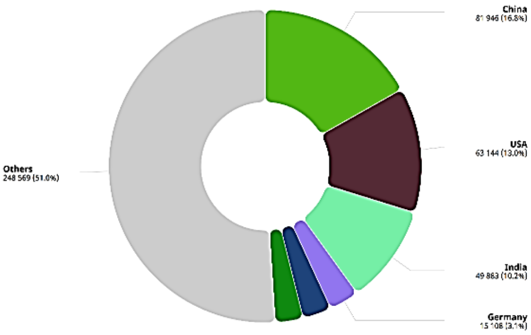

Population	Total	Percent.
China	81 946	16.8%
USA	63 144	13.0%
India	49 883	10.2%
Germany	15 108	3.1%
Russian Federatio.	14 495	3.0%
Indonesia	13 959	2.9%
Others	248 569	51.0%
Japan	13 572	2.8%
France	13 525	2.8%
Brazil	11 859	2.4%
Italy	10 799	2.2%
United Kingdom	10 755	2.2%
Canada	8 503	1.7%
Pakistan	7 722	1.6%
Mexico	7 457	1.5%
Philippines	7 026	1.4%
Iran (Islamic Rep.	6 485	1.3%
Türkiye	6 344	1.3%
Egypt	6 194	1.3%
Spain	5 976	1.2%
Viet Nam	5 789	1.2%
Australia	5 338	1.1%
Poland	5 152	1.1%
Ethiopia	4 721	0.97%
Thailand	4 427	0.91%
Republic of Korea	3 781	0.78%
Ukraine	3 522	0.72%
Colombia	3 399	0.70%
The Netherlands	3 136	[illegible]
Argentina	2 998	0.62%

Cancer TODAY | IARC - https://gco.iarc.who.int/today
Data version : Globocan 2022 (version 1.1)
© All Rights Reserved 2025

Absolute numbers, Mortality, Both sexes, in 2022
Leukaemia
Countries

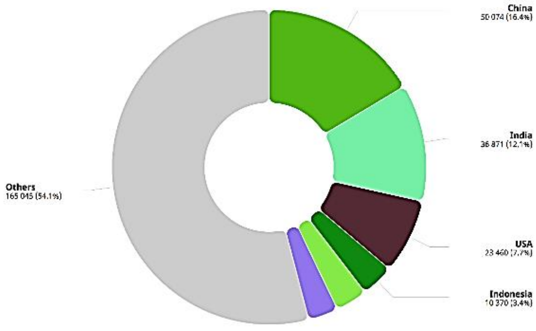

Population	Total	Percent.
China	50 074	16.4%
India	36 871	12.1%
USA	23 460	7.7%
Indonesia	10 370	3.4%
Japan	10 066	3.3%
Germany	9 378	3.1%
Others	165 045	54.1%
Brazil	8 790	2.9%
Russian Federatio.	8 224	2.7%
France	7 387	2.4%
Italy	7 197	2.4%
Iran (Islamic Rep.	5 499	1.8%
Philippines	5 416	1.8%
Pakistan	5 345	1.8%
United Kingdom	5 335	1.7%
Mexico	5 126	1.7%
Türkiye	[illegible]	[illegible]
Egypt	[illegible]	1.4%
Viet Nam	4 330	1.4%
Canada	3 808	1.2%
Spain	3 617	1.2%
Poland	3 534	1.2%
Ethiopia	3 482	1.1%
Thailand	3 194	1.0%
Colombia	2 428	0.80%
Bangladesh	[illegible]	0.75%
Australia	[illegible]	[illegible]
Republic of Korea	2 191	[illegible]
Argentina	2 111	[illegible]
Ukraine	2 106	0.69%

Cancer TODAY | IARC - https://gco.iarc.who.int/today
Data version : Globocan 2022 (version 1.1)
© All Rights Reserved 2025

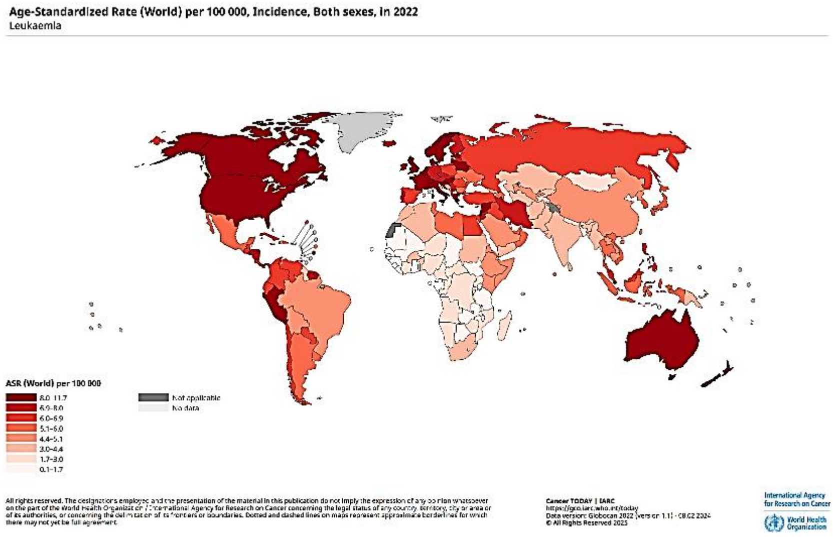

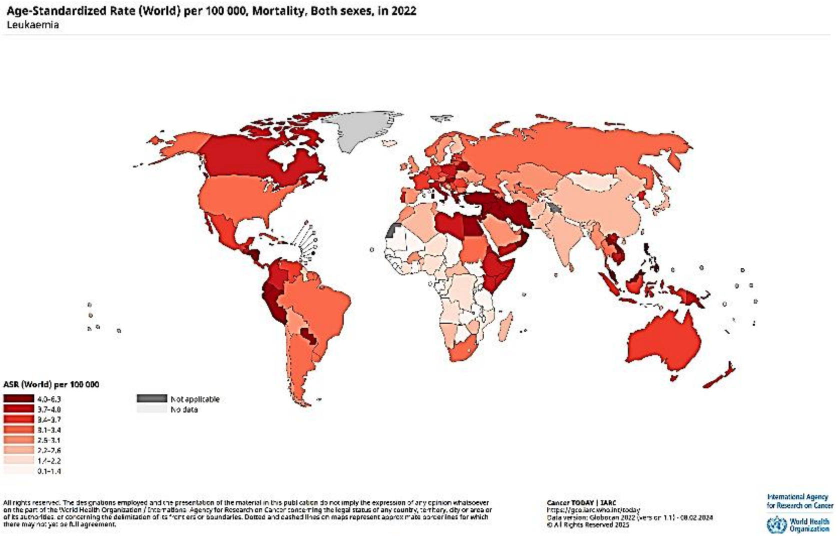

Figures are visualising the statistics of all cancers or leukaemia only over the world. The data is presented based on either estimated incidence or mortality rate.

Reference

1. Cancer Today. Dataviz. *Global Cancer Observatory*. Available at: https://gco.iarc.fr/today/en/dataviz (Accessed: 21 February 2025).
