# Supplementary material for: Safety profile of sikamat virus and its oncolytic potential in leukemic cells and cancer stem cells
Source: Sci Rep. 2025 Apr 22;15:13817. doi: 10.1038/s41598-025-96061-z (PMC12012088; doi:10.1038/s41598-025-96061-z)
Supplement: Supplementary file 4 — Supplementary Information 4. [file 41598_2025_96061_MOESM4_ESM.pdf]

Supplementary Fig. 2: Complete cell death pathway of PRV7S-infected THP1 cells

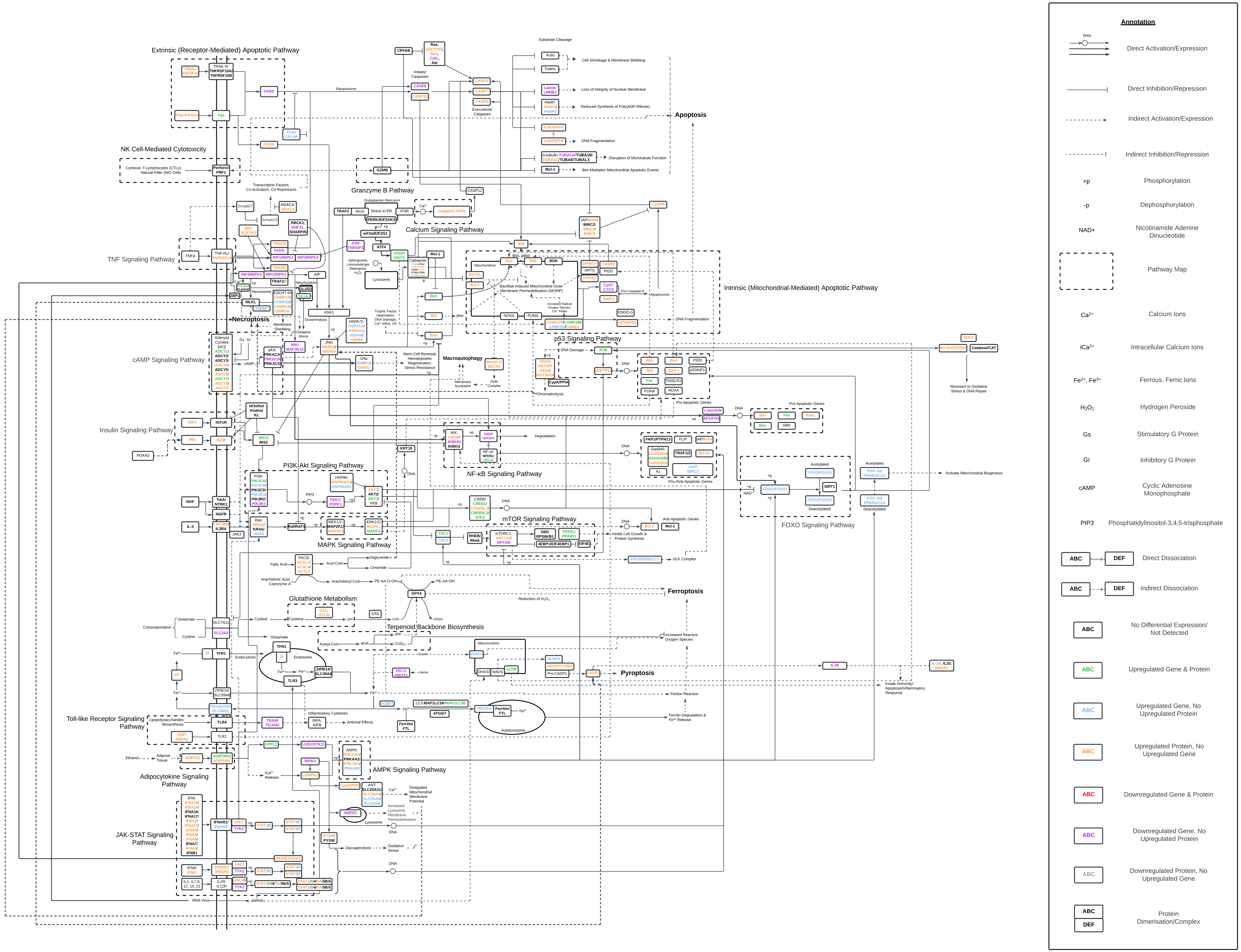

This complete cell death pathway map is constructed based on KEGG database.
