# Supplementary material for: Safety profile of sikamat virus and its oncolytic potential in leukemic cells and cancer stem cells
Source: Sci Rep. 2025 Apr 22;15:13817. doi: 10.1038/s41598-025-96061-z (PMC12012088; doi:10.1038/s41598-025-96061-z)
Supplement: Supplementary file 5 — Supplementary Information 5. [file 41598_2025_96061_MOESM5_ESM.pdf]

# Supplementary Fig. 3: Complete cell death pathway of PRV7S-infected K562 cells

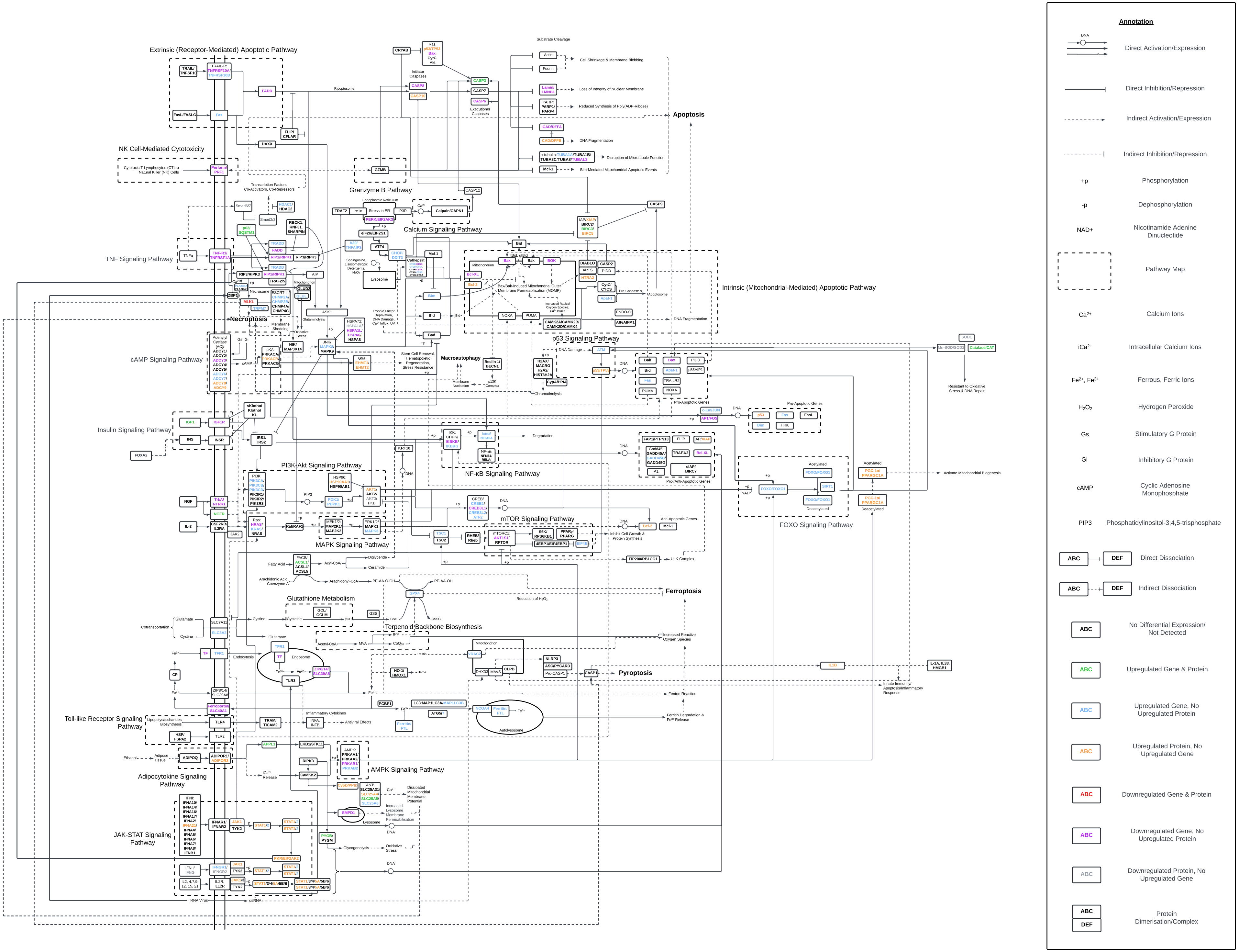


This complete cell death pathway map is generated based on KEGG database.
