# Supplementary material for: Safety profile of sikamat virus and its oncolytic potential in leukemic cells and cancer stem cells
Source: Sci Rep. 2025 Apr 22;15:13817. doi: 10.1038/s41598-025-96061-z (PMC12012088; doi:10.1038/s41598-025-96061-z)
Supplement: Supplementary file 6 — Supplementary Information 6. [file 41598_2025_96061_MOESM6_ESM.docx]

**Supplementary Table 1.** French-American-British (FAB) classification of acute myeloid leukaemia (AML). It includes features of each FAB subtype

| FAB Classification Subtype | Features | References |
| --- | --- | --- |
| AML-M0 | - Near 100% immature myeloblasts out of non-erythroid cells. (morphological diagnosis is indefinite as it is indifferentiable with ALL subtypes) - Blasts with peroxidase/Sudan Black negative or less than 3% myeloblasts with myeloperoxidase/Sudan Black B positive. | (1-4) |
| AML-M1 | - At least 90% of myeloblasts out of non-erythroid cells. - At least 3% blasts with myeloperoxidase/Sudan Black B positive. - At least 10% matured granulocytes. | (1,2) |
| AML-M2 | - 30% to 89% of myeloblasts out of non-erythroid cells. - Less than 20% matured monocytes with non-specific esterase-butyrate positive. - More than 10% matured granulocytes. |  |
| AML-M3 | - Promyelocytes with hypergranularity and presence of Auer rods. - Non-specific esterase-butyrate is negative in promyelocytes. |  |
| AML-M4 | - At least 30% myeloblasts out of non-erythroid cells. - At least 20% of monocytes from myeloblasts with different levels of maturation. - Less than 80% monocytes are non-specific esterase-butyrate positive. |  |
| AML-M4 with eosinophilia | - At least 5% of eosinophils out of non-erythroid cells. - Eosinophils are specifically chloroacetate esterase and periodic acid Schiff positive. |  |
| AML-M5a | - At least 80% of monoblasts out of non-erythroid cells. |  |
| AML-M5b | - Less than 80% monoblasts out of non-erythroid cells, at least 20% promonocytes and monocytes. - More than 80% monocytes are non-specific esterase-butyrate positive. |  |
| AML-M6 | - At least 30% myeloblasts or promyeloblasts out of non-erythroid cells. - At least 50% erythroblasts. |  |
| AML-M7 | - Presence of thrombocyte glycoproteins CD41, CD61 and CD62. (Determined by flow cytometry) | (4) |

**Supplementary Table 2.** Updated World Health Organisation (WHO) classification of acute myeloid leukaemia (AML), 5^th^ edition (2022). It includes features of each WHO subtype and indication of changes from the 4^th^ edition

| WHO Classification Subtype | Features | References |
| --- | --- | --- |
| AML with Defining Genetic Abnormalities | | (5-8) |
| APL with PML::RARA Fusion  (Formerly termed AML with t(15;17)(q22;11-12); RARA/PML fusion gene) | - Encodes RARα/PML fusion oncoprotein. - Clumps of Auer rods observed in immature granulocytes. - Associated with cytogenetics of t(15;17)(q22;11-12). - Promyelocytes with hypergranularity express HLA-DR with downregulated CD34. Promyelocytes with microgranularity expresses CD34 and CD2. |  |
| AML with RUNX1::RUNX1T1 Fusion  (Formerly termed AML with t(8;21)(q22;q22); RUNX1/RUNXT1 fusion gene) | - Encodes RUNX1/RUNXT1 fusion oncoprotein. - Presence of matured myelocytes with abnormal granules. - Associated with cytogenetics of t(8;21)(q22;q22) - Myeloblasts express CD13, CD34 and CD56. |  |
| AML with CBFB::MYH11 Fusion  (Formerly termed AML with inv(16)(p13;q22); CBFB/MYH11) | - Encodes CBFB/MYH11 fusion oncoprotein. - Associated with cytogenetics of inv(16)(p13;q22) - Eosinophilia in marrow with large dark purple granules. - Presence of differentiated monocytes. |  |
| AML with BCR::ABL1 Fusion | - Encodes BCR/ABL1 fusion oncoprotein. - Presence of more than 20% myeloblasts is compulsory. (newly added criterion) - Myeloblasts express CD7, CD19 and TdT. |  |
| AML with RBM15::MRTFA Fusion  (Formerly termed AML with RBM15::MKL1) | - Encodes RBM15/MRTFA fusion oncogene. - Presence of differentiated megakaryocytes expressing CD41, CD42b, and CD61 - Myelofibrosis is observed. |  |
| AML with DEK::NUP214 Fusion | - Encodes DEK/NUP214 fusion oncogene. - Differentiation of multiple myeloid lineage cells. - Frequently with basophilia. |  |
| AML with CEBPA Mutation | - Associated with mutations of biallelic CEBPA and bZIP region of smbZIP-CEBPA gene. - Presence of more than 20% myeloblasts is compulsory. (newly added criterion) - Presence of differentiated of granulocytes and megakaryocytes. |  |
| AML with NPM1 Mutation  (Formerly termed AML with normal cytogenetics and NMP mutation) | - Presence of more than 10% of myeloblasts. - Cup-like nuclei appearance is observed. - Associated with no cytogenetic abnormalities. | (5,6,9) |
| AML with KMT2A Rearrangement  (Former termed AML with t(9;11)(p22;q23), KMT2A/MLLT3) | - Encodes fusion oncoprotein of KMT2A commonly partnered with MLLT3, MLLT10, AFDN, ELL. - Presence of high number of myeloblasts with monocytes in adult patients whereas low number of myeloblasts and megakaryocytes in paediatric patients. | (5-8) |
| AML with MECOM Rearrangement | - Encodes MECOM fusion oncoprotein partnered with prevalently GATA2. - Presence of differentiated megakaryocytes. |  |
| AML with NUP98 Rearrangement | - Associated with mutations of FLT_3_-ITD and WT1 genes. - Presence of megakaryoblasts and erythroid cells in paediatric patients. |  |
| AML-Myelodysplasia Related (AML-MR)  (Formerly classified as AML with Myelodysplastic Syndrome-Related Changes, this classification is no longer used.) | - Presence of at least 20% myeloblast with cytogenetics of del(5q), del(7q), del(11q), del(12q), del(13q), del(17p), i(17q), and idic(X)(q13). (Newly added features) - Associated with genetic mutations of ASXL1, BCOR, EZH2, SF3B1, SRSF2, STAG2, U2AF1 and ZRSR2. (Newly added features) |  |
| AML with other defined genetic abnormalities | Category that includes new arising AML subtypes with distinguished features. |  |
| AML Defined by Differentiation (Removal of AML, not otherwise specified subtype) - Subclassification | |  |
| AML with minimal differentiation | - Similar to AML-M0 (FAB Classification) features. |  |
| AML without maturation | - Similar to AML-M1 (FAB Classification) features. - Presence of less than 10% matured granulocytes. |  |
| AML with maturation | - Similar to AML-M1 (FAB Classification) features - Presence of more than 10% matured granulocytes. - Myeloblasts express CD13, CD33, CD117, MPO. |  |
| Acute Basophilic Leukaemia | - Presence of differentiated basophils or immature basophils with metachromasia on toluidine blue staining. - Presence of myeloblasts with myeloperoxidase, Sudan Black B, Non-specific Esterase-Butyrate negative. - Myeloblasts have no expression of CD117 like mast cells. |  |
| Acute Myelomonocytic Leukaemia | - Presence of at least 20% promonocytes and monocytes and matured granulocytes - Presence of at least 3% blasts with myeloperoxidase positive. |  |
| Acute Monocytic Leukaemia | - Presence of at least 80% monocytes or promonocytes with non-specific esterase-butyrate positive. - Presence of less than 20% matured granulocytes. - Blasts express CD11c, CD14, CD36, CD64. |  |
| Acute Erythroid Leukaemia | - Presence of at least 30% proerythroblasts and 80% erythroid cells in bone marrow. |  |
| Acute Megakaryoblastic Leukaemia | - Similar to AML-M7 (FAB Classification) features. |  |

APL, Acute Promyelocytic Leukaemia; AML, Acute Myeloid Leukaemia.

**Supplementary Table 3.** French-American-British (FAB) classification of chronic myeloid leukaemia (CML). It includes features of each FAB subtype

| **FAB Classification Subtype** | **Features** | **Reference** |
| --- | --- | --- |
| Chronic Granulocytic Leukaemia (CGL) | - Positive or negative Ph chromosome. - Positive rearrangement of breakpoint cluster region (BCR) on q arm of chromosome 22. - More than 20% of undifferentiated granulocytes and blast cells fewer than 2%. - At least 2% of matured basophils and less than 3% of matured monocytes. - Absence of granulocytic dysplasia. | (3) |
| Chronic Myelomonocytic Leukaemia (CMML) | - Less than 10% of undifferentiated granulocytes and less than 2% of blast cells. - Less than 2% of matured basophils and presence of 3-10% of matured monocytes. - Presence of granulocytic dysplasia. - Averagely 11% of blast cells present in bone marrow. | (3,10) |
| Atypical Chronic Myeloid Leukaemia (aCML) | - Presence of 10-20% of undifferentiated granulocytes and more than 2% of blast cells. - Less than 2% of matured basophils and at least 3% of matured monocytes. - High presence of granulocytic dysplasia. | (3) |

**Supplementary Table 4.** Updated World Health Organisation (WHO) classification of Ph positive chronic myeloid leukaemia (CML), 5^th^ edition (2022). It includes features of each WHO subtype and indication of changes from the previous edition

| **WHO Classification Subtype** | | **Features** | **Relative Prognosis** | **References** |
| --- | --- | --- | --- | --- |
| Ph Chromosome Positive CML | Chronic Phase | - Associated with cytogenetic t(9;22)(q34.1;q11.2). - Encodes for BCR-ABL1 fusion oncoprotein. - Presence of 100% cellularity in bone marrow with matured granulocytes and decreased presence of precursor cells. - Reticular fibers is observed in certain cases with increased megakaryocytes. | CML involved with additional genetic abnormalities generally has poor prognosis.  **From Better to Worse (Chronic Phase 🡪 Blastic Phase)** | (5,6,11,12) |
|  | Accelerated Phase  (**Excluded in 5^th^ edition**) | - Persistent or worsening leucocytosis (WBC > 10 X 10^9^/L) & no complete haematological response upon more than one TKI therapy. - Persistent or worsening splenomegaly. - Associated with cytogenetic t(9;22)(q34.1;q11.2) with additional genetic aberrations (presence of second Ph chromosome, isochromosome 17q, trisomy 8, trisomy 19), complex karyotypic profile, and chromosomal abnormalities in q26.2 position of chromosome 3. - Encodes for BCR-ABL1 fusion oncoprotein. - Persistent or worsening thrombocytosis (>1000 X 10^9^/L) or thrombocytopenia (<100 X 10^9^/L). - At least 20% peripheral basophils. - Presence of 10-19% of peripheral/bone marrow blasts.   (**Excluded in 5^th^ edition**). |  |  |
|  | Blastic Phase | - Associated with cytogenetic t(9;22)(q34.1;q11.2). - Encodes for BCR-ABL1 fusion oncoprotein. - At least 20% peripheral blast cells or extramedullary presence of blast cells. - Presence of increased peripheral/bone marrow lymphoblasts. (**Added criterion in 5^th^ edition**). |  |  |
| Myelodysplastic/  Myeloproliferative  Neoplasms | CMML | Listed in **Supplementary** **Table 3** | Not Discussed | (3,10) |
|  | aCML |  |  |  |

**Supplementary Table 5.** Clinical Trials of Oncolytic Viruses against Different Types of Malignancies

| Name | Treatment Combination | Tumour Type | Phase | Status (Accessed June 2024) | Outcome (Efficacy and Safety) | References |
| --- | --- | --- | --- | --- | --- | --- |
| DNA Viruses: Herpes Simplex Virus Type-1 (HSV-1) | | | | | | |
| G207 | RT | Glioma | I | Completed | - Tumour stabilization or partial response was reported in 66% of study participants. - G207 was generally well-tolerated and there were no indications of HSV encephalitis in patients. | (13)  NCT00157703 |
|  | None | Glioma, Astrocytoma, and Glioblastoma | I, II | Completed | - Efficacy of G207 in study participants during Phase I was not reported. - No severe adverse effects were reported and no indications of HSV encephalitis. | (14)  NCT00028158 |
|  | RT | Supratentorial Brain Tumours | I | Completed | - Tumour stabilization was observed in 58% of patients in 1 month. - Only low-grade adverse effects were reported. | (15)  NCT02457845 |
|  | RT | Cerebellar Brain Tumours | I | Active,  Not Recruiting | No outcome is reported currently. | No related publications currently.  NCT03911388 |
| NV1020 | CT | Metastatic Colon Cancer | I, II | Completed | - Tumour stability was observed in 50% of study participants in Phase I. - Tumour regression was observed in 64% of study participants. - The tumour stability rate was 68% in Phase II. - Mild pyrexia was reported in the majority of patients. - Dose-dependent toxicity was only confined to transient lymphocytopenia. | (16,17)  NCT00149396 |
| OrienX010 | MA  (JS001) | Melanoma | I | Unknown | No outcome is reported currently. | No related publications currently.  NCT04206358 |
|  | CT  (Dacarbazine) | Melanoma | II | Unknown | No outcome is reported currently. | No related publications currently.  NCT04200040 |
|  | MA  (Toripalimab) | Melanoma | I | Active, Not Recruiting | - 81% of patients were reported having pathologic remission. - Low-grade pyrexia is the most common adverse effect experienced by study participants. | (18)  NCT04197882 |
|  | None | Melanoma, Liver Cancer, Pancreatic Cancer, and Lung Cancer | I | Completed | No outcome is reported currently. | No related publications currently.  NCT01935453 |
| HF10 (TBI-1401) | None | Melanoma and Skin SCC | I | Completed | No outcome is reported currently. | No related publications currently.  NCT02428036 |
|  | MA  (Ipilimumab) | Melanoma | II | Completed | No outcome is reported currently. | No related publications currently.  NCT03153085 |
|  | MA  (Nivolumab) | Melanoma | II | Terminated | - 83% of study participants reported having a pathologic complete response. - 43% of study participants reported experiencing severe adverse effects. - All study participants were at risk of toxicities.   **The trial was terminated possibly due to the risk of severe toxicities.** | No related publications currently.  NCT03259425 |
|  | None | Head & Neck Cancer, Skin SCC, Breast Cancer and Melanoma | I | Completed | - Tumour stability was observed in 67% of study participants. - Tumour size was reduced by 45% after administration. - Only low-grade adverse effects were reported in study participants. | (19)  NCT01017185 |
|  | CT  (Gemcitabine,  Nab-Paclitaxel/  TS-1) | Pancreatic Cancer | I | Active, Not Recruiting | No outcome is reported currently. | No related publications currently.  NCT03252808 |
|  | MA  (Ipilimumab) | Metastatic  Melanoma | II | Completed | No outcome is reported currently. | No related publications currently.  NCT02272855 |
| HSV1716 | None | Rhabdomyosarcoma, Osteosarcoma, Ewing Sarcoma, Soft Tissue Sarcoma, Neuroblastoma, Wilm’s Tumour, Malignant Peripheral Nerve Sheath Tumour, and Clival Chordoma | I | Completed | - Tumour stability was observed in 78% of study participants on day 14 after intralesional administration. - Only low-grade and short-term adverse effects were reported in study participants. | (20)  NCT00931931 |
|  | None | Malignant  Pleural  Mesothelioma | I, II | Completed | - Tumour stability was observed in 67% of patients receiving a single dose and 2 doses. - Tumour stability was observed in 33% of patients receiving 4 doses. - Overall, the safety profile in patients receiving single and multiple doses was adequate. | (21)  NCT01721018 |
|  | GC  (Dexamethasone)  SP | Glioma | I | Terminated | No outcome is reported currently.  **The trial was terminated without clarification.** | No related publications currently.  NCT02031965 |
| Talimogene Laherparepvec  (T-VEC, Imlygic™) | CT  (Nivolumab) | Melanoma | II | Active, Not Recruiting | No outcome is reported currently. | (22)  NCT04330430 |
|  | None | Angiosarcoma | II | Terminated | - Efficacy is not evaluated. - All study participants were at risk of adverse effects.   **The trial was terminated due to funding cessation from the sponsor.** | No related publications currently.  NCT03921073 |
|  | None | Basal Cell Carcinoma, Skin SCC, Cutaneous Lymphoma, and Merkel Cell Carcinoma | I | Completed | No outcome is reported currently. | No related publications currently.  NCT03458117 |
|  | CT  (Melphalan) | Melanoma and Sarcoma | I, II | Completed | - Overall, 77% of study participants were reported having pathologic remission. - The combined therapy is generally well-tolerated and short-term adverse effects were reported in study participants. | (23)  NCT03555032 |
|  | MA  (Pembrolizumab) | Melanoma | II | Recruiting | No outcome is reported currently. | (24)  NCT03842943 |
|  | MA  (Pembrolizumab) | Liver Cancer, Liver Metastases, Cutaneous/Subcutaneous  Lymph Node, Breast Cancer, Skin SCC, and Colorectal Adenocarcinoma | I, II | Completed | - Pathologic response rate for liver cancers was not reported. - 10% of the study participants reported having a pathologic complete response for hormonal receptor-positive breast cancer and skin SCC. - 16% of the study participants reported having a pathologic complete response for triple-negative breast cancer. - 20% of the study participants were reported having a pathologic complete response for basal cell carcinoma - 11% of patients receiving only T-VEC were reported to have dose-limiting adverse effects | No related publications currently.  NCT02509507 |
|  | None | Early Melanoma | II | Suspended | No outcome is reported currently.  **The trial was suspended due to the low number of enrolled participants.** | No related publications currently.  NCT04427306 |
|  | None | Peritoneal Surface Malignancies and Ovarian Cancer | I | Completed | - Efficacy is not evaluated. - No dose-limiting adverse effects were reported. 2 out of 19 patients were reported experiencing moderate adverse effects.   **The trial results were submitted on 5^th^ May 2024.** | (25)  NCT03663712 |
|  | None | Malignant Melanoma | I | Completed | - The overall pathologic response rate is 11.1%. - Overall, the safety profile in patients receiving only T-VEC was adequate. The most common adverse effect was mild pyrexia. | (26)  NCT03064763 |
|  | RT | Melanoma and Merkel Cell Carcinoma | II | Completed | - The pathologic response rate for cutaneous metastases was at least 27% upon administration of T-VEC with or without radiotherapy. Combined therapy was more effective for cutaneous metastases. - The most prevalent mild adverse effect was pyrexia. | (27)  NCT02819843 |
|  | MA  (Ipilimumab) | Melanoma | I, II | Completed | - 53% of the study participants were reported having an overall response of complete response or partial response - There were no dose-limiting toxicities reported in study participants. | (28)  NCT01740297 |
|  | MA  (Ipilimumab, Nivolumab) | Breast Cancer | I | Terminated | No outcome is reported currently.  **The trial is terminated due to enrolment issues.** | No related publications currently.  NCT04185311 |
|  | None  (Comparison with GM-CSF) | Melanoma | III | Completed | - Overall pathologic response rate was greater in patients receiving only T-VEC (26.4%). - There were no life-threatening adverse effects reported in the study participants. | (19)  NCT00769704 |
|  | MA  (Atezolizumab) | Metastatic Triple Negative Breast Cancer and Metastatic Colorectal Cancer | I | Completed | - Overall, 10% of study participants reported having pathologic remission for triple-negative breast cancer. - There were no dose-limiting adverse effects reported in patients with triple-negative breast cancer whereas there was 17% of study participants with colorectal cancer had dose-limiting toxicities. | (29)  NCT03256344 |
|  | None  (Comparison with GM-CSF) | Melanoma | III | Completed | - 57% of study participants receiving T-VEC were reported having pathologic remission. - The most common adverse effect reported on study participants was chills. | No related publications currently.  NCT01368276 |
|  | SP | Melanoma | II | Completed | - The percentage of patients having a pathologic complete response after receiving T-VEC was higher (17.1%). - The most common adverse effect reported on study participants was flu-like illness, followed by pyrexia. | (30)  NCT02211131 |
| DNA Viruses: Poxvirus (Vaccinia Virus) | | | | | | |
| JX-594  (Pexastimogene Devacirepvec, Pexa-Vec) | None | Melanoma | I, II | Completed | No outcome is reported currently. | (31)  NCT00429312 |
|  | None | Neuroblastoma, Rhabdomyosarcoma, Lymphoma, Wilm’s Tumour, and Ewing’s Sarcoma | I | Completed | - Tumour stability was reported in most study participants. - The most common adverse effect was pyrexia, reported in 86% of administration episodes. | (32)  NCT01169584 |
|  | None | Hepatocellular Carcinoma | II | Completed | No outcome is reported currently. | No related publications currently.  NCT01171651 |
|  | CT  (Cyclophosphamide)  MA  (Avelumab) | Soft Tissue Sarcoma and Breast Cancer | I, II | Recruiting | No outcome is reported currently. | (33)  NCT02630368 |
|  | CT  (Irinotecan) | Colorectal Carcinoma | I, II | Completed | - 6% of study participants were reported to have an overall radiographic response - The most common adverse effect reported in study participants was pyrexia. | No related publications currently.  NCT01394939 |
|  | None | Hepatocellular Carcinoma | II | Completed | - The modified Choi response rate was reported to be 62% in a total of 26 study participants. - Treatment was generally well-tolerated by study participants at high and low doses. | (34)  NCT00554372 |
|  | None | Colorectal Carcinoma | II | Terminated | No outcome is reported currently.  **The trial is terminated without clarification.** | No related publications currently.  NCT01329809 |
|  | MA  (Ipilimumab) | Metastatic/  Advanced Tumour  (Colorectal Carcinoma, Melanoma, Head & Neck Tumours, Gastric Cancer, Triple Negative Breast Cancer, Mesothelioma) | I | Completed | No outcome is reported currently. | (35)  NCT02977156 |
|  | TT  (Sorafenib) | Hepatocellular Carcinoma | III | Completed | - The overall response rate was reported 19% in study participants receiving the combined therapy. - Severe adverse effects were reported in approximately half of the study participants. | (36)  NCT02562755 |
| vvDD-CDSR | None | Melanoma, Breast Cancer, Head & Neck SCC, and Liver Cancer | I | Completed | No outcome is reported currently. | No related publications currently.  NCT00574977 |
| GL-ONC1 | CT  MA  (Bevacizumab) | Ovarian Cancer, Peritoneal Carcinomatosis, Fallopian Tube Cancer | I, II | Completed | - 55% of study participants were reported having stable disease and partial response. - Only mild adverse effects were reported in study participants. | (37)  NCT02759588 |
|  | RT  CT  (Cisplatin) | Head & Neck Cancer | I | Completed | - 74% of study participants reported having no tumour progression in one year. - The most prevalent adverse effects were mild, including pyrexia, rigours, and fatigue. | (38)  NCT01584284 |
|  | MA  (Eculizumab) | Advanced Solid Organ Tumours | I | Terminated | No outcome is reported currently.  **The trial is terminated due to inadequate funding.** | No related publications currently.  NCT02714374 |
|  | None | Peritoneal Carcinomatosis | I, II | Completed | - 89% of study participants reported having an efficacious viral infection followed by oncolysis. - Only mild to moderate adverse effects were reported in study participants. | (39)  NCT01443260 |
|  | CT  MA  (Bevacizumab) | Ovarian Cancer, Fallopian Tube Cancer, and Peritoneal Cancer | III | Recruiting | No outcome is reported currently. | (37,39,40)  NCT05281471 |
| T601 | CT  (Flucytosine) | Advanced Solid Tumours (Gastric Cancer, Pancreatic Cancer, Hepatocellular Carcinoma) | I, II | Unknown | No outcome is reported currently. | No related publications currently.  NCT04226066 |
| DNA Viruses: Parvovirus H-1 | | | | | | |
| ParvOryx | None | Pancreatic Ductal Carcinoma | I, II | Completed | - 29% of study participants reported having an overall partial response. - Virotherapy demonstrated a very good safety profile and no dose-limiting adverse effects were reported. | (41)  NCT02653313 |
|  | None | Glioblastoma Multiforme | I, II | Completed | - 27% of study participants were reported having no tumour progression within 6 months. - No dose-limiting adverse effects were reported in study participants. | (42)  NCT01301430 |
| DNA Viruses: Adenovirus | | | | | | |
| ONCOS-102 (Previously termed CGTG-102) | CT  (Cyclophosphamide)  MA  (Durvalumab) | Colorectal Cancer, Ovarian Cancer and Appendiceal Cancer | I, II | Completed | - On average, 23% of study participants were reported having disease stability. - Mild to moderate adverse effects were reported in patients. | (43)  NCT02963831 |
|  | CT  (Cyclophosphamide)  MA  (Pembrolizumab) | Melanoma | I | Completed | - Overall, 35% of study participants reported having a complete response or partial response. - 53% of study participants were reported having tumour regression. - The common adverse effects reported in patients were pyrexia and chills. | (44)  NCT03003676 |
|  | CT  (Cyclophosphamide) | Malignant Solid Tumours  (Ovarian Cancer, Metastatic Colon Cancer, Adenocarcinoma in Sigma, Hepatocellular Carcinoma, Pulmonary Adenocarcinoma, Lung Mesothelioma, Rectal Adenocarcinoma, Serous Endometrial Cancer, Soft Tissue Sarcoma, Breast Cancer) | I | Completed | - 40% of study participants reported having disease stability in 3 months. - Only mild adverse effects were reported in patients including pyrexia and flu-like presentation. | (45)  NCT01598129 |
|  | IT  (DCVAC/PCa)  HT  (Abiraterone/  Enzalutamide)  CT  (Cyclophosphamide) | Prostate Cancer | I, II | Terminated | No outcome is reported currently  **The trial is terminated due to inadequate accrual.** | No related publications currently.  NCT03514836 |
| CELYVIR  (Bone Marrow-Derived Autologous MSCs incorporated with ICOVIR-5) | None | Metastatic Solid Tumours | I, II | Completed | - 22% of study participants were reported having disease stability. - Only low-grade fever is reported in study participants. | (46)  NCT01844661 |
|  | RT | Pontine Glioma and Medulloblastoma | I, II | Recruiting | No outcome is reported currently. | No related publications.  NCT04758533 |
|  | None | Uveal Melanoma | I, II | Not Yet Recruiting | No outcome is reported currently. | No related publications.  NCT05047276 |
| ICOVIR-5 | None | Melanoma | I | Completed | No outcome is reported currently. | No related publications  NCT01864759 |
| DNX-2401  (Previously termed Delta-24-RGD-4C) | SP | Brain Cancer | I | Completed | - 72% of study participants were reported having tumour regression. - No dose-limiting adverse effects were reported. | (47)  NCT00805376 |
|  | MA  (Pembrolizumab) | Glioma and Glioblastoma | II | Completed | - Overall clinical benefit rate in study participants was 56%. - Only low-grade adverse effects were reported in patients including brain oedema, headache, and fatigue. | (48)  NCT02798406 |
|  | CT  (Temozolomide) | Glioblastoma Multiforme | I | Completed | No outcome is reported currently. | No related publications currently.  NCT01956734 |
| BM-DNX-2401  (Bone Marrow-Derived Autologous MSCs incorporated with DNX-2401) | SP | Anaplastic Astrocytoma, Glioblastoma, Gliosarcoma and Malignant Glioma | I | Recruiting | No outcome is reported currently. | (49)  NCT03896568 |
| VCN-01 | CT  (Gemcitabine/ Abraxane®) | Pancreatic Adenocarcinoma | I | Completed | - Overall, 50% of study participants were reported having an objective response (complete response or partial response) - The common adverse effects were reported in average 76% of study participants including fever and flu-like symptoms. | (50)  NCT02045602 |
|  | IT  (huCART-meso cells) | Pancreatic Cancer and Serous Ovarian Cancer | I | Recruiting | - 67% of study participants reported having tumour stability. - No dose-limiting adverse effects and no neurotoxicity were reported.   **The trial is still in progress.** | (51)  NCT05057715 |
| CG0070  (Cretostimogene Grenadenorepvec) | None | Bladder Cancer | II | Completed | - Overall, 47% of study participants were reported having complete responses. - An adequate safety profile was reported in study participants as the most frequent treatment-related toxicity is urinary bladder spasms (36%). | (52)  NCT02365818 |
|  | None | Transitional Cell Carcinoma of Bladder | II, III | Terminated | No outcome is reported currently.  **The trial is terminated due to study design alteration.** | No related publications currently.  NCT01438112 |
|  | None | Bladder Cancer | II | Withdrawn | No outcome is reported currently.  **The trial is terminated due to study design alteration.** | No related publications currently.  NCT02143804 |
|  | MA  (Nivolumab) | Bladder Cancer | I | Active, Not Recruiting | - 53% of study participants reported having a pathologic response. - The combined therapy is generally well-tolerated. | (53)  NCT04610671 |
|  | None | Non-Muscle Invasive Bladder Cancer | I | Recruiting | No outcome is reported currently. | No related publications currently.  NCT06253845 |
|  | n-dodecyl-B-D-maltoside | Non-Muscle Invasive Bladder Cancer and Urothelial Carcinoma | III | Recruiting | No outcome is reported currently. | No related publications currently.  NCT06111235 |
| RNA Viruses: Picornavirus | | | | | | |
| Poliovirus (Lerapolturev/  PVSRIPO) | None | Melanoma | I | Completed | - 33% of study participants reported achieving objective responses. - 50% of study participants reported achieving a pathologic complete response. - Only low-grade pruritus was commonly reported in study participants. | (54)  NCT03712358 |
|  | None | Glioblastoma and Glioma | I | Completed | - The overall survival rate of study participants tapered off at 21% starting from 2 years after administration. - 69% of the study participants were reported experiencing low-grade adverse effects. | (55)  NCT01491893 |
|  | MA  (Nivolumab) | Melanoma | I | Withdrawn | No outcome is reported currently.  **The trial is withdrawn due to resubmission.** | No related publications currently.  NCT04125719 |
|  | None | Anaplastic Astrocytoma, Anaplastic Oligoastrocytoma, Anaplastic  Oligodendroglioma, Glioblastoma, Gliosarcoma, Atypical Teratoid/Rhabdoid Tumour of Brain, Medulloblastoma, Ependymoma, Embryonal Tumour of Brain, and Pleomorphic Xantho-astrocytoma of Brain | I | Completed | - Efficacy was not directly measured from study participants. - No lethal adverse effects were reported in the study participants. | (56)  NCT03043391 |
|  | CT  (Lomustine) | Supratentorial Glioblastoma | II | Not Yet Recruiting | No outcome is reported currently. | No related publications currently.  NCT06177964 |
| Seneca Valley Virus-001  (NTX-010) | CT  (Cyclophosphamide) | Adrenocortical Carcinoma, Gastrointestinal Carcinoid Tumour, Kidney Cancer, Neuroblastoma, Retinoblastoma, and Sarcoma | I | Completed | - 50% of study participants were reported achieving disease stability. - No association between treatment and toxicity was recorded. | (57)  NCT01048892 |
|  | Placebo | Lung Cancer | II | Terminated | **The trial is terminated due to no conclusive clinical benefit from treatment.** | (58)  NCT01017601 |
| Coxsackievirus A21 (CVA21/  CAVATAK™) | CT  (Mitomycin C) | Non-Muscle Invasive Bladder Cancer | I | Completed | - A complete pathologic response was observed in 1 study participant. - No significant adverse effects were reported in the study participants. | (59)  NCT02316171 |
|  | MA  (Pembrolizumab) | Non-Small Cell Lung Cancer and Bladder Cancer | I | Completed | - Overall, the objective response rate of study participants was 12%. - Moderate adverse effects were reported in study participants. Fatigue was reported in 36% of study participants. | (60)  NCT02043665 |
|  | MA  (Ipilimumab) | Uveal Melanoma and Liver Metastases | I | Completed | - 27% of study participants were reported achieving disease stability. - Mild adverse effects were reported in study participants, diarrhoea was the most common toxicity (55%). | (61)  NCT03408587 |
|  | None | Stage IV Melanoma | I | Completed | No outcome is reported currently. | No related publications currently.  NCT00438009 |
|  | None | Malignant Melanoma | I | Completed | No outcome is reported currently. | No related publications currently.  NCT00235482 |
|  | MA  (Ipilimumab) | Melanoma | I | Completed | - Overall, the objective response rate was reported 30% in all study participants. - The objective response rate was reported 47% in study participants receiving combination therapy. - No dose-limiting adverse effects were reported. The most prevalent mild adverse effect was pruritus (50%). | (62)   NCT02307149 |
|  | None | Melanoma | II | Completed | - Overall, the response rate was reported 28% in study participants. - No severe treatment-associated adverse effects were reported in the participants. | (63)  NCT01227551 & NCT01636882 (extension study) |
|  | None | Head & Neck Cancer | I | Terminated | No outcome is reported currently.  **The trial is terminated due to the low recruitment rate.** | No related publications currently.  NCT00832559 |
| RNA Viruses: Paramyxovirus | | | | | | |
| Measles Virus (MV-NIS) | None | Urothelial Carcinoma | I | Completed | No outcome is reported currently. | No related publications currently.  NCT03171493 |
|  | None | Medulloblastoma and Atypical Teratoid/Rhabdoid Tumour | I | Completed | No outcome is reported currently. | No related publications currently.  NCT02962167 |
|  | CT  (Cyclophosphamide) | Plasma Cell Myeloma | I, II | Completed | - 5% of study participants receiving only MV-NIS were reported achieving clinical responses. - No dose-limiting adverse effects were reported in study participants. | (64)  NCT00450814 |
|  | None | Malignant Pleural Mesothelioma | I | Completed | - Efficacy is not directly measured in this trial. - No dose-limiting toxicities were reported in study participants. | (65)  NCT01503177 |
|  | Measles Virus  (MV-CEA) | Ovarian Cancer and Peritoneal Cavity Cancer | I | Completed | - Overall, 74% of study participants were reported achieving disease stability. - No dose-limiting adverse effects were observed in study participants. Most participants were reported experiencing abdominal pain. | No related publications currently.  NCT00408590 |
|  | None | Multiple Myeloma | II | Completed | - Efficacy is not evaluated in the study participants. - Common adverse effects in study participants were metabolic disorders and neutropenia. | No related publications currently.  NCT02192775 |
|  | None | Neurofibromatosis and Peripheral Nerve Sheath Tumour | I | Recruiting | No outcome is reported currently. | (66)  NCT02700230 |
|  | MSCs | Fallopian Tube Cancer, Ovarian Cancer, and Peritoneal Cancer | I, II | Recruiting | No outcome is reported currently. | No related publications currently.  NCT02068794 |
|  | MA  (Atezolizumab) | Non-Small Cell Lung Cancer | I | Terminated | - Efficacy is not measured. - The common adverse effects observed were pruritus and chills.   **The trial is terminated due to poor recruitment.** | No related publications currently.  NCT02919449 |
| Measles Virus (MV-CEA) | Measles Virus  (MV-NIS) | Ovarian Cancer  Peritoneal Cavity Cancer | I | Completed | - Overall, 74% of study participants were reported achieving disease stability. - No dose-limiting adverse effects were observed in study participants. Most participants were reported experiencing abdominal pain. | No related publications currently.  NCT00408590 |
|  | None | Anaplastic Astrocytoma, Anaplastic Oligodendroglioma, Mixed Glioma, and Glioblastoma | I | Completed | - Overall, 91% of study participants reported achieving the best objective response. - 28% of study participants were reported experiencing mild adverse effects. The most common adverse effect was fatigue. | (67)  NCT00390299 |
| Newcastle Disease Virus  (MEDI5395) | MA  (Durvalumab) | Advanced Solid Tumours | I | Completed | No outcome is reported currently. | (68)  NCT03889275 |
| Newcastle Disease Virus  (MEDI9253) | MA  (Durvalumab) | Solid Tumours | I | Active, Not Recruiting | - 15% of study participants were reported having exceeded 2 times increment of tumoral CD8 T lymphocyte frequency - 50% of study participants were reported achieving disease stability. - Administration is feasible and safe. | (69)  NCT04613492 |

RT, Radiotherapy; CT, Chemotherapy; IT, Immunotherapy; MA, Monoclonal Antibody; GC, Glucocorticoids; SP, Surgical Procedure; TT, Targeted Therapy; HT, Hormonal Therapy; SCC, Squamous Cell Carcinoma; MSCs, Mesenchymal Stem Cells

**Supplementary Table 6.** Clinical Trials of Pelareorep, Reolysin (A) and the Use of Other Reoviruses (B) against Different Types of Malignancies

| 1. Clinical Trials with Pelareorep (Reolysin) | | | | | | | | |
| --- | --- | --- | --- | --- | --- | --- | --- | --- |
| Treatment Combination | | **Tumour Type** | | **Phase** | | **Status (Accessed June 2024)** | **Outcome (Efficacy & Safety)** | **References** |
| CT  (Paclitaxel) | | Metastatic Breast Cancer | | II | | Completed | - Overall, 24% of study participants were reported achieving objective responses. - The treatment is generally well-tolerated. The most common adverse effect reported was fever (65%). | (70)  NCT01656538 |
| None | | Malignant Glioma | | I | | Completed | - 56% of study participants were reported achieving disease stability. - The adverse effects reported were not treatment-associated. | (71)  NCT00528684 |
| None | | Osteosarcoma, Ewing Sarcoma, Malignant Fibrous Histiocytoma, Synovial Sarcoma, Fibrosarcoma, and Leiomyosarcoma | | II | | Completed | - 42% of study participants were reported achieving disease stability. - Only low-grade adverse effects were reported in a minority of study participants. | (72)  NCT00503295 |
| CT  (Gemcitabine) | | Metastatic Pancreatic Adenocarcinoma | | II | | Completed | - Overall, study participants were reported to have a clinical benefit rate of 58%. - The most common adverse effect reported among study participants was fatigue (71%). | (73)  NCT00998322 |
| CT  (Paclitaxel) | | Fallopian Tube Cancer, Ovarian Cancer and Peritoneal Cancer | | II | | Completed | - 17% of study participants reported having an overall response. - The most common adverse effect reported was fatigue (84%). | (74)  NCT01199263 |
| MA  (Pembrolizumab)  CT  (Gemcitabine,  Irinotecan,  5-Fluorouracil) | | Pancreatic Adenocarcinoma | | I | | Completed | - 40% of study participants were reported having disease stability. - Low-grade adverse effects were reported in study participants. Fever was the most frequent adverse effect (64%). | (75,76)  NCT02620423 |
| MA  (Retifanlimab) | | Triple Negative Breast Cancer | | II | | Recruiting | No outcome is reported currently. | (77)  NCT04445844 |
| CT  (FOLFOX)  MA  (Bevacizumab) | | Colorectal Cancer | | II | | Completed | - 53% of study participants reported achieving objective responses. - Febrile neutropenia was observed as the most common adverse effect in an average of 47% of patients. | (78)  NCT01622543 |
| CT  (Carboplatin,  Paclitaxel) | | Head & Neck SCC | | III | | Completed | No outcome is reported currently. | No related publications currently.  NCT01166542 |
| 1. Potential Non-human Oncolytic Reoviruses | | | | | | | | |
| Reovirus | **Strain** | | **Tumour Type** | | **Details** | | | **References** |
| ARV | ARV-PB1  ARV S1133 | | Hepatocellular carcinoma, lung adenocarcinoma, cervical adenocarcinoma, & melanoma. | | - Inducing CPE and cytolytic activity in Huh-7, Huh-7.5, Huh-7.5.1, HepG2, A549, B16-F10, and HeLa cells, including HCV-infected cells. - Inhibiting invadopodia formation in B16-F10 cells. - No significant cytotoxic effect in *ex vivo* primary liver cells. - No significant/obvious pathological damage in infected SPF Kunming mice. | | | (79-82) |
| PRV | PRV7S | | Human nasopharyngeal carcinoma | | - Inducing CPE and inhibiting the proliferation of CNE1, TWO1, HONNE1 and SUNE1 cells. - Less cytolytic and cytopathic effects were observed in non-carcinoma nasopharyngeal cells, NP69 and NP460 cells. - Not capable of establishing persistent infection. | | | (83,84) |

CT, Chemotherapy; MA, Monoclonal Antibody; SCC, Squamous Cell Carcinoma; KRAS, Kirsten rat sarcoma viral oncogene homolog; ARV, Avian orthoreovirus; ARV-PB1, avian reovirus pathobiology 1; CPE, cytopathic effect; HCV, hepatitis C virus; PRV, pteropine orthoreovirus; PRV7S, Sikamat virus.

**References**

1. Bennett, J. M., Catovsky, D., Daniel, M. T., Flandrin, G., Galton, D. A., Gralnick, H. R., and Sultan, C. (1985). Proposed revised criteria for the classification of acute myeloid leukemia. A report of the French-American-British Cooperative Group. *Annals of internal medicine*, *103*(4), 620–625. <https://doi.org/10.7326/0003-4819-103-4-620>

2. Neame, P. B., Soamboonsrup, P., Browman, G. P., Meyer, R. M., Benger, A., Wilson, W. E., Walker, I. R., Saeed, N., & McBride, J. A. (1986). Classifying acute leukemia by immunophenotyping: a combined FAB-immunologic classification of AML. *Blood*, *68*(6), 1355–1362. <https://doi.org/10.1182/blood.V68.6.1355.1355>

3. Bennett, J. M., Catovsky, D., Daniel, M. T., Flandrin, G., Galton, D. A., Gralnick, H., Sultan, C., and Cox, C. (1994). The chronic myeloid leukaemias: guidelines for distinguishing chronic granulocytic, atypical chronic myeloid, and chronic myelomonocytic leukaemia. Proposals by the French-American-British Cooperative Leukaemia Group. *British journal of haematology*, ***87***(4), 746–754. <https://doi.org/10.1111/j.1365-2141.1994.tb06734.x>

4. Catovsky, D., Matutes, E., Buccheri, V., Shetty, V., Hanslip, J., Yoshida, N., & Morilla, R. (1991). A classification of acute leukaemia for the 1990s. *Annals of hematology*, *62*(1), 16–21. <https://doi.org/10.1007/BF01714978>

5. Arber, D. A., Orazi, A., Hasserjian, R., Thiele, J., Borowitz, M. J., Le Beau, M. M., Bloomfield, C. D., Cazzola, M., and Vardiman, J. W. (2016). The 2016 revision to the World Health Organization classification of myeloid neoplasms and acute leukemia. *Blood*, ***127***(20), 2391–2405. <https://doi.org/10.1182/blood-2016-03-643544>

6. Khoury, J. D., Solary, E., Abla, O., Akkari, Y., Alaggio, R., Apperley, J. F., Bejar, R., Berti, E., Busque, L., Chan, J. K. C., Chen, W., Chen, X., Chng, W. J., Choi, J. K., Colmenero, I., Coupland, S. E., Cross, N. C. P., De Jong, D., Elghetany, M. T., Takahashi, E., … Hochhaus, A. (2022). The 5th edition of the World Health Organization Classification of Haematolymphoid Tumours: Myeloid and Histiocytic/Dendritic Neoplasms. *Leukemia*, ***36***(7), 1703–1719. <https://doi.org/10.1038/s41375-022-01613-1>

7. Appelbaum F. R. (2023). WHO, what, when, where, and why: New classification systems for acute myeloid leukemia and their impact on clinical practice. *Best practice & research. Clinical haematology*, *36*(4), 101518. <https://doi.org/10.1016/j.beha.2023.101518>

8. Loghavi S. (2024). SOHO State of the Art Updates and Next Questions-WHO Classification of Acute Myeloid Leukemia. *Clinical lymphoma, myeloma & leukemia*, *24*(11), 752–758. <https://doi.org/10.1016/j.clml.2024.05.001>

9. Chen, W., Konoplev, S., Medeiros, L. J., Koeppen, H., Leventaki, V., Vadhan-Raj, S., Jones, D., Kantarjian, H. M., Falini, B., & Bueso-Ramos, C. E. (2009). Cuplike nuclei (prominent nuclear invaginations) in acute myeloid leukemia are highly associated with FLT3 internal tandem duplication and NPM1 mutation. *Cancer*, *115*(23), 5481–5489. <https://doi.org/10.1002/cncr.24610>

10. Solal-Celigny, P., Desaint, B., Herrera, A., Chastang, C., Amar, M., Vroclans, M., Brousse, N., Mancilla, F., Renoux, M., & Bernard, J. F. (1984). Chronic myelomonocytic leukemia according to FAB classification: analysis of 35 cases. *Blood*, *63*(3), 634–638. <https://doi.org/10.1182/blood.V63.3.634.634>

11. Haznedaroğlu, İ. C., Kuzu, I., & İlhan, O. (2020). WHO 2016 Definition of Chronic Myeloid Leukemia and Tyrosine Kinase Inhibitors. *Turkish journal of haematology : official journal of Turkish Society of Haematology*, *37*(1), 42–47. <https://doi.org/10.4274/tjh.galenos.2019.2019.0241>

12. Iezza, M., Cortesi, S., Ottaviani, E., Mancini, M., Venturi, C., Monaldi, C., De Santis, S., Testoni, N., Soverini, S., Rosti, G., Cavo, M., & Castagnetti, F. (2023). Prognosis in Chronic Myeloid Leukemia: Baseline Factors, Dynamic Risk Assessment and Novel Insights. *Cells*, *12*(13), 1703. <https://doi.org/10.3390/cells12131703>

13. Markert, J. M., Razdan, S. N., Kuo, H. C., Cantor, A., Knoll, A., Karrasch, M., Nabors, L. B., Markiewicz, M., Agee, B. S., Coleman, J. M., Lakeman, A. D., Palmer, C. A., Parker, J. N., Whitley, R. J., Weichselbaum, R. R., Fiveash, J. B. and Gillespie, G. Y. (2014). A phase 1 trial of oncolytic HSV-1, G207, given in combination with radiation for recurrent GBM demonstrates safety and radiographic responses. *Molecular therapy : the journal of the American Society of Gene Therapy*, ***22***(5), 1048–1055. <https://doi.org/10.1038/mt.2014.22>

14. Markert, J. M., Medlock, M. D., Rabkin, S. D., Gillespie, G. Y., Todo, T., Hunter, W. D., Palmer, C. A., Feigenbaum, F., Tornatore, C., Tufaro, F. and Martuza, R. L. (2000). Conditionally replicating herpes simplex virus mutant, G207 for the treatment of malignant glioma: results of a phase I trial. *Gene therapy*, ***7***(10), 867–874. <https://doi.org/10.1038/sj.gt.3301205>

15. Friedman, G. K., Johnston, J. M., Bag, A. K., Bernstock, J. D., Li, R., Aban, I., Kachurak, K., Nan, L., Kang, K. D., Totsch, S., Schlappi, C., Martin, A. M., Pastakia, D., McNall-Knapp, R., Farouk Sait, S., Khakoo, Y., Karajannis, M. A., Woodling, K., Palmer, J. D., Osorio, D. S., … Gillespie, G. Y. (2021). Oncolytic HSV-1 G207 Immunovirotherapy for Pediatric High-Grade Gliomas. *The New England journal of medicine*, ***384***(17), 1613–1622. <https://doi.org/10.1056/NEJMoa2024947>

16. Sze, D. Y., Iagaru, A. H., Gambhir, S. S., De Haan, H. A. and Reid, T. R. (2012). Response to intra-arterial oncolytic virotherapy with the herpes virus NV1020 evaluated by [18F]fluorodeoxyglucose positron emission tomography and computed tomography. *Human gene therapy*, ***23***(1), 91–97. <https://doi.org/10.1089/hum.2011.141>

17. Geevarghese, S. K., Geller, D. A., de Haan, H. A., Hörer, M., Knoll, A. E., Mescheder, A., Nemunaitis, J., Reid, T. R., Sze, D. Y., Tanabe, K. K. and Tawfik, H. (2010). Phase I/II study of oncolytic herpes simplex virus NV1020 in patients with extensively pretreated refractory colorectal cancer metastatic to the liver. *Human gene therapy*, ***21***(9), 1119–1128. <https://doi.org/10.1089/hum.2010.020>

18. Wang, X., Cui, C., Si, L., Li, C., Dai, J., Mao, L., Bai, X., Chi, Z., Sheng, X., Kong, Y., Lian, B., Tang, B., Yan, X., Zhou, L., Li, S., Andtbacka, R.H.I. and Guo, J. (2021). A phase Ib clinical trial of neoadjuvant OrienX010, an oncolytic virus, in combination with toripalimab in patients with resectable stage IIIb to stage IVM1a acral melanoma. *J Clin Oncol*, ***39***, 9570. <https://doi.org/10.1200/JCO.2021.39.15_suppl.9570>

19. Andtbacka, R. H., Kaufman, H. L., Collichio, F., Amatruda, T., Senzer, N., Chesney, J., Delman, K. A., Spitler, L. E., Puzanov, I., Agarwala, S. S., Milhem, M., Cranmer, L., Curti, B., Lewis, K., Ross, M., Guthrie, T., Linette, G. P., Daniels, G. A., Harrington, K., Middleton, M. R., … Coffin, R. S. (2015). Talimogene Laherparepvec Improves Durable Response Rate in Patients With Advanced Melanoma. *Journal of clinical oncology : official journal of the American Society of Clinical Oncology*, ***33***(25), 2780–2788. <https://doi.org/10.1200/JCO.2014.58.3377>

20. Streby, K. A., Geller, J. I., Currier, M. A., Warren, P. S., Racadio, J. M., Towbin, A. J., Vaughan, M. R., Triplet, M., Ott-Napier, K., Dishman, D. J., Backus, L. R., Stockman, B., Brunner, M., Simpson, K., Spavin, R., Conner, J. and Cripe, T. P. (2017). Intratumoral Injection of HSV1716, an Oncolytic Herpes Virus, Is Safe and Shows Evidence of Immune Response and Viral Replication in Young Cancer Patients. *Clinical cancer research : an official journal of the American Association for Cancer Research*, ***23***(14), 3566–3574. <https://doi.org/10.1158/1078-0432.CCR-16-2900>

21. Danson, S. J., Conner, J., Edwards, J. G., Blyth, K. G., Fisher, P. M., Muthana, M., Salawu, A., Taylor, F., Hodgkinson, E., Joyce, P., Roman, J., Simpson, K., Graham, A., Learmonth, K. and Woll, P. J. (2020). Oncolytic herpesvirus therapy for mesothelioma - A phase I/IIa trial of intrapleural administration of HSV1716. *Lung cancer (Amsterdam, Netherlands)*, ***150***, 145–151. <https://doi.org/10.1016/j.lungcan.2020.10.007>

22. Rohaan, M. W., Stahlie, E. H. A., Franke, V., Zijlker, L. P., Wilgenhof, S., van der Noort, V., van Akkooi, A. C. J. and Haanen, J. B. A. G. (2022). Neoadjuvant nivolumab + T-VEC combination therapy for resectable early stage or metastatic (IIIB-IVM1a) melanoma with injectable disease: study protocol of the NIVEC trial. *BMC cancer*, ***22***(1), 851. <https://doi.org/10.1186/s12885-022-09896-4>

23. Tulokas, S. K. A., Kohtamäki, L. M., Mäkelä, S. P., Juteau, S., Albäck, A., Vikatmaa, P. J., Mattila, K. E., Skyttä, T. K., Koivunen, J. P., Tyynelä-Korhonen, K. and Hernberg, M. M. (2021). Isolated limb perfusion with melphalan as treatment for regionally advanced melanoma of the limbs: results of 60 patients treated in Finland during 2007-2018. *Melanoma research*, ***31***(5), 456–463. <https://doi.org/10.1097/CMR.0000000000000755>

24. Hieken, T. J., Kreidieh, F., Aedo-Lopez, V., Block, M. S., McArthur, G. A. and Amaria, R. N. (2023). Neoadjuvant Immunotherapy in Melanoma: The Paradigm Shift. *American Society of Clinical Oncology educational book. American Society of Clinical Oncology. Annual Meeting*, ***43***, e390614. <https://doi.org/10.1200/EDBK_390614>

25. Stewart, J.H., Lowe, M., Chapple, A.G., Niedzwiecki, D., Moyer, A., Bolch, E., MacLaughlan, S.D., Levine, E.A., Moaven, O., Nettu, N., Strickler, J.H. and Blazer III, D.G. (2024). Phase I trial of talimogene laherparepvec for the treatment of peritoneal surface malignancies (TEMPO). *J Clin Oncol*, ***42***, 2668. <https://doi.org/10.1200/JCO.2024.42.16_suppl.2668>

26. Yamazaki, N., Isei, T., Kiyohara, Y., Koga, H., Kojima, T., Takenouchi, T., Yokota, K., Namikawa, K., Yi, M., Keegan, A. and Fukushima, S. (2022). A phase I study of the safety and efficacy of talimogene laherparepvec in Japanese patients with advanced melanoma. *Cancer science*, ***113***(8), 2798–2806. <https://doi.org/10.1111/cas.15450>

27. Barker, C.A., D’Angelo, S.P., Steckler, A.M., Lian, M., Wasilewski, G., Lacouture, M.E., Chapman, P.B., Shoushtari, A.N. and Ariyan, C.E. (2023). A phase II randomized trial of talimogene laherparepvec (T-VEC) oncolytic immunotherapy with or without radiotherapy for patients with cutaneous metastases from solid tumors. *J Clin Oncol*, ***41***(16), 2639. <https://doi.org/10.1200/JCO.2023.41.16_suppl.2639>

28. Chesney, J. A., Puzanov, I., Collichio, F. A., Singh, P., Milhem, M. M., Glaspy, J., Hamid, O., Ross, M., Friedlander, P., Garbe, C., Logan, T., Hauschild, A., Lebbé, C., Joshi, H., Snyder, W. and Mehnert, J. M. (2023). Talimogene laherparepvec in combination with ipilimumab versus ipilimumab alone for advanced melanoma: 5-year final analysis of a multicenter, randomized, open-label, phase II trial. *Journal for immunotherapy of cancer*, ***11***(5), e006270. <https://doi.org/10.1136/jitc-2022-006270>

29. Hecht, J. R., Raman, S. S., Chan, A., Kalinsky, K., Baurain, J. F., Jimenez, M. M., Garcia, M. M., Berger, M. D., Lauer, U. M., Khattak, A., Carrato, A., Zhang, Y., Liu, K., Cha, E., Keegan, A., Bhatta, S., Strassburg, C. P. and Roohullah, A. (2023). Phase Ib study of talimogene laherparepvec in combination with atezolizumab in patients with triple negative breast cancer and colorectal cancer with liver metastases. *ESMO open*, ***8***(2), 100884. <https://doi.org/10.1016/j.esmoop.2023.100884>

30. Dummer, R., Gyorki, D. E., Hyngstrom, J., Berger, A. C., Conry, R., Demidov, L., Sharma, A., Treichel, S. A., Radcliffe, H., Gorski, K. S., Anderson, A., Chan, E., Faries, M. and Ross, M. I. (2021). Neoadjuvant talimogene laherparepvec plus surgery versus surgery alone for resectable stage IIIB-IVM1a melanoma: a randomized, open-label, phase 2 trial. *Nature medicine*, ***27***(10), 1789–1796. <https://doi.org/10.1038/s41591-021-01510-7>

31. Mastrangelo, M. J., Maguire, H. C., Jr, Eisenlohr, L. C., Laughlin, C. E., Monken, C. E., McCue, P. A., Kovatich, A. J. and Lattime, E. C. (1999). Intratumoral recombinant GM-CSF-encoding virus as gene therapy in patients with cutaneous melanoma. *Cancer gene therapy*, ***6***(5), 409–422. <https://doi.org/10.1038/sj.cgt.7700066>

32. Cripe, T. P., Ngo, M. C., Geller, J. I., Louis, C. U., Currier, M. A., Racadio, J. M., Towbin, A. J., Rooney, C. M., Pelusio, A., Moon, A., Hwang, T. H., Burke, J. M., Bell, J. C., Kirn, D. H. and Breitbach, C. J. (2015). Phase 1 study of intratumoral Pexa-Vec (JX-594), an oncolytic and immunotherapeutic vaccinia virus, in pediatric cancer patients. *Molecular therapy : the journal of the American Society of Gene Therapy*, ***23***(3), 602–608. <https://doi.org/10.1038/mt.2014.243>

33. Toulmonde, M., Cousin, S., Kind, M., Guegan, J. P., Bessede, A., Le Loarer, F., Perret, R., Cantarel, C., Bellera, C. and Italiano, A. (2022). Randomized phase 2 trial of intravenous oncolytic virus JX-594 combined with low-dose cyclophosphamide in patients with advanced soft-tissue sarcoma. *Journal of hematology & oncology*, ***15***(1), 149. <https://doi.org/10.1186/s13045-022-01370-9>

34. Heo, J., Reid, T., Ruo, L., Breitbach, C. J., Rose, S., Bloomston, M., Cho, M., Lim, H. Y., Chung, H. C., Kim, C. W., Burke, J., Lencioni, R., Hickman, T., Moon, A., Lee, Y. S., Kim, M. K., Daneshmand, M., Dubois, K., Longpre, L., Ngo, M., … Kirn, D. H. (2013). Randomized dose-finding clinical trial of oncolytic immunotherapeutic vaccinia JX-594 in liver cancer. *Nature medicine*, ***19***(3), 329–336. <https://doi.org/10.1038/nm.3089>

35. Marabelle, A., Eberst, L., Terret, C., Pilleul, F., Mastier, C., Bouhamama, A., Gilles-Afchain, L., Laurent, S., Delzano, I., Reynaud, C., Caux, C., Caux C., Garin, C., Bidaux, A.S., Perol, D., Stojkowitz, N., Homerin, M., Leenders, H. and Cassier P. (2018). A phase I dose escalation trial evaluating the impact of an in situ immunization strategy with intra-tumoral injections of Pexa-Vec in combination with ipilimumab in advanced solid tumors with injectable lesions. *Ann Oncol,* ***29***, x38. <https://doi.org/10.1093/annonc/mdy487.041>

36. Abou-Alfa, G. K., Galle, P. R., Chao, Y., Erinjeri, J., Heo, J., Borad, M. J., Luca, A., Burke, J., Pelusio, A., Agathon, D., Lusky, M., Breitbach, C., Qin, S. and Gane, E. (2023). PHOCUS: A Phase 3, Randomized, Open-Label Study of Sequential Treatment with Pexa-Vec (JX-594) and Sorafenib in Patients with Advanced Hepatocellular Carcinoma. *Liver cancer*, ***13***(3), 248–264. <https://doi.org/10.1159/000533650>

37. Holloway, R.W., Kendrick, J.E., Stephens, A., Kennard, J., Burt, J., LeBlanc, J., Sellers, K., Smith, J. and Coakley, S. (2018). Phase 1b study of oncolytic vaccinia virus GL-ONC1 in recurrent ovarian cancer (ROC). *J Clin Oncol*, **36**(15), 5577. <https://doi.org/10.1200/JCO.2018.36.15_suppl.557>

38. Mell, L. K., Brumund, K. T., Daniels, G. A., Advani, S. J., Zakeri, K., Wright, M. E., Onyeama, S. J., Weisman, R. A., Sanghvi, P. R., Martin, P. J. and Szalay, A. A. (2017). Phase I Trial of Intravenous Oncolytic Vaccinia Virus (GL-ONC1) with Cisplatin and Radiotherapy in Patients with Locoregionally Advanced Head and Neck Carcinoma. *Clinical cancer research : an official journal of the American Association for Cancer Research*, ***23***(19), 5696–5702. <https://doi.org/10.1158/1078-0432.CCR-16-3232>

39. Lauer, U. M., Schell, M., Beil, J., Berchtold, S., Koppenhöfer, U., Glatzle, J., Königsrainer, A., Möhle, R., Nann, D., Fend, F., Pfannenberg, C., Bitzer, M. and Malek, N. P. (2018). Phase I Study of Oncolytic Vaccinia Virus GL-ONC1 in Patients with Peritoneal Carcinomatosis. *Clinical cancer research : an official journal of the American Association for Cancer Research*, ***24***(18), 4388–4398. <https://doi.org/10.1158/1078-0432.CCR-18-0244>

40. Manyam, M., Stephens, A. J., Kennard, J. A., LeBlanc, J., Ahmad, S., Kendrick, J. E. and Holloway, R. W. (2021). A phase 1b study of intraperitoneal oncolytic viral immunotherapy in platinum-resistant or refractory ovarian cancer. *Gynecologic oncology*, ***163***(3), 481–489. <https://doi.org/10.1016/j.ygyno.2021.10.069>

41. Hajda, J., Leuchs, B., Angelova, A. L., Frehtman, V., Rommelaere, J., Mertens, M., Pilz, M., Kieser, M., Krebs, O., Dahm, M., Huber, B., Engeland, C. E., Mavratzas, A., Hohmann, N., Schreiber, J., Jäger, D., Halama, N., Sedlaczek, O., Gaida, M. M., Daniel, V., … Ungerechts, G. (2021). Phase 2 Trial of Oncolytic H-1 Parvovirus Therapy Shows Safety and Signs of Immune System Activation in Patients With Metastatic Pancreatic Ductal Adenocarcinoma. *Clinical cancer research : an official journal of the American Association for Cancer Research*, ***27***(20), 5546–5556. <https://doi.org/10.1158/1078-0432.CCR-21-1020>

42. Geletneky, K., Hajda, J., Angelova, A. L., Leuchs, B., Capper, D., Bartsch, A. J., Neumann, J. O., Schöning, T., Hüsing, J., Beelte, B., Kiprianova, I., Roscher, M., Bhat, R., von Deimling, A., Brück, W., Just, A., Frehtman, V., Löbhard, S., Terletskaia-Ladwig, E., Fry, J., … Rommelaere, J. (2017). Oncolytic H-1 Parvovirus Shows Safety and Signs of Immunogenic Activity in a First Phase I/IIa Glioblastoma Trial. *Molecular therapy : the journal of the American Society of Gene Therapy*, ***25***(12), 2620–2634. <https://doi.org/10.1016/j.ymthe.2017.08.016>

43. Zamarin, D., Odunsi, K., Zsiros, E., Slomovitz, B.M., Pimentel, A., Duska, L.R., Reilley, M., Nemunaitis, J.J., Hamouda, D.M., Patel, H., Segal, N.H., Pesci, G., Holland, A., Shohara, L., Schwarzenberger, P.O., Ricciardi, T., Macri, M.J., Ryan, A., Jaderberg, M.E. and Venhaus, R.R. (2022). Study to evaluate intraperitoneal (IP) ONCOS-102 with systemic durvalumab in patients with peritoneal disease who have epithelial ovarian (OC) or metastatic colorectal cancer (CRC): Phase 2 results*. J Clin Oncol,* ***40***(16), 2600. <https://doi.org/10.1200/JCO.2022.40.16_suppl.260>

44. Shoushtari, A. N., Olszanski, A. J., Nyakas, M., Hornyak, T. J., Wolchok, J. D., Levitsky, V., Kuryk, L., Hansen, T. B. and Jäderberg, M. (2023). Pilot Study of ONCOS-102 and Pembrolizumab: Remodeling of the Tumor Microenvironment and Clinical Outcomes in Anti-PD-1-Resistant Advanced Melanoma. *Clinical cancer research : an official journal of the American Association for Cancer Research*, ***29***(1), 100–109. <https://doi.org/10.1158/1078-0432.CCR-22-2046>

45. Ranki, T., Pesonen, S., Hemminki, A., Partanen, K., Kairemo, K., Alanko, T., Lundin, J., Linder, N., Turkki, R., Ristimäki, A., Jäger, E., Karbach, J., Wahle, C., Kankainen, M., Backman, C., von Euler, M., Haavisto, E., Hakonen, T., Heiskanen, R., Jaderberg, M., … Joensuu, T. (2016). Phase I study with ONCOS-102 for the treatment of solid tumors - an evaluation of clinical response and exploratory analyses of immune markers. *Journal for immunotherapy of cancer*, ***4***, 17. <https://doi.org/10.1186/s40425-016-0121-5>

46. Ruano, D., López-Martín, J. A., Moreno, L., Lassaletta, Á., Bautista, F., Andión, M., Hernández, C., González-Murillo, Á., Melen, G., Alemany, R., Madero, L., García-Castro, J. and Ramírez, M. (2020). First-in-Human, First-in-Child Trial of Autologous MSCs Carrying the Oncolytic Virus Icovir-5 in Patients with Advanced Tumors. *Molecular therapy : the journal of the American Society of Gene Therapy*, ***28***(4), 1033–1042. <https://doi.org/10.1016/j.ymthe.2020.01.019>

47. Lang, F.F., Conrad, C., Gomez-Manzano, C., Yung, W.K.A., Sawaya, R., Weinberg, J.S., Prabhu, S.S., Rao, G., Fuller, G.N., Aldape, K.D., Gumin, J., Vence, L.M., Wistuba, I., Rodriguez-Canales, J., Villalobos, P.A., Dirven, C.M.F., Tejada, S., Valle, R.D., Alonso, M.M., Ewald, B., Peterkin, J.J., Tufaro, F. and Fueyo, J. (2018). Phase I Study of DNX-2401 (Delta-24-RGD) Oncolytic Adenovirus: Replication and Immunotherapeutic Effects in Recurrent Malignant Glioma. *J Clin Oncol*, ***36***(14), 1419-1427. <https://doi.org/10.1200/JCO.2017.75.8219>

48. Nassiri, F., Patil, V., Yefet, L.S., Singh, O., Liu, J., Dang, R.M.A., Yamaguchi, T.N., Daras, M., Cloughesy, T.F., Colman, H., Kumthekar, P.U., Chen, C.C., Aiken, R., Groves, M.D., Ong, S.S., Ramakrishna, R., Vogelbaum, M.A., Khagi S., Kaley, T., Melear, J.M., Peereboom, D.M., Rodriguez, A., Yankelevich, M., Nair, S.G., Puduvalli, V.K., Aldape, K., Gao, A., López-Janeiro, A., de Andrea, C.E., Alonso, M.M., Boutros, P., Robbins, J., Mason, W.P., Sonabend, A.M., Stupp, R., Fueyo, J., Gomez-Manzano, C., Lang, F.F. and Zadeh, G*.* (2023). Oncolytic DNX-2401 virotherapy plus pembrolizumab in recurrent glioblastoma: a phase 1/2 trial. *Nature medicine,* ***29***, 1370–1378. <https://doi.org/10.1038/s41591-023-02347-y>

49. Chen, S. R., Chen, M. M., Ene, C., Lang, F. F. and Kan, P. (2022). Perfusion-guided endovascular super-selective intra-arterial infusion for treatment of malignant brain tumors. *Journal of neurointerventional surgery*, ***14***(6), 533–538. <https://doi.org/10.1136/neurintsurg-2021-018190>

50. Garcia-Carbonero, R., Bazan-Peregrino, M., Gil-Martín, M., Álvarez, R., Macarulla, T., Riesco-Martinez, M. C., Verdaguer, H., Guillén-Ponce, C., Farrera-Sal, M., Moreno, R., Mato-Berciano, A., Maliandi, M. V., Torres-Manjon, S., Costa, M., Del Pozo, N., Martínez de Villarreal, J., Real, F. X., Vidal, N., Capella, G., Alemany, R., … Salazar, R. (2022). Phase I, multicenter, open-label study of intravenous VCN-01 oncolytic adenovirus with or without nab-paclitaxel plus gemcitabine in patients with advanced solid tumors. *Journal for immunotherapy of cancer*, ***10***(3), e003255. <https://doi.org/10.1136/jitc-2021-003255>

51. Tanyi, J.L., O’Hara, M.H., Hexner, E., Marshall, A., Jadlowsky, J., Ferrara, M., Farrelly, O., Runkle, A., Chew, A., Dowd, E., Gonzalez, V., Fraietta, J.A., Plesa, G., Sheppard, N.C. and June, C.H. (2023). 671 Phase 1 trial of human chimeric antigen receptor modified T cells (huCART-meso) administered in combination with oncolytic virus VCN-01 in patients with pancreatic and ovarian cancer. *Journal for ImmunoTherapy of Cancer*, ***11***. <https://doi.org/10.1136/jitc-2023-SITC2023.0671>

52. Packiam, V. T., Lamm, D. L., Barocas, D. A., Trainer, A., Fand, B., Davis, R. L., 3rd, Clark, W., Kroeger, M., Dumbadze, I., Chamie, K., Kader, A. K., Curran, D., Gutheil, J., Kuan, A., Yeung, A. W. and Steinberg, G. D. (2018). An open label, single-arm, phase II multicenter study of the safety and efficacy of CG0070 oncolytic vector regimen in patients with BCG-unresponsive non-muscle-invasive bladder cancer: Interim results. *Urologic oncology*, ***36***(10), 440–447. <https://doi.org/10.1016/j.urolonc.2017.07.005>

53. Li, R., Spiess, P.E., Sexton, W.J., Poch, M.A., Zemp, L., Pow-Sang, J.M., Zhang, J., Dhillon, J., Borjas, G., Naidu, S.U., Dorman, D., Burke, J.M., Mule, J.J., Conejo-Garcia, J. and Jain, R.K. (2023). Phase Ib neoadjuvant CG0070 and nivolumab (N) for cisplatin (C)-ineligible muscle invasive bladder cancer (MIBC). *J Clin Oncol,* ***41***(16), e16613. <https://doi.org/10.1200/JCO.2023.41.16_suppl.e16613>

54. Beasley, G. M., Nair, S. K., Farrow, N. E., Landa, K., Selim, M. A., Wiggs, C. A., Jung, S. H., Bigner, D. D., True Kelly, A., Gromeier, M. and Salama, A. K. (2021). Phase I trial of intratumoral PVSRIPO in patients with unresectable, treatment-refractory melanoma. *Journal for immunotherapy of cancer*, ***9***(4), e002203. <https://doi.org/10.1136/jitc-2020-002203>

55. Desjardins, A., Gromeier, M., Herndon, J. E., 2nd, Beaubier, N., Bolognesi, D. P., Friedman, A. H., Friedman, H. S., McSherry, F., Muscat, A. M., Nair, S., Peters, K. B., Randazzo, D., Sampson, J. H., Vlahovic, G., Harrison, W. T., McLendon, R. E., Ashley, D. and Bigner, D. D. (2018). Recurrent Glioblastoma Treated with Recombinant Poliovirus. *The New England journal of medicine*, ***379***(2), 150–161. <https://doi.org/10.1056/NEJMoa1716435>

56. Thompson, E.M., Landi, D., Brown, M. C., Friedman, H.S., McLendon, R., Herndon, J.E., 2nd, Buckley, E., Bolognesi, D.P., Lipp, E., Schroeder, K., Becher, O.J., Friedman, A.H., McKay, Z., Walter, A., Threatt, S., Jaggers, D., Desjardins, A., Gromeier, M., Bigner, D.D. and Ashley, D.M. (2023). Recombinant polio-rhinovirus immunotherapy for recurrent paediatric high-grade glioma: a phase 1b trial. *The Lancet. Child & adolescent health*, ***7***(7), 471–478. <https://doi.org/10.1016/S2352-4642(23)00031-7>

57. Burke, M.J., Ahern, C., Weigel, B.J., Poirier, J.T., Rudin, C.M., Chen, Y., Cripe, T.P., Bernhardt, M.B. and Blaney, S.M. (2015). Phase I trial of Seneca Valley Virus (NTX-010) in children with relapsed/refractory solid tumors: a report of the Children's Oncology Group. *Pediatric blood & cancer*, ***62***(5), 743–750. <https://doi.org/10.1002/pbc.25269>

58. Schenk, E.L., Mandrekar, S.J., Dy, G.K., Aubry, M.C., Tan, A.D., Dakhil, S.R., Sachs, B.A., Nieva, J.J., Bertino, E., Lee Hann, C., Schild, S.E., Wadsworth, T.W., Adjei, A.A. and Molina, J.R. (2020). A Randomized Double-Blind Phase II Study of the Seneca Valley Virus (NTX-010) versus Placebo for Patients with Extensive-Stage SCLC (ES SCLC) Who Were Stable or Responding after at Least Four Cycles of Platinum-Based Chemotherapy: North Central Cancer Treatment Group (Alliance) N0923 Study. *Journal of thoracic oncology : official publication of the International Association for the Study of Lung Cancer*, ***15***(1), 110–119. <https://doi.org/10.1016/j.jtho.2019.09.083>

59. Annels, N. E., Mansfield, D., Arif, M., Ballesteros-Merino, C., Simpson, G. R., Denyer, M., Sandhu, S. S., Melcher, A. A., Harrington, K. J., Davies, B., Au, G., Grose, M., Bagwan, I., Fox, B., Vile, R., Mostafid, H., Shafren, D. and Pandha, H. S. (2019). Phase I Trial of an ICAM-1-Targeted Immunotherapeutic-Coxsackievirus A21 (CVA21) as an Oncolytic Agent Against Non Muscle-Invasive Bladder Cancer. *Clinical cancer research : an official journal of the American Association for Cancer Research*, ***25***(19), 5818–5831. <https://doi.org/10.1158/1078-0432.CCR-18-4022>

60. Rudin, C. M., Pandha, H. S., Zibelman, M., Akerley, W. L., Harrington, K. J., Day, D., Hill, A. G., O'Day, S. J., Clay, T. D., Wright, G. M., Jennens, R. R., Gerber, D. E., Rosenberg, J. E., Ralph, C., Campbell, D. C., Curti, B. D., Merchan, J. R., Ren, Y., Schmidt, E. V., Guttman, L., … Gupta, S. (2023). Phase 1, open-label, dose-escalation study on the safety, pharmacokinetics, and preliminary efficacy of intravenous Coxsackievirus A21 (V937), with or without pembrolizumab, in patients with advanced solid tumors. *Journal for immunotherapy of cancer*, ***11***(1), e005007. <https://doi.org/10.1136/jitc-2022-005007>

61. Lutzky, J., Sullivan, R. J., Cohen, J. V., Ren, Y., Li, A. and Haq, R. (2023). Phase 1b study of intravenous coxsackievirus A21 (V937) and ipilimumab for patients with metastatic uveal melanoma. *Journal of cancer research and clinical oncology*, ***149***(9), 6059–6066. <https://doi.org/10.1007/s00432-022-04510-3>

62. Curti, B. D., Richards, J., Hyngstrom, J. R., Daniels, G. A., Faries, M., Feun, L., Margolin, K. A., Hallmeyer, S., Grose, M., Zhang, Y., Li, A. and Andtbacka, R. H. I. (2022). Intratumoral oncolytic virus V937 plus ipilimumab in patients with advanced melanoma: the phase 1b MITCI study. *Journal for immunotherapy of cancer*, ***10***(12), e005224. <https://doi.org/10.1136/jitc-2022-005224>

63. Andtbacka, R. H. I., Curti, B., Daniels, G. A., Hallmeyer, S., Whitman, E. D., Lutzky, J., Spitler, L. E., Zhou, K., Bommareddy, P. K., Grose, M., Wang, M., Wu, C. and Kaufman, H. L. (2021). Clinical Responses of Oncolytic Coxsackievirus A21 (V937) in Patients With Unresectable Melanoma. *Journal of clinical oncology : official journal of the American Society of Clinical Oncology*, ***39***(34), 3829–3838. <https://doi.org/10.1200/JCO.20.03246>

64. Dispenzieri, A., Tong, C., LaPlant, B., Lacy, M. Q., Laumann, K., Dingli, D., Zhou, Y., Federspiel, M. J., Gertz, M. A., Hayman, S., Buadi, F., O'Connor, M., Lowe, V. J., Peng, K. W. and Russell, S. J. (2017). Phase I trial of systemic administration of Edmonston strain of measles virus genetically engineered to express the sodium iodide symporter in patients with recurrent or refractory multiple myeloma. *Leukemia*, ***31***(12), 2791–2798. <https://doi.org/10.1038/leu.2017.120>

65. Peikert, T., Mandrekar, S., Mansfield, A., Keulen, V.V., Albelda, S., Aderca, S., Carlson, S., Dietz, A., Gustafson, M., Kratzke, R., Lowe V., Maldonado, F., Molina, J., Patel, M., Roden, A., Sun, J., Tan, A., Tippmann-Peikert, M. and Galanis, E. (2017). OA13.07 Intrapleural Modified Vaccine Strain Measles Virus Therapy for Patients with Malignant Pleural Mesothelioma. *J Thorac Oncol,* ***12***(1), S296. <https://doi.org/10.1016/j.jtho.2016.11.305>

66. Hassan, A., Pestana, R. C. and Parkes, A. (2021). Systemic Options for Malignant Peripheral Nerve Sheath Tumors. *Current treatment options in oncology*, ***22***(4), 33. <https://doi.org/10.1007/s11864-021-00830-7>

67. Galanis, E., Dooley, K. E., Keith Anderson, S., Kurokawa, C. B., Carrero, X. W., Uhm, J. H., Federspiel, M. J., Leontovich, A. A., Aderca, I., Viker, K. B., Hammack, J. E., Marks, R. S., Robinson, S. I., Johnson, D. R., Kaufmann, T. J., Buckner, J. C., Lachance, D. H., Burns, T. C., Giannini, C., Raghunathan, A., … Parney, I. F. (2024). Carcinoembryonic antigen-expressing oncolytic measles virus derivative in recurrent glioblastoma: a phase 1 trial. *Nature communications*, ***15***(1), 493. <https://doi.org/10.1038/s41467-023-43076-7>

68. Dy, G.K., Davar, D., Galanis, E., Townsley, D., Karanovic, D., Schwaederle, M., Kelly, B., Zamarin, D., Borad, M. and Harrington, K. (2020). Abstract CT244: A phase 1 study of IV MEDI5395, an oncolytic virus, in combination with durvalumab in patients with advanced solid tumors. *Cancer Research*, ***80***(16), CT244. <https://doi.org/10.1158/1538-7445.AM2020-CT244>

69. Postel-Vinay, S., Cosaert, J., Hattersley, M., Phillips, M., Tu, E., Durham, N., Agrawal, S., Martin P.L., Chandrasekar, P., Ouali, K. and Arnaldez, F.I. (2023). 109O Phase I study of MEDI9253, a recombinant Newcastle Disease Virus encoding interleukin-12, in combination with durvalumab in participants with select advanced/metastatic solid tumors. *Ann Oncol*, ***8***(1), 2. doi: <https://doi.org/10.1016/j.esmoop.2023.101161>

70. Bernstein, V., Ellard, S. L., Dent, S. F., Tu, D., Mates, M., Dhesy-Thind, S. K., Panasci, L., Gelmon, K. A., Salim, M., Song, X., Clemons, M., Ksienski, D., Verma, S., Simmons, C., Lui, H., Chi, K., Feilotter, H., Hagerman, L. J., and Seymour, L. (2018). A randomized phase II study of weekly paclitaxel with or without pelareorep in patients with metastatic breast cancer: final analysis of Canadian Cancer Trials Group IND.213.. *Breast cancer research and treatment*, ***167***(2), 485–493. <https://doi.org/10.1007/s10549-017-4538-4>

71. Kicielinski, K. P., Chiocca, E. A., Yu, J. S., Gill, G. M., Coffey, M. and Markert, J. M. (2014). Phase 1 clinical trial of intratumoral reovirus infusion for the treatment of recurrent malignant gliomas in adults. *Molecular therapy : the journal of the American Society of Gene Therapy*, ***22***(5), 1056–1062. <https://doi.org/10.1038/mt.2014.21>

72. Mita, A.C., Sankhala, K., Sarantopoulos, J., Carmona, J., Okuno, S. and Goel, S. (2009). A phase II study of intravenous (IV) wild-type reovirus (Reolysin) in the treatment of patients with bone and soft tissue sarcomas metastatic to the lung. *J Clin Oncol,* ***27***(15), 10524. <https://doi.org/10.1200/jco.2009.27.15_suppl.10524>

73. Mahalingam, D., Goel, S., Aparo, S., Patel Arora, S., Noronha, N., Tran, H., Chakrabarty, R., Selvaggi, G., Gutierrez, A., Coffey, M., Nawrocki, S. T., Nuovo, G. and Mita, M. M. (2018). A Phase II Study of Pelareorep (REOLYSIN^®^) in Combination with Gemcitabine for Patients with Advanced Pancreatic Adenocarcinoma. *Cancers*, ***10***(6), 160. <https://doi.org/10.3390/cancers10060160>

74. Cohn, D. E., Sill, M. W., Walker, J. L., O'Malley, D., Nagel, C. I., Rutledge, T. L., Bradley, W., Richardson, D. L., Moxley, K. M. and Aghajanian, C. (2017). Randomized phase IIB evaluation of weekly paclitaxel versus weekly paclitaxel with oncolytic reovirus (Reolysin®) in recurrent ovarian, tubal, or peritoneal cancer: An NRG Oncology/Gynecologic Oncology Group study. *Gynecologic oncology*, ***146***(3), 477–483. <https://doi.org/10.1016/j.ygyno.2017.07.135>

75. Mahalingam, D., Fountzilas, C., Moseley, J. L., Noronha, N., Cheetham, K., Dzugalo, A. D., Nuovo, G. J., Gutierrez, A. A., and Arora, S. P. (2017). A study of pelareorep in combination with pembrolizumab and chemotherapy in patients (pts) with relapsed metastatic adenocarcinoma of the pancreas (MAP). *Journal of Clinical Oncology*, ***35***(15_suppl), e15753. <https://doi.org/10.1200/JCO.2017.35.15_suppl.e15753>

76. Mahalingam, D., Wilkinson, G. A., Eng, K. H., Fields, P., Raber, P., Moseley, J. L., Cheetham, K., Coffey, M., Nuovo, G., Kalinski, P., Zhang, B., Arora, S. P., and Fountzilas, C. (2020). Pembrolizumab in Combination with the Oncolytic Virus Pelareorep and Chemotherapy in Patients with Advanced Pancreatic Adenocarcinoma: A Phase Ib Study. *Clinical cancer research: an official journal of the American Association for Cancer Research*, ***26***(1), 71–81. <https://doi.org/10.1158/1078-0432.CCR-19-2078>

77. George, M., Williams, N., Omene, C., Chan, N., Lu, S.E., Tang, D., Wilkinson, G., Ganesan, S. and Toppmeyer, D. (2022). Abstract OT2-25-01: Irene study: Phase 2 study of Incmga00012 and the oncolytic virus pelareorep in metastatic triple negative breast cancer. *Cancer Research*, ***82***(4), OT2-25-01. <https://doi.org/10.1158/1538-7445.SABCS21-OT2-25-01>

78. Jonker, D. J., Tang, P. A., Kennecke, H., Welch, S. A., Cripps, M. C., Asmis, T., Chalchal, H., Tomiak, A., Lim, H., Ko, Y. J., Chen, E. X., Alcindor, T., Goffin, J. R., Korpanty, G. J., Feilotter, H., Tsao, M. S., Theis, A., Tu, D. and Seymour, L. (2018). A Randomized Phase II Study of FOLFOX6/Bevacizumab With or Without Pelareorep in Patients With Metastatic Colorectal Cancer: IND.210, a Canadian Cancer Trials Group Trial. *Clinical colorectal cancer*, ***17***(3), 231–239.e7. <https://doi.org/10.1016/j.clcc.2018.03.001>

79. Kozak, R. A., Hattin, L., Biondi, M. J., Corredor, J. C., Walsh, S., Xue-Zhong, M., Manuel, J., McGilvray, I. D., Morgenstern, J., Lusty, E., Cherepanov, V., McBey, B. A., Leishman, D., Feld, J. J., Bridle, B., and Nagy, É. (2017). Replication and Oncolytic Activity of an Avian Orthoreovirus in Human Hepatocellular Carcinoma Cells. *Viruses*, ***9***(4), 90. <https://doi.org/10.3390/v9040090>

80. Cai, R., Meng, G., Li, Y., Wang, W., Diao, Y., Zhao, S., Feng, Q., and Tang, Y. (2019). The oncolytic efficacy and safety of avian reovirus and its dynamic distribution in infected mice. *Experimental biology and medicine (Maywood, N.J.)*, ***244***(12), 983–991. <https://doi.org/10.1177/1535370219861928>

81. Hsu, C. Y., Huang, J. W., Huang, W. R., Chen, I. C., Chen, M. S., Liao, T. L., Chang, Y. K., Munir, M., and Liu, H. J. (2023). Oncolytic Avian Reovirus σA-Modulated Upregulation of the HIF-1α/C-myc/glut1 Pathway to Produce More Energy in Different Cancer Cell Lines Benefiting Virus Replication. *Viruses*, ***15***(2), 523. <https://doi.org/10.3390/v15020523>

82. Hsu, C., Li, J., Yang, E., Liao, T., Wen, H., Tsai, P., Ju, T., Lye, L., Nielsen, B. L., and Liu, H. (2024). The Oncolytic Avian Reovirus p17 Protein Inhibits Invadopodia Formation in Murine Melanoma Cancer Cells by Suppressing the FAK/Src Pathway and the Formation of theTKs5/NCK1 Complex. *Viruses*, ***16***(7), 1153. <https://doi.org/10.3390/v16071153>

83. Loh, A., Shaibie, N. A., Chan, P. Q., Leong, P. P., Segeran, S., Tan, B. K., Wong, S. T., Koh, R. Y., Chye, S. M., and Voon, K. (2022). Pteropine Orthoreovirus, PRV7S (Sikamat Virus) Demonstrates Oncolysis in Nasopharyngeal Carcinoma Cell Lines. *Frontiers in bioscience (Landmark edition)*, ***27***(4), 138. <https://doi.org/10.31083/j.fbl2704138>

84. Loh, M. K. A., Siew, Z. Y., Leong, P. P., Koh, R. Y., Chye, S. M., Wong, S. T. and Voon, K. (2023). Pteropine Orthoreovirus (PRV7S) does not Establish Persistent Infection in NP460 Cells but with Inflammatory Response Triggered. *Biomedical and environmental sciences : BES*, ***36***(9), 886–891. <https://doi.org/10.3967/bes2023.114>
